# Endogenous control genes in complex vascular tissue samples

**DOI:** 10.1186/1471-2164-10-516

**Published:** 2009-11-10

**Authors:** Lasse Folkersen, Sanela Kurtovic, Anton Razuvaev, Hanna E Agardh, Anders Gabrielsen, Gabrielle Paulsson-Berne

**Affiliations:** 1 Cardiovasular Research Unit, Center for Molecular Medicine, Department of Medicine, Karolinska Institute, Stockholm, Sweden

## Abstract

**Background:**

Gene expression microarrays and real-time PCR are common methods used to measure mRNA levels. Each method has a fundamentally different approach of normalization between samples. Relative quantification of gene expression using real-time PCR is often done using the 2^(-ΔΔCt) method, in which the normalization is performed using one or more endogenous control genes. The choice of endogenous control gene is often arbitrary or bound by tradition. We here present an analysis of the differences in expression results obtained with microarray and real-time PCR, dependent on different choices of endogenous control genes.

**Results:**

In complex tissue, microarray data and real-time PCR data show the best correlation when endogenous control genes are omitted and the normalization is done relative to total RNA mass, as measured before reverse transcription.

**Conclusion:**

We have found that for real-time PCR in heterogeneous tissue samples, it may be a better choice to normalize real-time PCR Ct values to the carefully measured mass of total RNA than to use endogenous control genes. We base this conclusion on the fact that total RNA mass normalization of real-time PCR data shows better correlation to microarray data. Because microarray data use a different normalization approach based on a larger part of the transcriptome, we conclude that omitting endogenous control genes will give measurements more in accordance with actual concentrations.

## Background

Real-time PCR is a sensitive method for expression analysis widely used for both cell culture and complex tissues. Relative quantification of mRNA levels using real-time PCR data is commonly done using the 2^(-ΔΔCt) method [1]. A central idea of this method is the use of an endogenous control for normalization, a so-called housekeeping gene. The aim of this normalization is to correct for different amounts of starting material of RNA or differences in the cDNA synthesis efficiency. Commonly used selection criteria for housekeeping genes are genes with the least amount of variance across all samples and genes that show no trends of change in relation to sample parameters of interest. However, because of lack of methods to determine low variance - other than real-time PCR itself - the selection of endogenous controls often comes precariously close to circular reasoning. Vandesompele and coworkers have suggested methods to circumvent this, through the iterative calculation of pairwise correlations with other potential endogenous control genes and removal of the most deviating candidates [2].

To investigate the merit of these endogenous control selection methods, we analyzed gene expression using different real-time PCR normalization setups and compared it with gene expression obtained using the fundamentally different approach of expression microarray measurements. The method of real-time PCR is often used as a gold standard with which to validate findings from expression microarray experiments [3-5]. This view, that real-time PCR is a gold standard, might be true when looking at individual genes. However, the specific question of between-sample normalization is usually covered by measuring one or a few supposedly constant endogenous control genes. With microarrays, on the other hand, the large number of measured genes in microarrays gives a much broader base from which to address sample variation and normalization issues. We therefore propose to investigate the specific issue of real-time PCR normalization, using correlation to microarray data as our primary metric.

Herein, we present an analysis of 87 human carotid plaque samples, for which gene expression data have been obtained with Affymetrix HG-U133 plus 2.0 arrays and for 15 target genes using TaqMan real-time PCR. The plaque tissue is typically of a heterogeneous character, containing diverse populations of leukocytes, endothelial cells, and smooth muscle cells in various proportions. Finding and validating a set of control genes that are stable across samples under these conditions is therefore essential for accurate measurement of gene expression levels.

## Results and Discussion

### Selection of endogenous control genes

We made a definition of established endogenous controls as genes available commercially, such as from Applied Biosystems. At the time of the analysis, they were: ACTB, B2 M, GAPDH, GUSB, HPRT1, PGK1, PPIA, RPLP0, TBP, and TFRC. From these, GAPDH, B2 M, PPIA, RPLP0, and TBP were selected as endogenous control candidates. They were selected, as described in methods.

The Ct value of each of these genes was submitted to the geNorm plugin for investigation of the stability index. The most stable gene pair was GAPDH and RPLP0. In order of decreasing stability, they were followed by TBP, PPIA, and B2 M. The exact definition of this method of classification is further described by Vandesompele et al. [2]. Briefly, the two most stable genes are identified by calculating expression ratios, over all samples, for all pairwise combinations of genes. For each pair of genes, the standard deviation over all samples is calculated, and for each gene, the mean of all standard deviations is calculated. Genes for which this value is highest are iteratively eliminated--in other words, the algorithm searches for genes that show the same expression profiles across all samples.

### Comparison of real-time PCR expression data with microarray expression data

**Figure 1:**
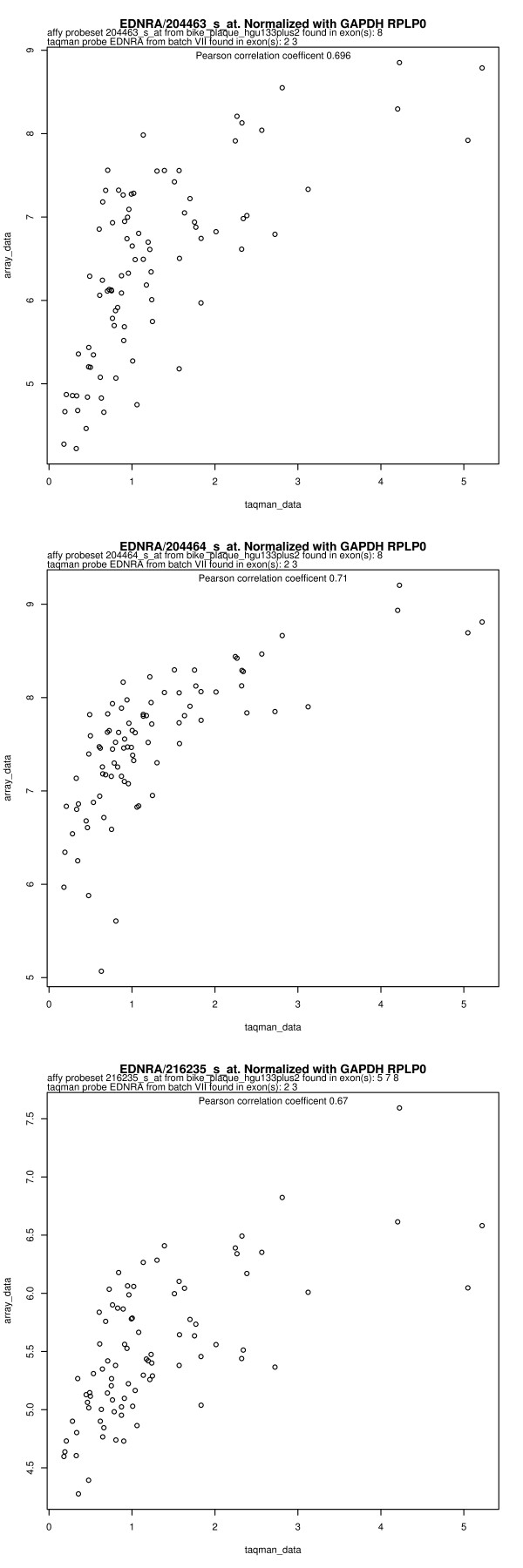
**Correlation between real-time PCR data and microarray data for EDNRA**. Real-time PCR data has been analyzed using the ΔΔCt method with GAPDH and RPLP0 as endogenous controls. Microarray data are shown for each of the three probe sets in EDNRA. The values were obtained using the Affymetrix Power Tools implementation of RMA normalization [8]. Exon location information for TaqMan probes is from the Applied Biosystems webpage. Exon location for microarray probe sets was obtained as described in methods.

**Figure 2:**
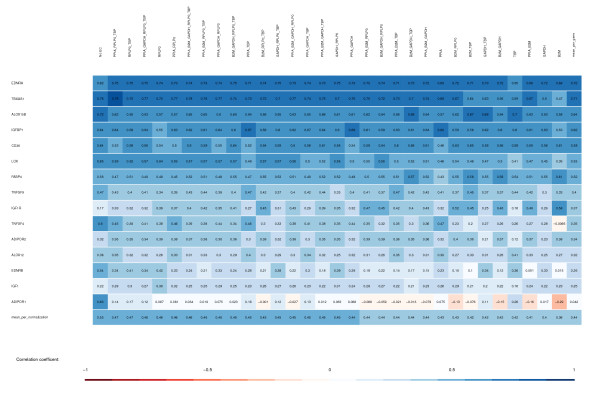
**Summary of Pearson correlation coefficients between microarray data and real-time PCR data**. The 32 possible combinations containing one or more of 5 endogenous controls are shown as columns. All genes of interest are shown as rows. The color scale for the correlation coefficient is shown below. Rows are sorted top to bottom by mean correlation coefficient across all endogenous control combinations. Columns are sorted left to right by mean correlation coefficient across all genes. As a simplification, microarray data for genes with more than one probe set are taken as the per-sample mean value of all probe sets. Creating the same figure with values per probe set would give a figure with 34 rows (one for each probe set in the genes of interest), with slightly different correlation coefficients but would not change the sorting of the columns overall. Exact distribution using per-probe-set analysis can be extracted from Additional file 1. Real-time PCR data on LOX and ALOX12 has large deviations on replicate values as described in the text and can be omitted without changing the results.

Real-time PCR data were obtained for 15 different genes of interest in addition to the 5 candidate endogenous controls. We compared the measurement of gene expression using these two methods of quantification. For each gene, this was done by creating a scatter plot, such as the example shown in Figure 1. Scatter plots for all combinations of genes, probe sets, and endogenous control combinations are found in Additional file 1. Pearson correlation coefficients were calculated for all of these combinations, and a summary can be seen in Figure 2. This figure prompts two discussions: a row-wise discussion of the differences in correlations between different genes and a column-wise discussion of the differences in correlations between different choices of endogenous controls.

### Differences in correlations between genes

Some genes show good correlation between real-time PCR measurements and microarray measurements, and others do not. These differences can be explained biologically and technically.

It was investigated whether there was any systematic technical bias of the real-time PCR-to-microarray correlation. This was done by comparing the correlation metric to the mean absolute expression level and the standard deviation, both for microarray values and real-time PCR values. This is shown as scatter plots in Additional file 2, and no patterns could be identified.

Technical imprecision in individual measurements can also be a problem. All microarray scans were subjected to standard quality control measures, as detailed in the methods section, in order to exclude problematic samples. For real-time PCR, replicate measurements can clarify if deviance is a result of technical imprecision. A technical variation threshold is described in methods. For most genes, there were only a few measurements (< 10%) with technical variation above this threshold. The exception was ALOX12, which had 69 samples with coefficients of variance above this threshold. ALOX12 was present in very low quantities, with a mean Ct of 37.08 (Additional file 3). The discrepancy between measurements of this gene is therefore concluded to be due to technical imprecision in real-time PCR, as it is reflected in the lack of correlation between microarray and real-time PCR data. The data are included in the analysis to show the effects of technical imprecision, but its removal does not change the final conclusion, as specified in the sensitivity analysis in the methods section. In addition to the threshold values in the bottom of Additional file 4, we have included the raw data from the real-time PCR measurements as Additional file 3. This threshold could possibly be used as a measure of comparison for use in other experiments that do not have microarray data to compare with.

**Figure 3:**
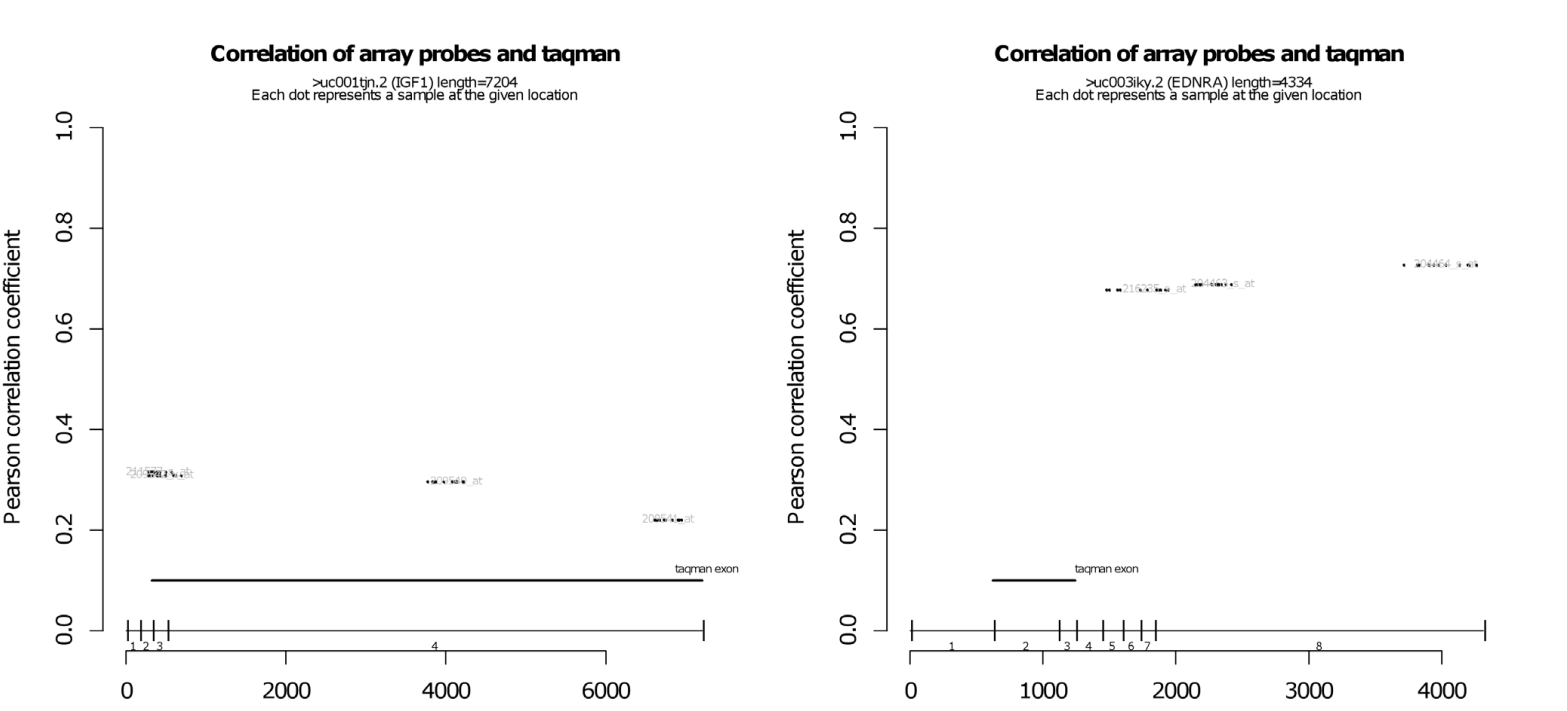
**Correlation of microarray and real-time PCR measurements, stratified by location of probe for EDNRA and IFG1**. The x-axis gives the position of a probe along the length of the gene. The y-axis gives the Pearson correlation between microarray probe set intensity and RPLP0-/TBP-normalized real-time PCR Ct value, both preprocessed as described in the methods section. Microarray probe sets are shown at a height corresponding to their correlation, with one dot for each probe in the set. Exact real-time PCR primer location is not available from Applied Biosystems, but the exon location of an assay is given. The locations of them are therefore marked with a thick horizontal line at a fixed height on the plot. The exon-intron architecture is indicated below the real-time PCR location. The value of the correlation is given in the first line below the plot. In some cases, the microarray probes were not found to match in the gene sequence used. They are likely to be probe sets for obsolete or alternative transcript isoforms. The correlation of these cases is indicated in the second line below the plot. The third line below the plot specifies how many real-time PCR double measurements had coefficients of variance above the threshold of 0.02. Additional file 4 contains this type of plot for all target genes. A figure similar to this, but using per-probe microarray data, is provided as Additional file 5.

Because of alternative splicing and the difference in probe location for the two measurements, biological variability can also explain differences in measurement techniques for some genes. It is therefore not necessarily sufficient to talk about measuring a particular gene with two methods; the measurements should also be performed on the same part of the gene. The locations of real-time PCR probes and primers and of microarray probe sets are shown for the examples of IGF1 and EDNRA in Figure 3. For all other genes, the same plots are available in Additional file 4. ENDRA shows a high degree of correlation, even though different sections of the gene are measured with the two techniques - the opposite is true for IGF1. For IGF1, alternative splicing is unlikely to be the reason behind the lack of correlation, but unfortunately, for almost all other genes (11 out of 15), the real-time PCR primers and microarray probe locations are not in the same exon. Without specific real-time PCR measurements of relevant regions, it is therefore difficult to make a decisive conclusion on the gene-wise differences in correlation between microarray and real-time PCR. Focus shall therefore be on the column-wise discussion, as follows.

### Differences in correlations between micorarray data and different real-time PCR normalizations

In Figure 2, the differences in correlations between different choices of endogenous controls can be read in each column. As demonstrated, the best correlation between microarray and real-time PCR is, on average, obtained when no endogenous control is used. The results obtained by using the geNorm method of Vandesompele et al. ranks 13^th ^(for the GAPDH/RPLP0/TBP combination) and 17^th ^(for GAPDH/RPLP0) among all 32 possible combinations of one or more of 5 endogenous control genes. This is an unexpected result, and it prompts a thorough discussion of the assumptions behind normalizations of PCR gene expression data.

A functional definition of gene expression should be expressed in units of mRNA per cell or nucleic mRNA concentration or a similar measure, because this is the level at which changes will affect the biology of the cell. In effect, the methods we compare here all rely on one of three assumptions to get to this functional definition: 1) that there exist one or more genes for which the expression is at a sufficiently constant level to be proportional to the amount of cells across all samples; this is the assumption behind the concept of endogenous control in ΔΔCt analysis; 2) that the overall distribution of the expression levels of all genes is the same in all cells, across all samples; this is the assumption behind the quantile normalization, which is the normalization part of the RMA pre-processing algorithm used for microarray analysis; and 3) that the total concentration of RNA is the same in all cells across all samples; this is the assumption behind the "no endogenous control" calculation of gene expression for real-time PCR data. This assumption stems from the fact that equal amounts of RNA were used in the sample preparation for the real-time PCR measurements, and it could as well be labeled "normalization to total RNA mass." It carries the further assumption that the reverse transcription of the RNA to cDNA does not introduce a bias.

## Conclusion

We here present gene expression measurements, obtained with different methods, on the same set of RNA samples. We analyze disagreements, which are of biological and technical origin: technical imprecision will surely obstruct data collection, but biological variation can also be thought to interfere through alternative splicing mechanisms. One finding is that introduction of housekeeping genes in the calculation perturbs the data without improving measurements. It has previously been speculated that normalizing to total RNA mass will produce better results in complex tissue [6], and these results support that notion.

The strength in our study is the high number of samples for which transcript analysis has been made with both real-time PCR and microarray. This in turns allow us to do the analysis on a detailed level. We see clear advantages with array analysis, because it has the total gene expression profile to normalize against. Therefore, the obtained data seem more robust normalization-wise, compared with real-time PCR. The advantages with real-time PCR are the sensitivity and usually a more optimal and up-to-date design of probe-primer pairs. The data, however, seem to be distorted while going through an endogenous control normalization procedure, either by a single housekeeping gene used out of tradition by the research team or by the geometric mean method, suggested by others [2]. While the bulk of the mass of total RNA is ribosomal RNA, our results show that it might nonetheless carry fewer perturbations to use this value as is.

This conclusion is of course limited by the circumstances in which we measure. One factor that is likely to be crucial is the highly heterogeneous tissue in question. The plaque-derived samples contain varying compositions of different cell types, and this can cause problems when assuming constant expression level of any gene across many different cell types of highly varying transcriptomic composition. The risk that cell composition changes in a way that affects the rRNA-to-mRNA ratio is of course also present, but the data show that at least in this data set, this not the case.

Another factor that could be of interest to the conclusion is the methods with which the cDNA for real-time PCR is prepared. The RNA quality of all samples is carefully controlled, and degraded samples are excluded. Furthermore, the quantification system in use could possibly provide for better precision than previously used spectrophotometers.

## Methods

### Ethics statement

Biobank materials were extracted after informed written consent from all participants were obtained according to the Declaration of Helsinki and approved by the ethical committee of the Karolinska Institute, journal number 02-147.

### Sample preparation

All samples were part of the Biobank of the Karolinska Carotid Endarterectomies (BiKE) cohort [7], which consists of samples from 400 patients undergoing carotid endarterectomy. Patients were 70.0 ± 8.56 years, 28.9% being females. Ten of the samples included in the analysis were taken from healthy control tissue (iliaca and aorta). Tissue samples were cryohomogenized using a Mikro Dismembrator S (B Braun Biotech International GmbH, Melsungen, Germany), and cells were lysed with RLT buffer (Qiagen, Valencia, CA, USA). Total RNA was isolated using the RNeasy extraction kit (Qiagen). The optional on-column DNase digestion step was included. RNA quality was assessed with a Bioanalyzer capillary electrophoresis system (Agilent Technology), and degraded samples were excluded from further analysis. RNA concentration was measured using a Nanodrop 1000 spectrophotometer (Thermo Scientific).

### Microarray measurements

Microarray hybridization and scanning were done at the Karolinska Institute core facility for Bioinformatics and Expression Analysis, using Affymetrix HG-U133 plus 2.0 type microarrays. One hundred seventeen of the samples have been scanned on HG-U133 plus 2.0 microarrays. All cel files were visually inspected for scratches and subjected to the NUSE and RPE quality control algorithms available from the affyPLM Bioconductor package. Cel files were analyzed using Robust Multichip Average (RMA), as implemented in the Affymetrix Power Tools 1.8.6 package apt-probeset-summarize. Because the normalization is of specific interest here, it will briefly be described. RMA, as introduced by Irizarry et al. [8], includes a normalization step, a background adjustment step, and a summarization step. The normalization step, known as quantile normalization, was introduced separately and consists of an algorithm for normalizing a set of data vectors by giving them the same distribution [9]. The normalized data are background-adjusted, and probe values are summarized per probe set using a median polish function. Notably, the data are log2-transformed in this process, which is done to obtain a closer relation to mRNA concentrations as seen in spike-in experiments and to provide more normal distributions for subsequent statistical analysis. The data in Figures 2 and 3 are RMA-summarized. To check that background correction and summarization are not obscuring results, an alternative analysis using probe level data that have only been quantile-summarized is available in Additional file 5. Furthermore, the entire analysis was repeated with the same findings using three different microarray pre-processing methods - the gcRMA, as implemented in the Bioconductor package of the same name; the PLIER-MM, as implemented in Affymetrix Power Tools; and the model-based expression with invariant set normalization, as implemented in dChip 2008 software [10]. Probe level and probe set level microarray data have been provided as Additional file 6

### Real-time PCR measurement

Total RNA from 157 samples was reverse-transcribed using SuperScript II reverse transcriptase (Invitrogen) and random hexamers, using mastermix for all included samples in one batch. The same amount of total RNA was used for each sample. The quantification was based on Nanodrop 1000 spectrophotometer (Thermo Scientific) measurements. TaqMan probes and primers for all genes mentioned were purchased as assay-on-demand (Applied Biosystems). For each gene, PCR amplification was done in four 96-well plates in a either a 7700 or a 7900 HT fast real-time PCR system (Applied Biosystems). All samples were measured in double. We defined a reasonable disagreement between replicates as a coefficient of variance of 0.02, because this was the value all measurements met on the best plates. All endogenous controls except PPIA had replicate values that were all below this threshold. PPIA had 6 samples with a coefficient of variance above the threshold. The summarized precision of other gene measurements is specified in the bottom line of Additional file 4 and fully described in Additional file 3. All 96-well plates included a commercial RNA standard to control for systematic interplate variability. All real-time PCR analyses were done using the ΔΔCt method with the mean of all samples as calibrator and a specified set of endogenous controls [1]. In the case of more than one endogenous control, the geometric mean was used, as specified [2]. In the case of no endogenous control, the first normalization step was omitted, and a calibrator value of the mean of all Ct values was subtracted from raw Ct values before calculating the power with two as base. Exact calculations can be found in the included R-script in Additional file 7.

### Selection of target genes and endogenous control genes

Endogenous control genes PPIA and B2 M were selected arbitrarily for initial analysis. The remaining candidates were selected based on 1) low coefficient of variance in microarray data of a subset of the samples; 2) as low a correlation as possible to clinical parameters that could be of interest (symptom, diabetes, statins, time between symptom and operation, HbA1c values, serum cholesterol); and 3) mean expression level above some fraction of the mean of all probe sets. Target genes were all measured as part of nonrelated projects on the BiKE dataset and are therefore in this respect arbitrarily selected. The target genes are not part of any common functional or cell type-specific groups.

### Exon location of microarray probe sets

To retrieve the exact location of each probe set, we performed sequence matching of probe sequences with sequences of the gene of interest. It was matched to the sequence of the gene in question to give the position using tools available in the GeneRegionScan package [11,12], available from the Bioconductor repository [13]. Gene sequences were downloaded from the UCSC genome browser [14] human Mar. 2006 assembly. In cases with more than one known isoform, preference was given to the longest variant. UCSC ID-number for each sequence is given in the top of each plot.

### Sensitivity analysis

A sensitivity analysis, similar to the one performed by Qin et al. [5], showed that systematic removal of single genes did not affect final conclusions. No single gene changes the fact that the omission of endogenous control produces the best correlation.

## Authors' contributions

LF carried out the bioinformatical analysis and drafted the manuscript. SK provided statistical feedback. HA helped obtain the samples and measurements. AR, AG, and GB conceived of the study and participated in its design and coordination. All authors read and approved the final manuscript.

## Supplementary Material

Additional file 1 **scatter plots of real-time PCR and array data for all genes of interest**. The zip-file contains pdf files for each gene of interest. Each row in the pdf file shows a probe set investigating the gene in question. Each column in the pdf file shows different combinations of endogenous controls used for preparing the real-time PCR data. Click here for file

Additional file 2 **investigations of systematic patterns of relation between SD, mean and correlation metric**. For the investigation of systematic bias of correlation metric in relation to standard deviation and absolute expression. Click here for file

Additional file 3 **raw Ct values for all samples measured with real-time PCR**. It contains a sheet for each gene. The columns named Value 1 and Value 2 contain raw Ct values of the data. Click here for file

Additional file 4 **Figure **3**for all target genes**. The figure text of Figure 3 applies here as well. Click here for file

Additional file 5 **Figure **3, **without probe set summarization**. The figure text of Figure 3 applies here as well, with the only change being that microarray data were not RMA-normalized. Instead, the quantile-normalized probe level values were extracted using the GeneRegionScan package. Full details can be found in the Additional file 7 script. Click here for file

Additional file 6 **raw microarray data for target genes**. Probe level data has been generated with a ReadAffy call on the cell files, followed by an exprs call for the genes of interest on the generated AffyBatch. Probe set level data has been generated using the RMA algorithm as implemented in Affymetrix Power Tools. Click here for file

Additional file 7 **R/Bioconductor script for obtaining all plots**. The script needs a few prerequisites, such as array data and gene sequences, but given them it should produce the exact same results as shown here. The content is plain text format, viewable with any text editor and runnable in R 2.8.0 [15]. Click here for file
