# Supplementary material for: Endogenous control genes in complex vascular tissue samples
Source: BMC Genomics. 2009 Nov 10;10:516. doi: 10.1186/1471-2164-10-516 (PMC2779820; doi:10.1186/1471-2164-10-516)
Supplement: Additional file 2 — investigations of systematic patterns of relation between SD, mean and correlation metric. For the investigation of systematic bias of correlation metric in relation to standard deviation and absolute expression. [file 1471-2164-10-516-S2.pdf]

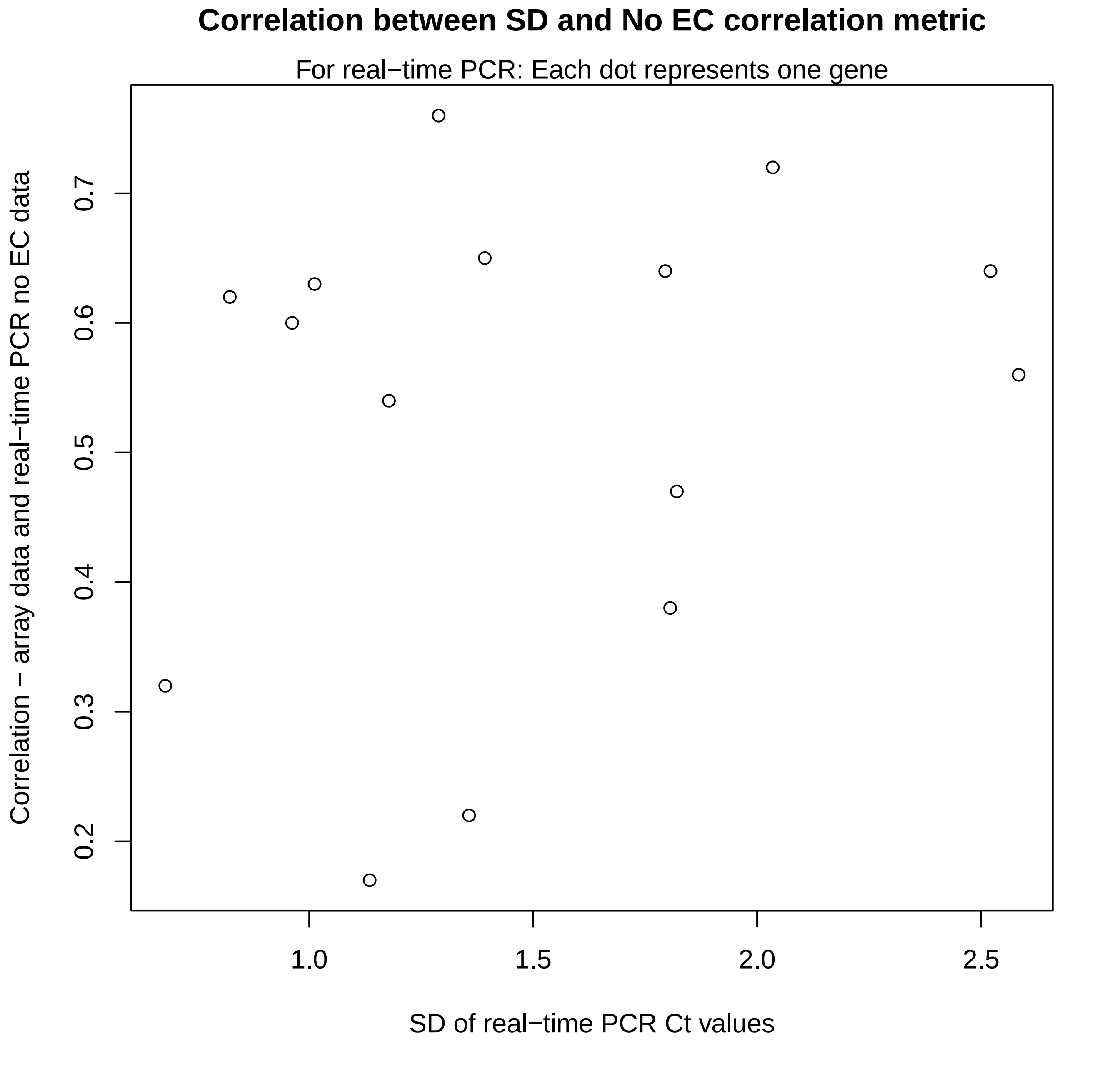
Correlation between SD and No EC correlation metric
For real−time PCR: Each dot represents one gene
Correlation − array data and real−time PCR no EC data
0.2
0.3
0.4
0.5
0.6
0.7
1.0
1.5
2.0
2.5
SD of real−time PCR Ct values

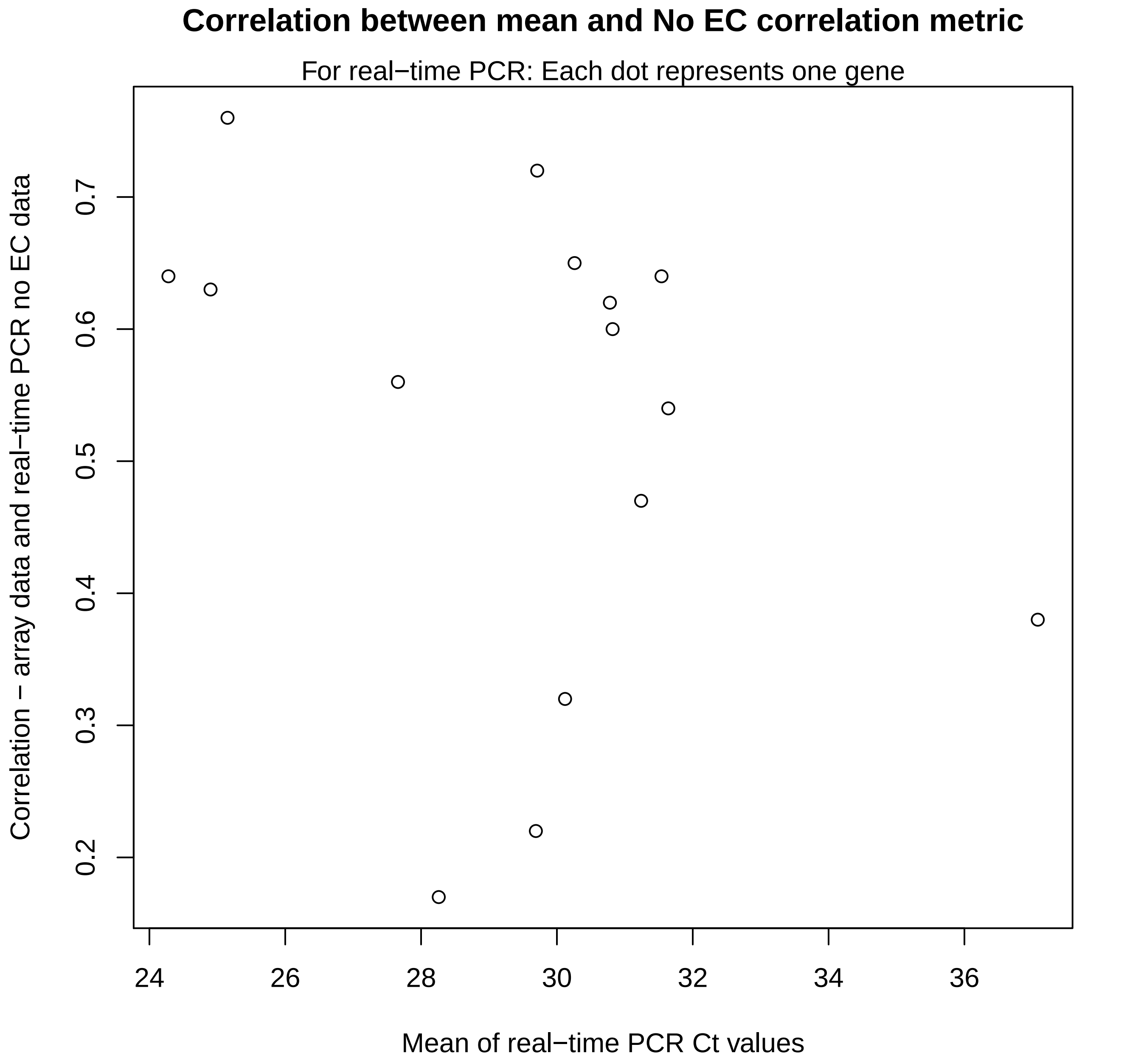
Correlation between mean and No EC correlation metric
For real−time PCR: Each dot represents one gene
Correlation − array data and real−time PCR no EC data
0.2
0.3
0.4
0.5
0.6
0.7
24
26
28
30
32
34
36
Mean of real−time PCR Ct values

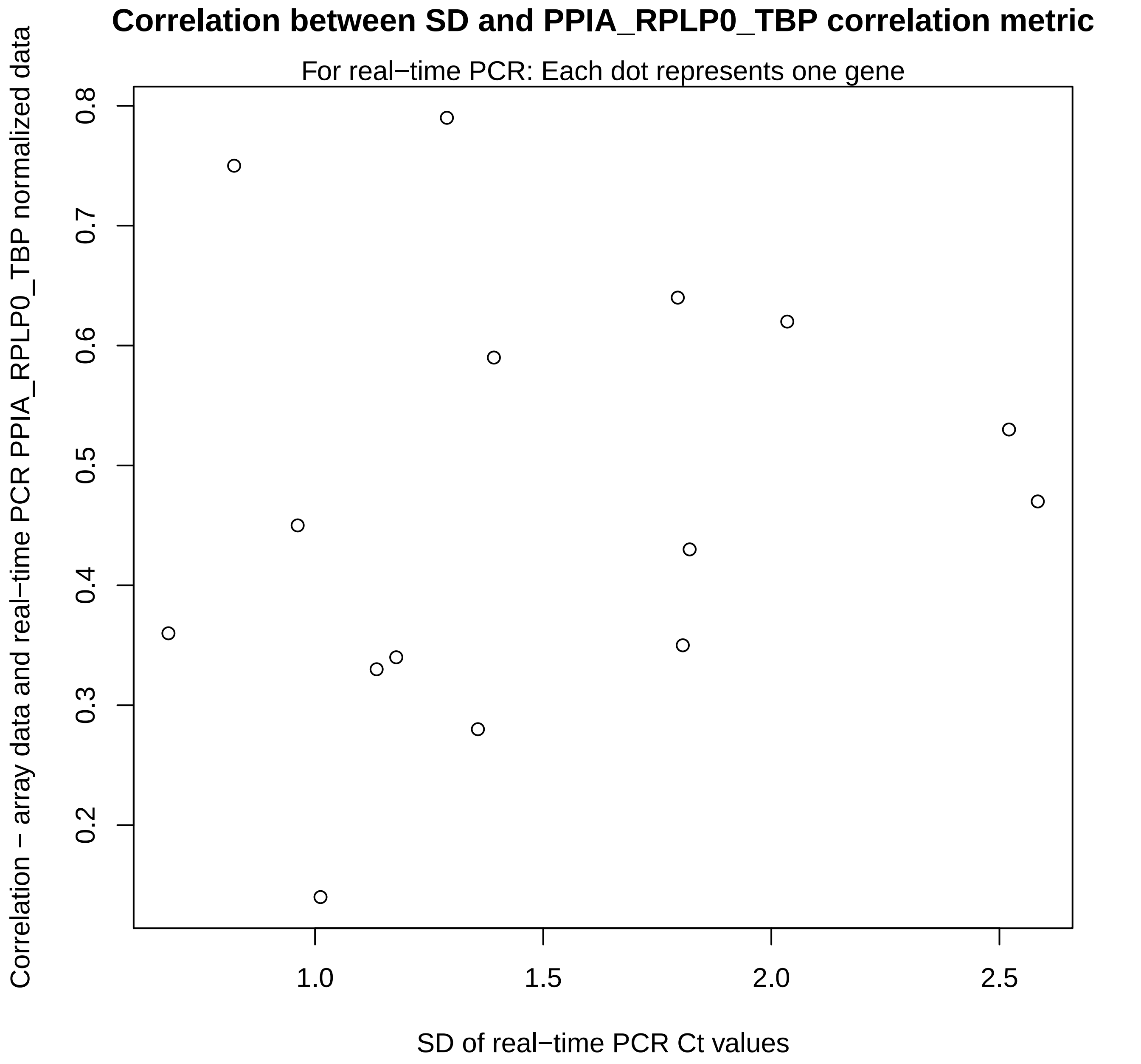
Correlation between SD and PPIA_RPLP0_TBP correlation metric
For real−time PCR: Each dot represents one gene
Correlation − array data and real−time PCR PPIA_RPLP0_TBP normalized data
0.2
0.3
0.4
0.5
0.6
0.7
0.8
1.0
1.5
2.0
2.5
SD of real−time PCR Ct values

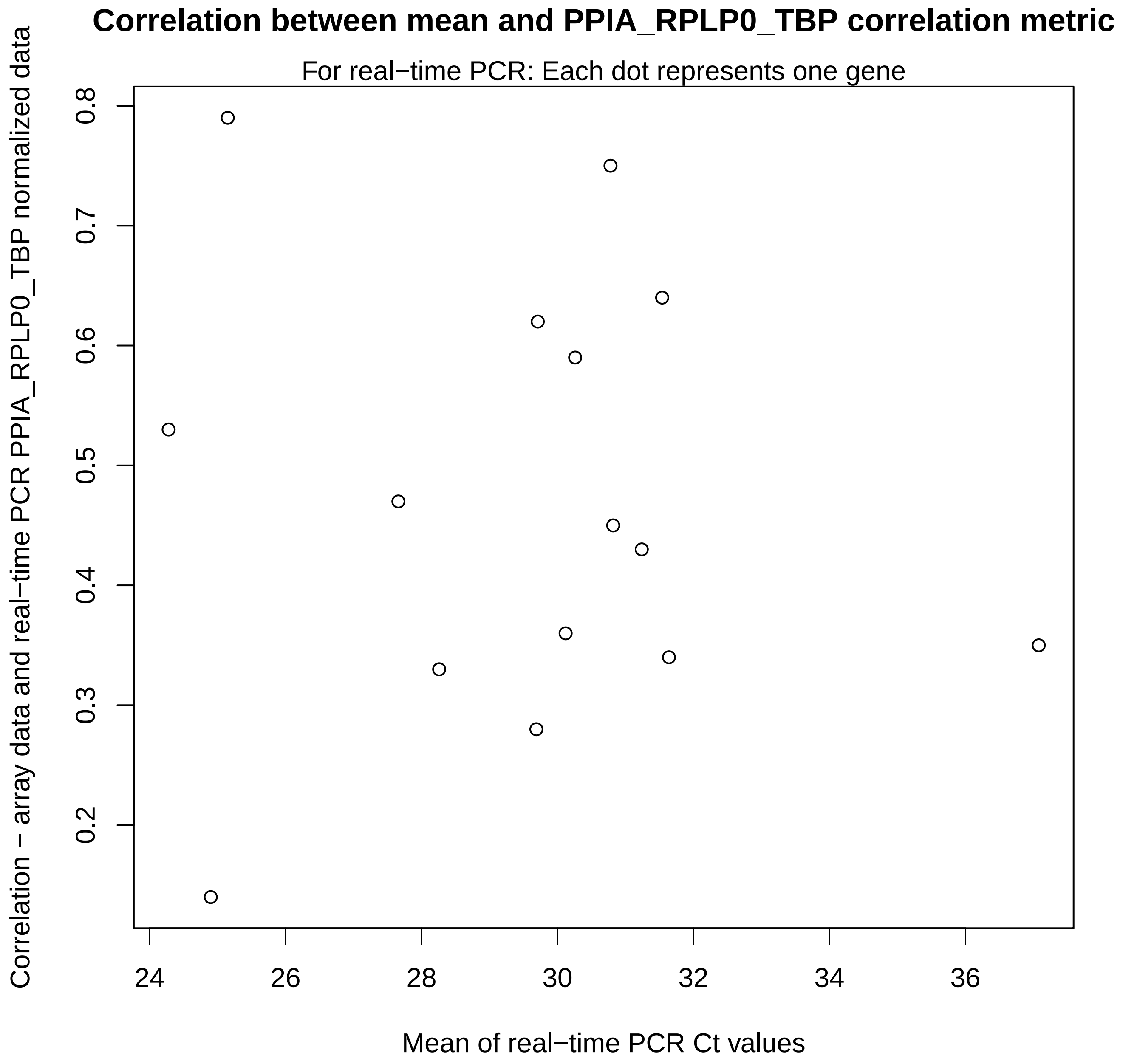
Correlation between mean and PPIA_RPLP0_TBP correlation metric
For real−time PCR: Each dot represents one gene
Correlation − array data and real−time PCR PPIA_RPLP0_TBP normalized data
0.2
0.3
0.4
0.5
0.6
0.7
0.8
24
26
28
30
32
34
36
Mean of real−time PCR Ct values

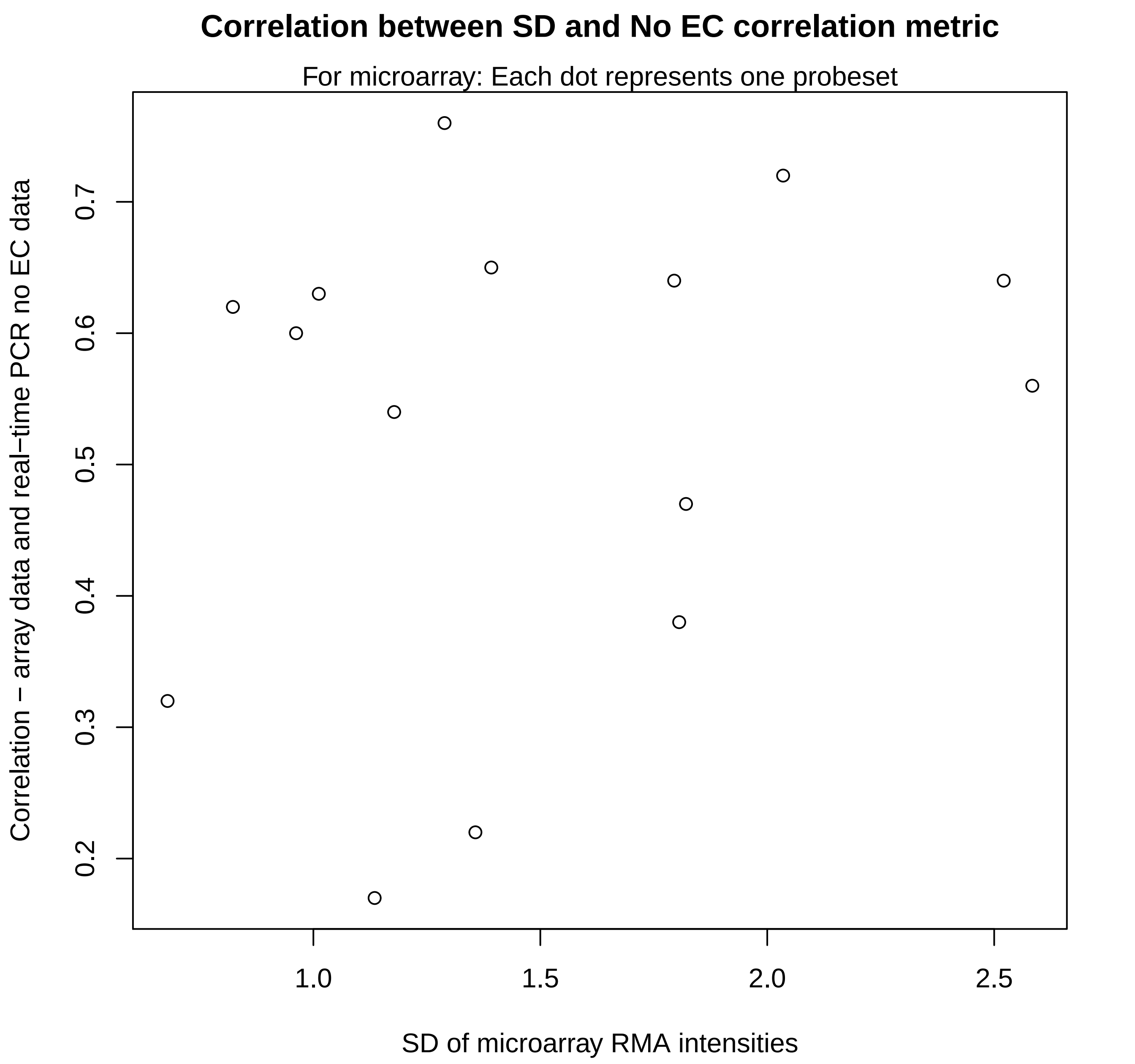

Correlation between SD and No EC correlation metric
For microarray: Each dot represents one probeset
Correlation − array data and real−time PCR no EC data
0.2
0.3
0.4
0.5
0.6
0.7
1.0
1.5
2.0
2.5
SD of microarray RMA intensities

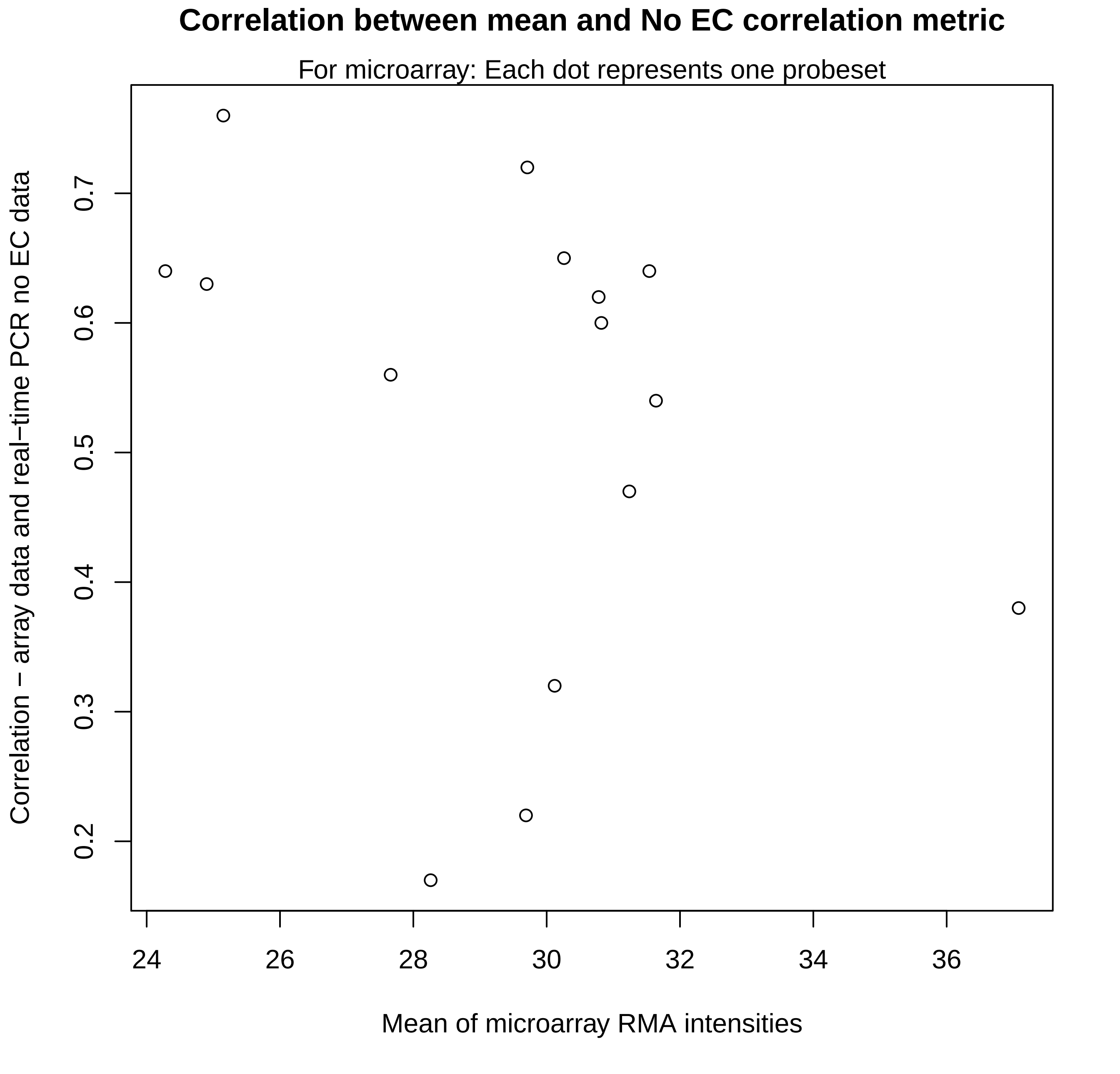
Correlation between mean and No EC correlation metric
For microarray: Each dot represents one probeset
Correlation − array data and real−time PCR no EC data
0.2
0.3
0.4
0.5
0.6
0.7
24
26
28
30
32
34
36
Mean of microarray RMA intensities

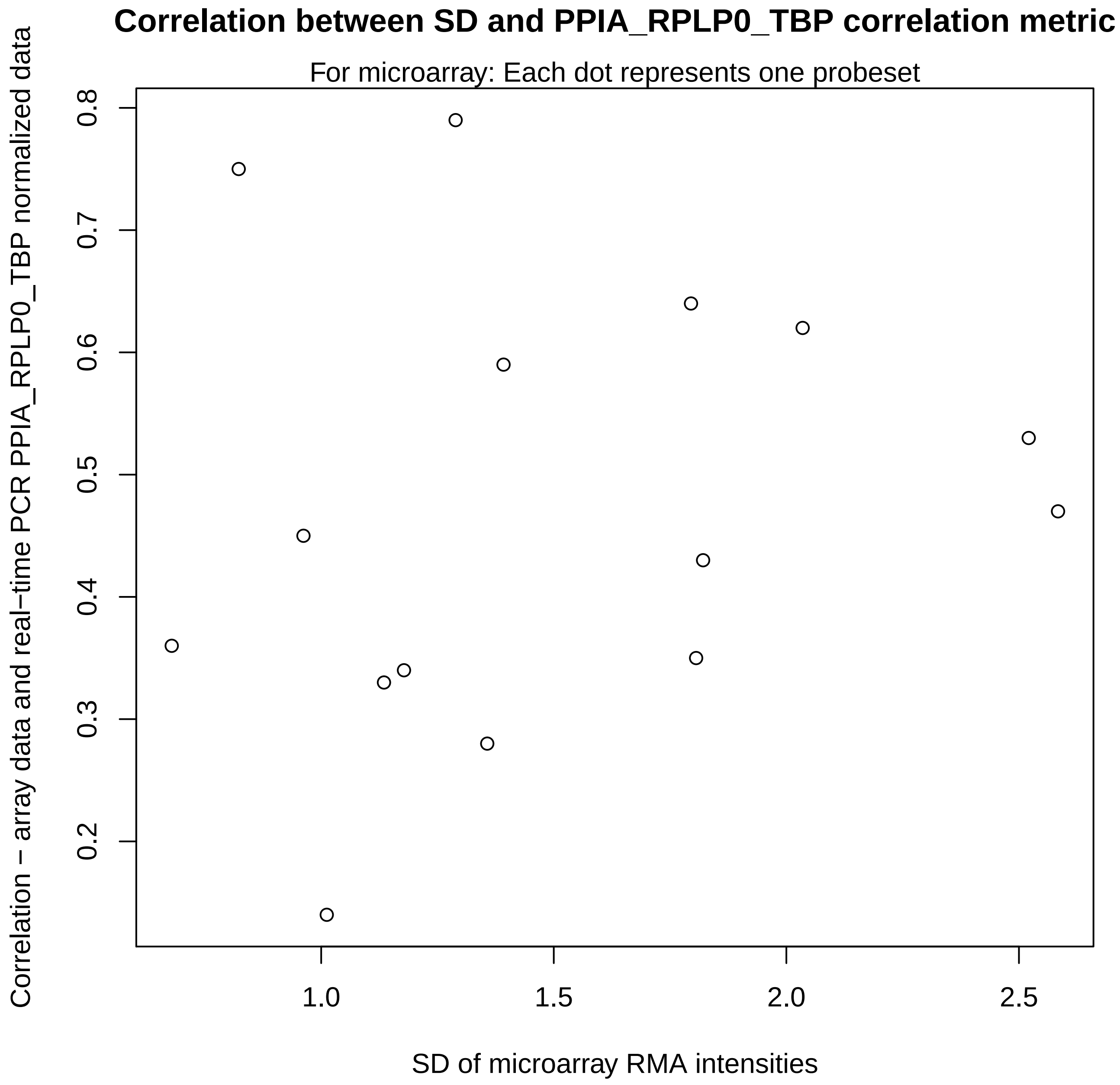
Correlation between SD and PPIA_RPLP0_TBP correlation metric
For microarray: Each dot represents one probeset
Correlation − array data and real−time PCR PPIA_RPLP0_TBP normalized data
0.2
0.3
0.4
0.5
0.6
0.7
0.8
1.0
1.5
2.0
2.5
SD of microarray RMA intensities

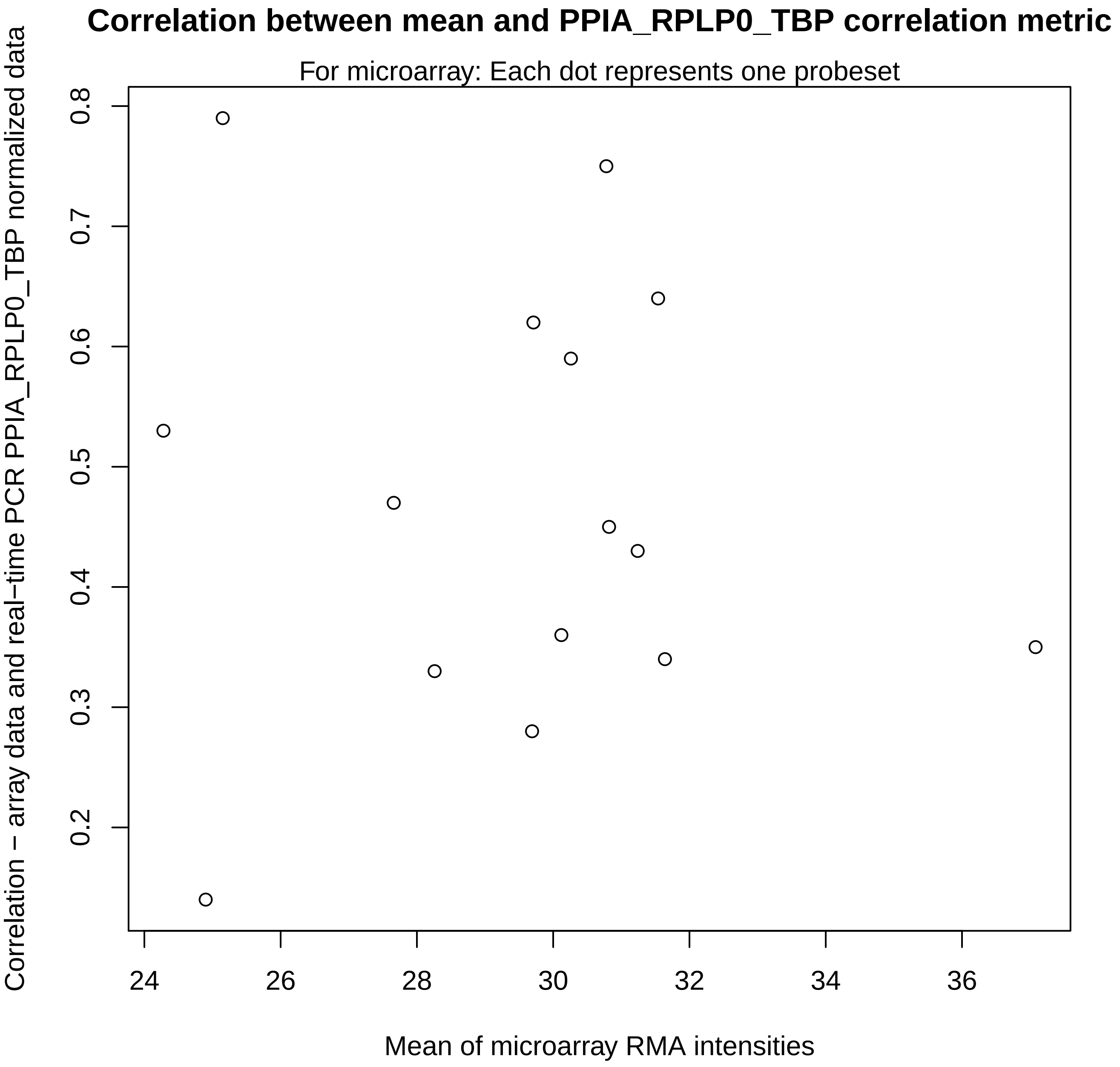

Correlation between mean and PPIA_RPLP0_TBP correlation metric
For microarray: Each dot represents one probeset
Correlation – array data and real−time PCR PPIA_RPLP0_TBP normalized data
0.2
0.3
0.4
0.5
0.6
0.7
0.8
24
26
28
30
32
34
36
Mean of microarray RMA intensities
