# Supplementary material for: Endogenous control genes in complex vascular tissue samples
Source: BMC Genomics. 2009 Nov 10;10:516. doi: 10.1186/1471-2164-10-516 (PMC2779820; doi:10.1186/1471-2164-10-516)
Supplement: Additional file 4 — Figure 3for all target genes. The figure text of Figure 3 applies here as well. [file 1471-2164-10-516-S4.pdf]

Correlation of array probes and taqman

>uc003iky.2 (EDNRA) length=4334
Each dot represents a sample at the given location

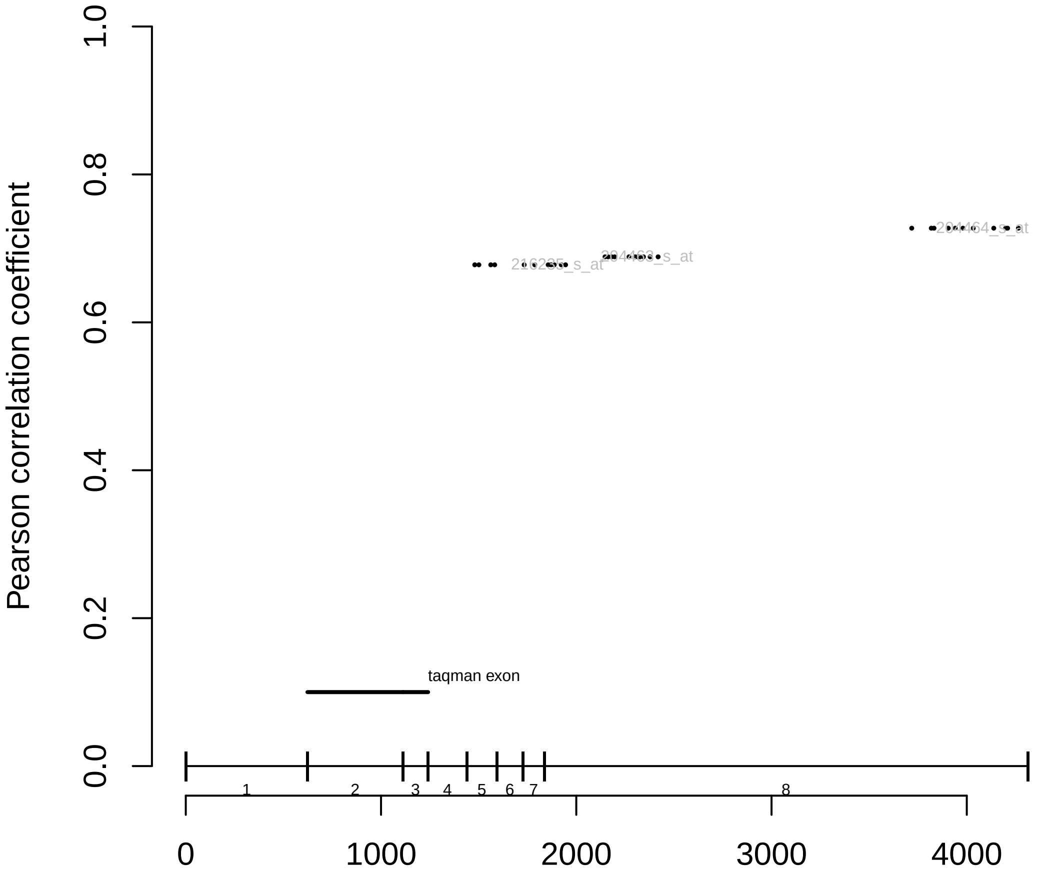

Found 3 probesets with correlation coefficient(s): 204463_s_at: 0.689 204464_s_at: 0.727 216235_s_at: 0.678
Found all probesets
Number of taqman replicate measurements with CV above threshold: 2

Correlation of array probes and taqman

>uc003vvh.1 (TBXAS1) length=2246
Each dot represents a sample at the given location

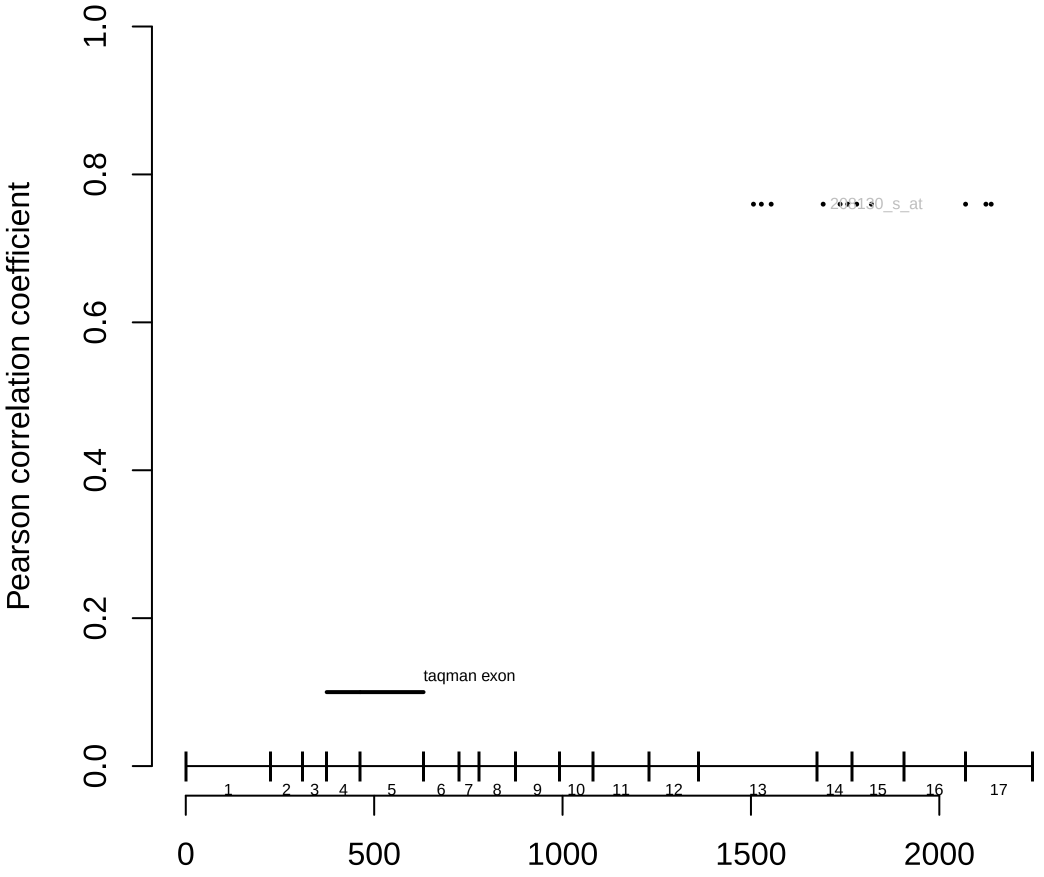

Found 1 probesets with correlation coefficient(s): 208130_s_at: 0.76

1 probesets did not match in the given mRNA. They had correlation coefficient(s): 236345_at: 0.565

Number of taqman replicate measurements with CV above threshold: 8

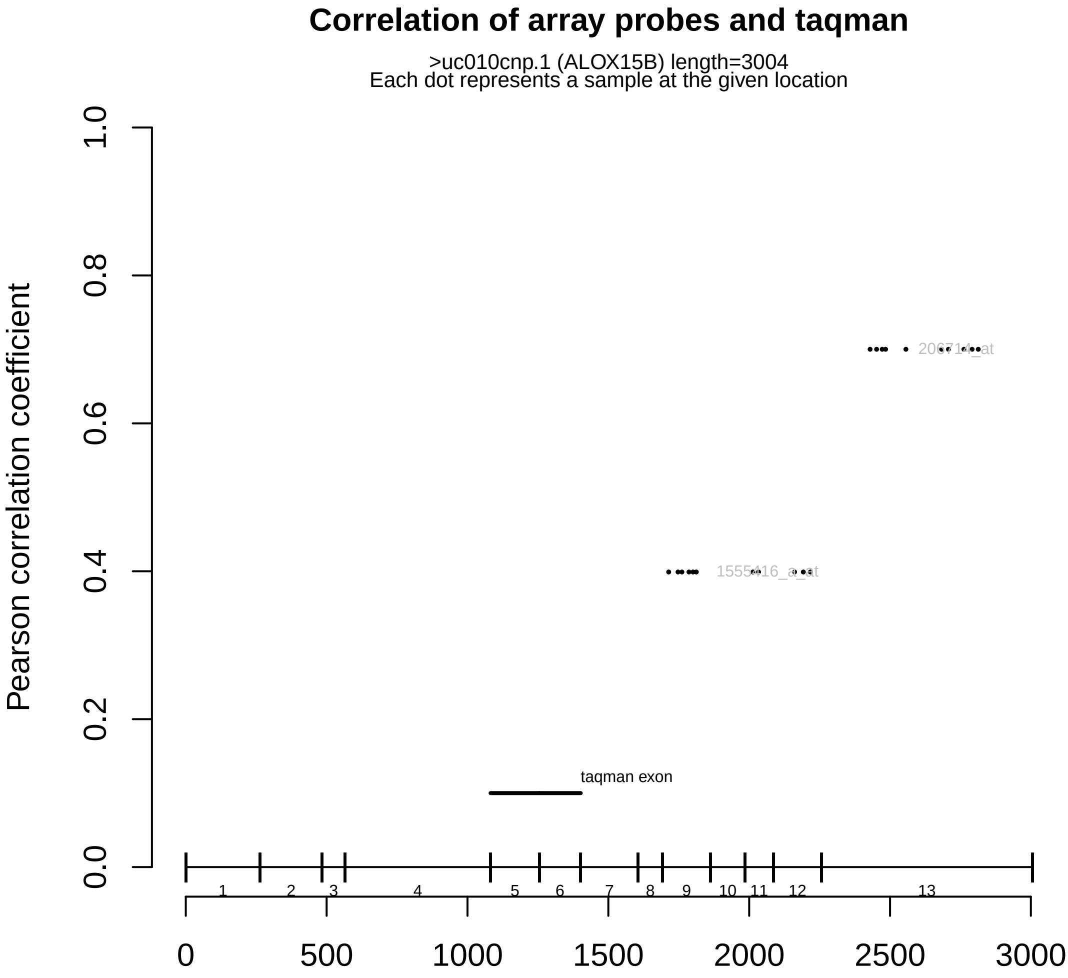

Found 2 probesets with correlation coefficient(s): 1555416_a_at: 0.399   206714_at: 0.7

Found all probesets

Number of taqman replicate measurements with CV above threshold: 10

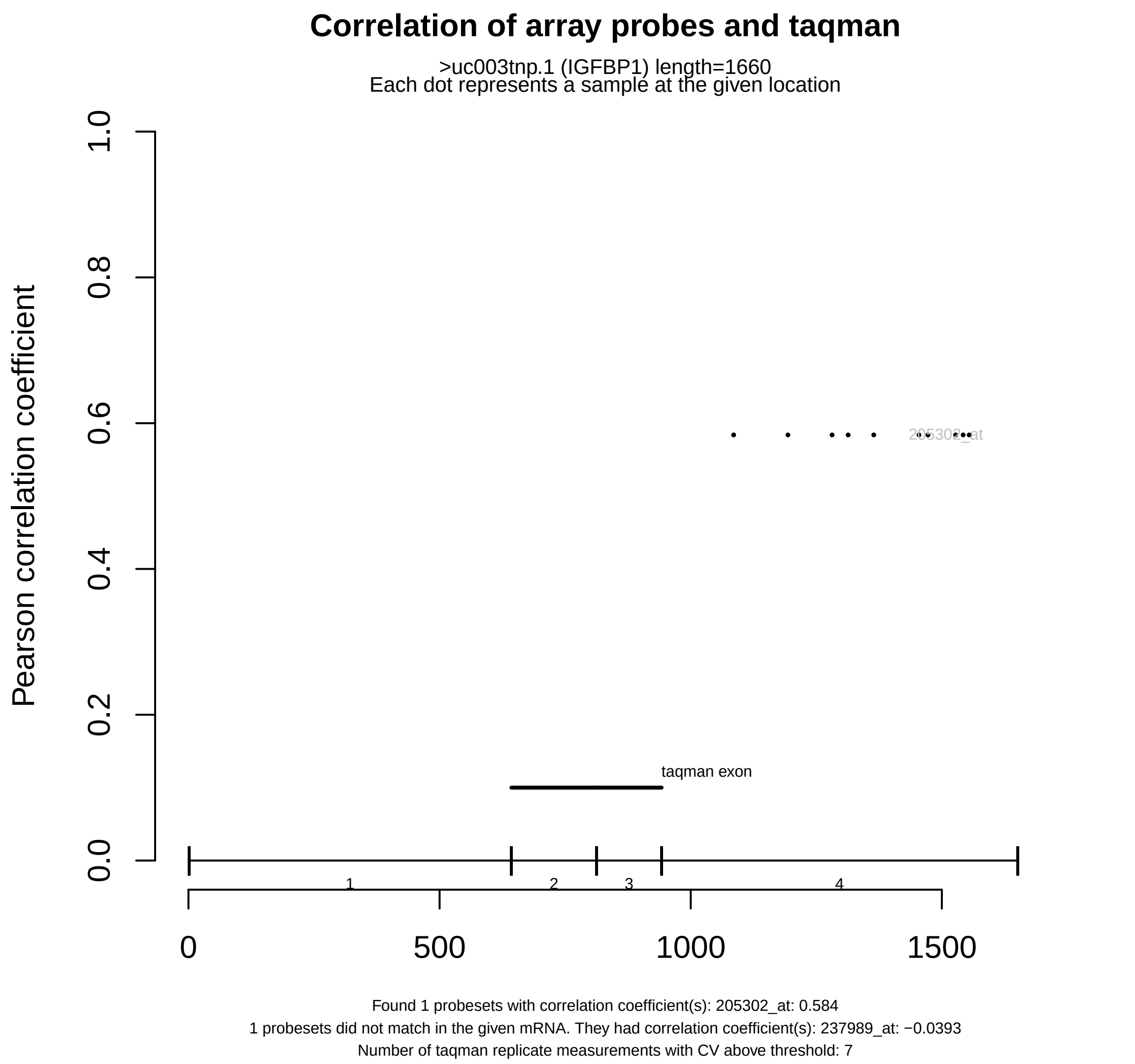
Correlation of array probes and taqman
>uc003tnp.1 (IGFBP1) length=1660
Each dot represents a sample at the given location
Pearson correlation coefficient
0.0
0.2
0.4
0.6
0.8
1.0
205302_at
taqman exon
1
2
3
4
0
500
1000
1500
Found 1 probesets with correlation coefficient(s): 205302_at: 0.584
1 probesets did not match in the given mRNA. They had correlation coefficient(s): 237989_at: −0.0393
Number of taqman replicate measurements with CV above threshold: 7

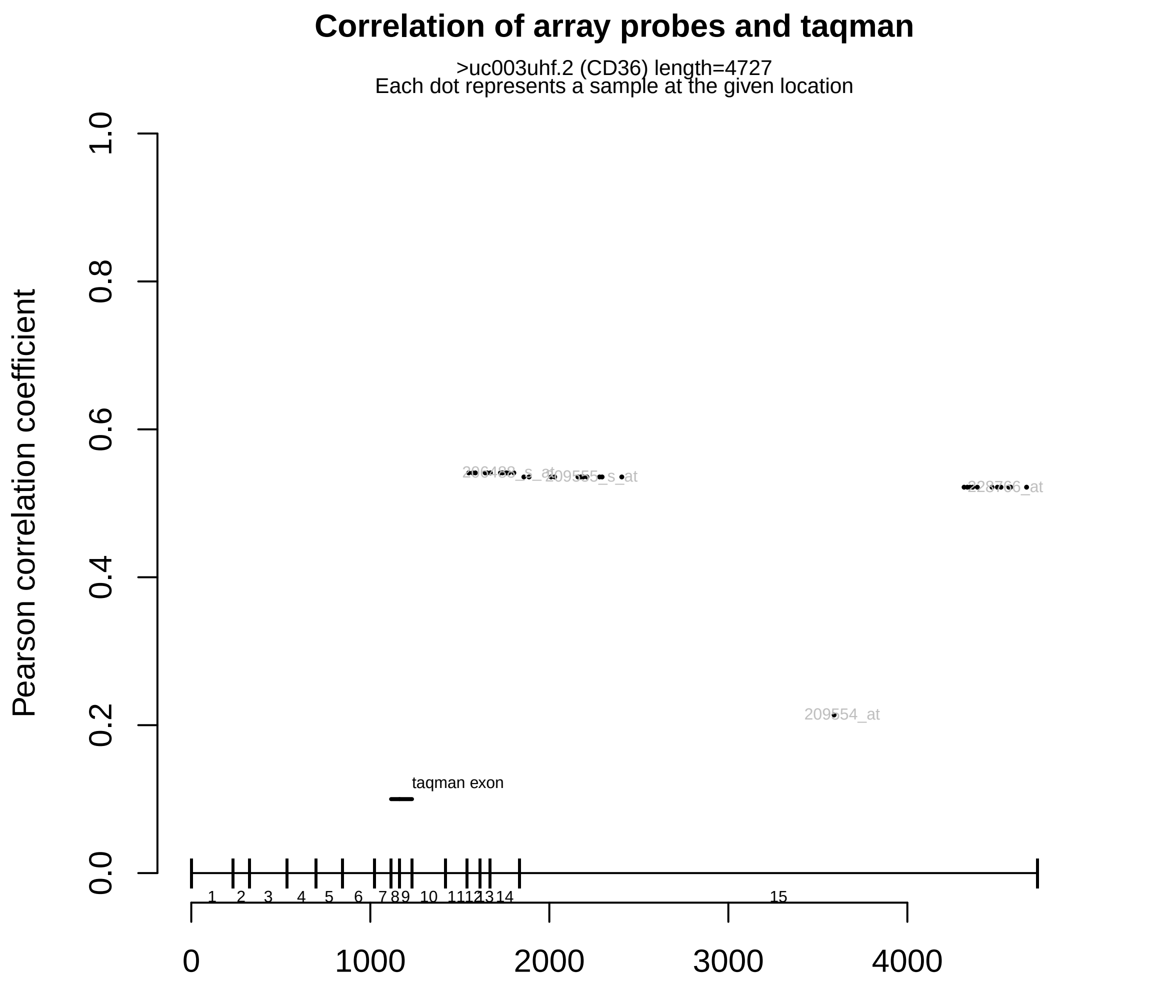

Found 4 probesets with correlation coefficient(s): 206488_s_at: 0.541 209554_at: 0.214 209555_s_at: 0.536 228766_at: 0.522
1 probesets did not match in the given mRNA. They had correlation coefficient(s): 242197_x_at: 0.617
Number of taqman replicate measurements with CV above threshold: 10

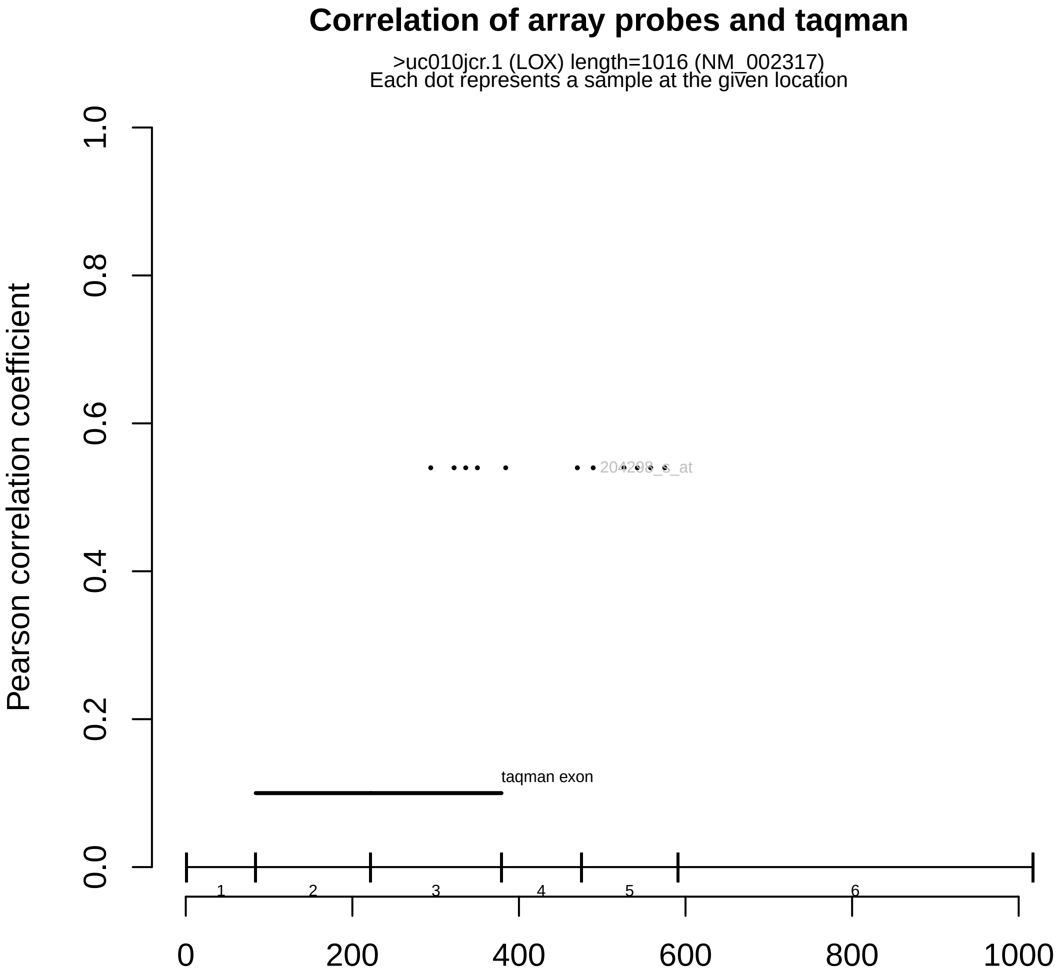

Found 1 probesets with correlation coefficient(s): 204298_s_at: 0.54

2 probesets did not match in the given mRNA. They had correlation coefficient(s): 213640_s_at: 0.369   215446_s_at: 0.641

Number of taqman replicate measurements with CV above threshold: 0

Correlation of array probes and taqman

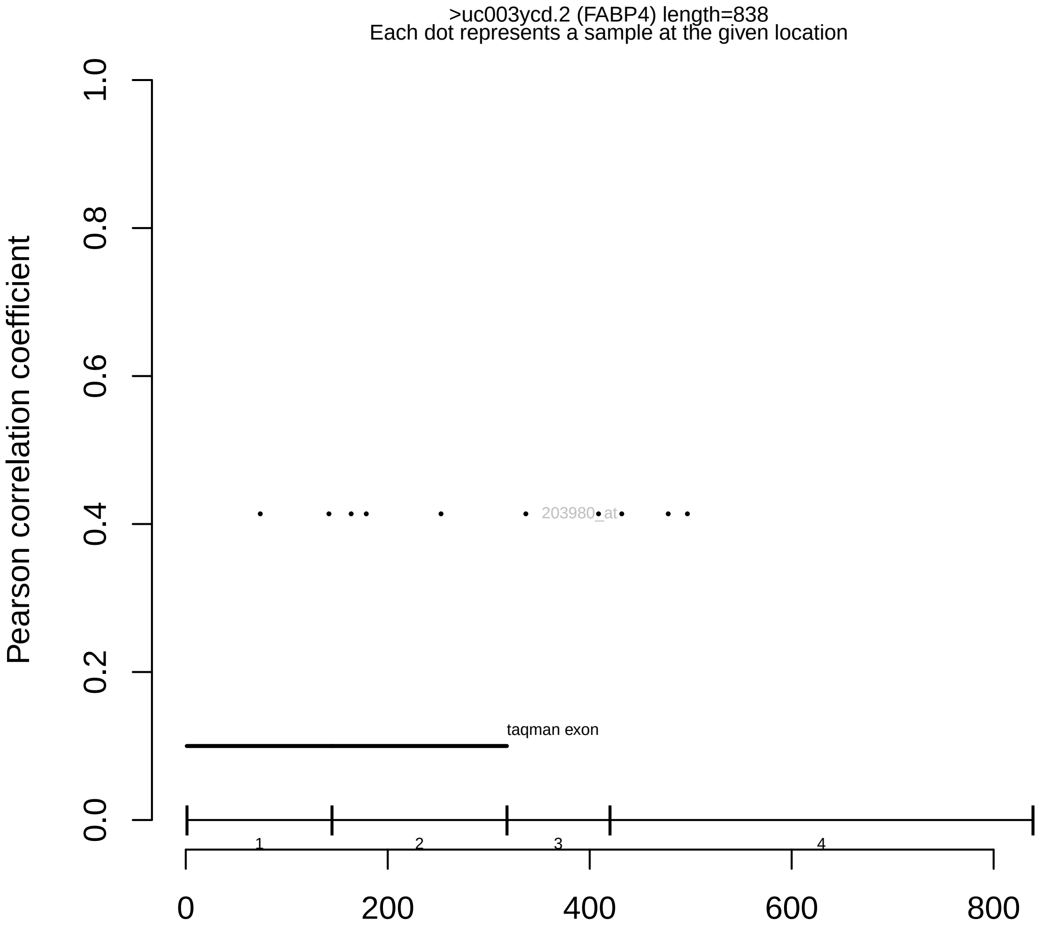

Found 1 probesets with correlation coefficient(s): 203980_at: 0.414

1 probesets did not match in the given mRNA. They had correlation coefficient(s): 235978_at: 0.659

Number of taqman replicate measurements with CV above threshold: 11

Correlation of array probes and taqman

>uc002mfh.1 (TNFSF9 / CD137L) length=1645 (from NM_003811, inclusive 3 x G in the middle but excl poly−A tail
Each dot represents a sample at the given location

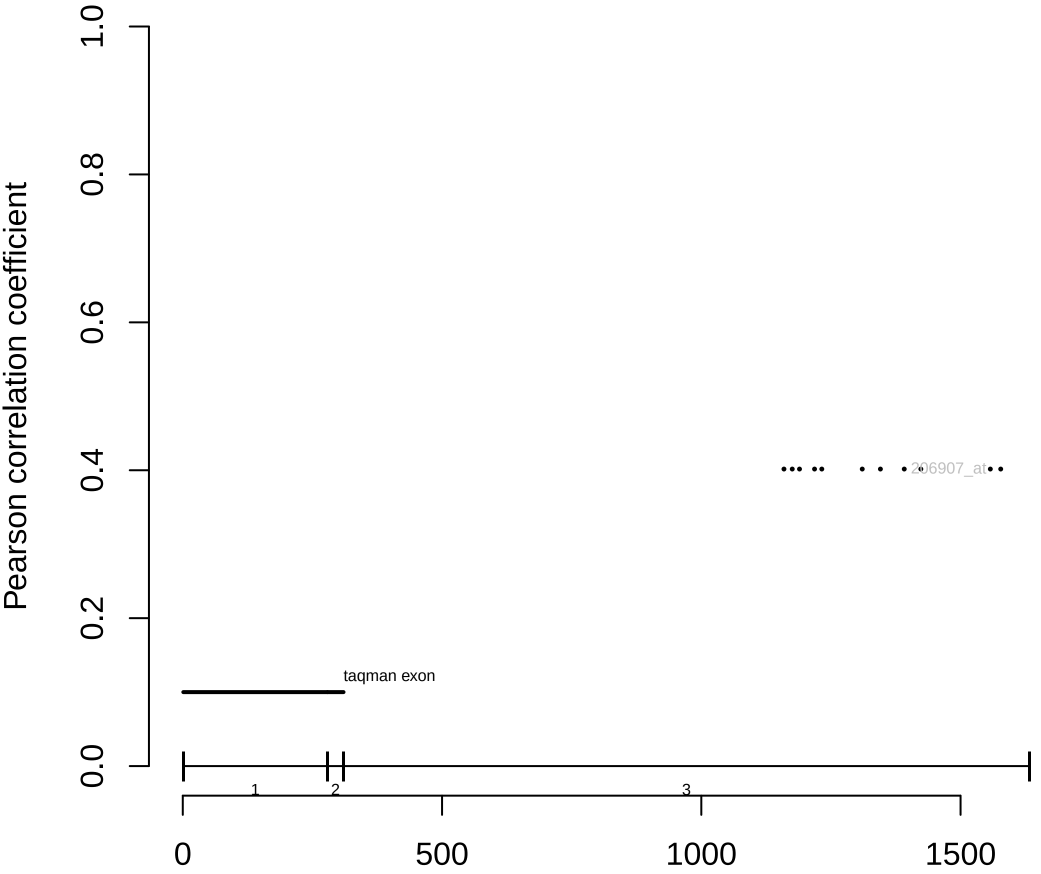

Found 1 probesets with correlation coefficient(s): 206907_at: 0.402

Found all probesets

Number of taqman replicate measurements with CV above threshold: 5

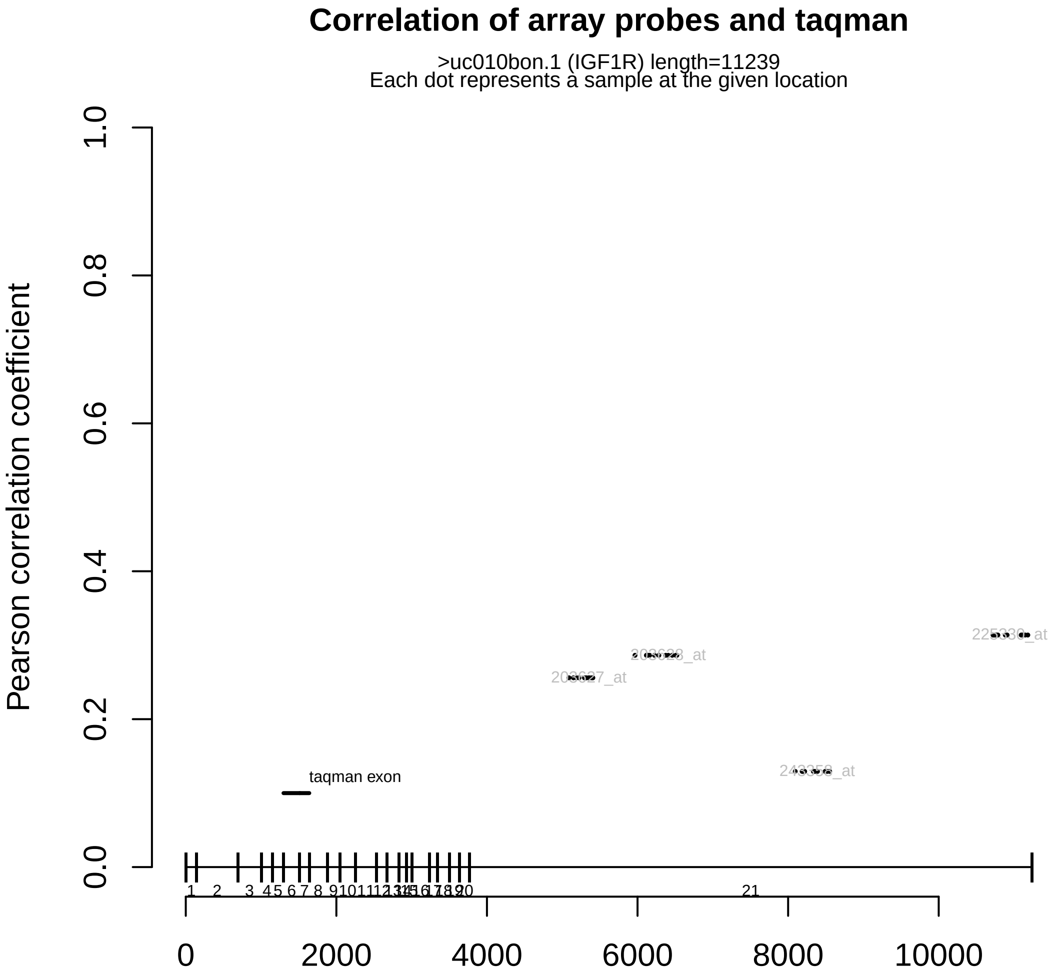

Found 4 probesets with correlation coefficient(s): 203627_at: 0.256   203628_at: 0.286   225330_at: 0.314   243358_at: 0.129

1 probesets did not match in the given mRNA. They had correlation coefficient(s): 208441_at: −0.00669

Number of taqman replicate measurements with CV above threshold: 3

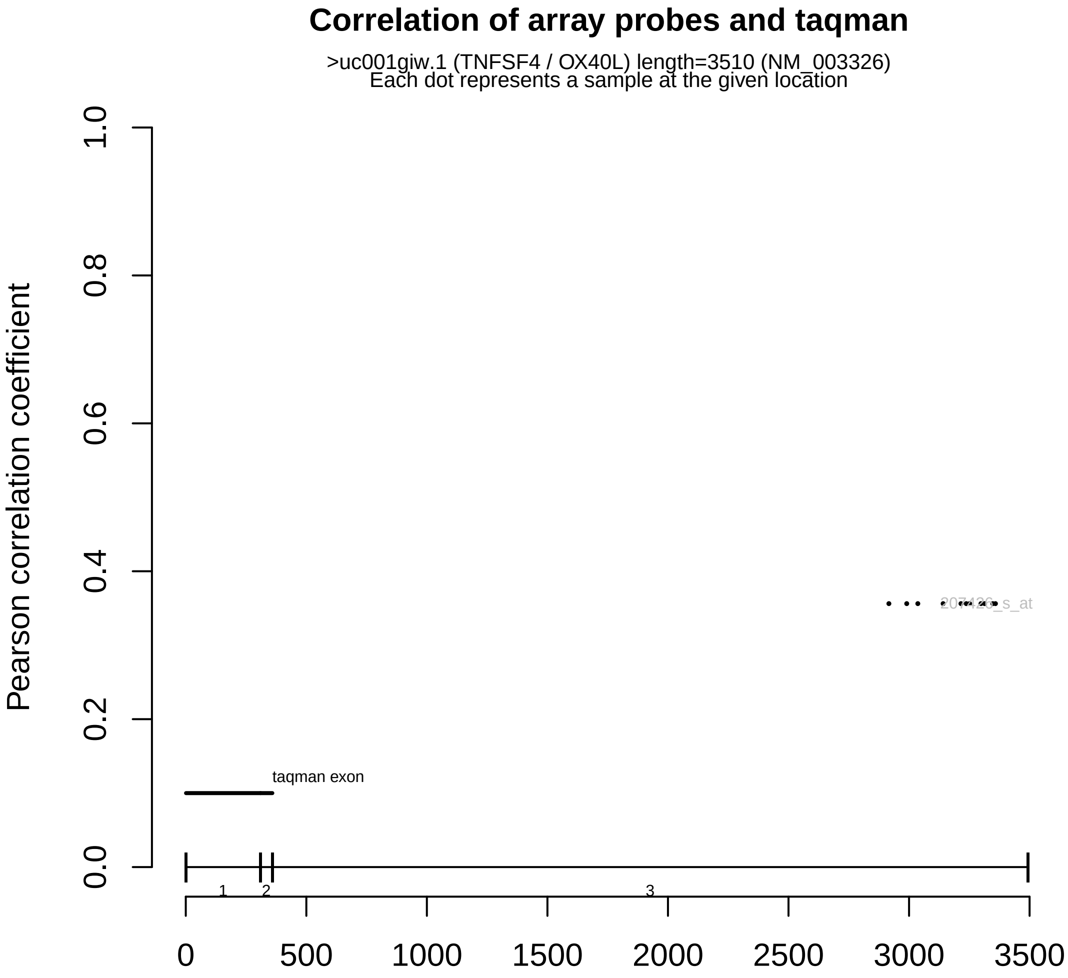

Found 1 probesets with correlation coefficient(s): 207426_s_at: 0.356

Found all probesets

Number of taqman replicate measurements with CV above threshold: 10

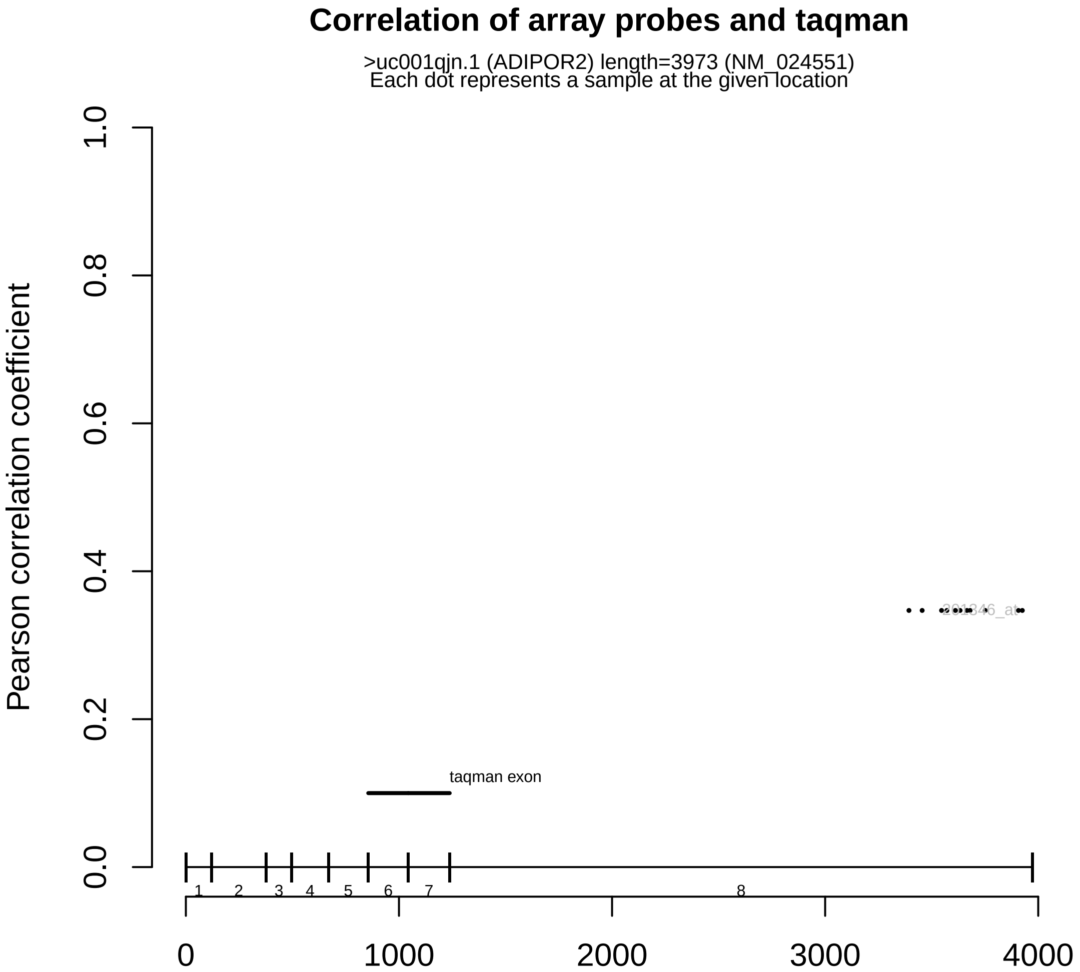

Found 1 probesets with correlation coefficient(s): 201346_at: 0.347

Found all probesets

Number of taqman replicate measurements with CV above threshold: 4

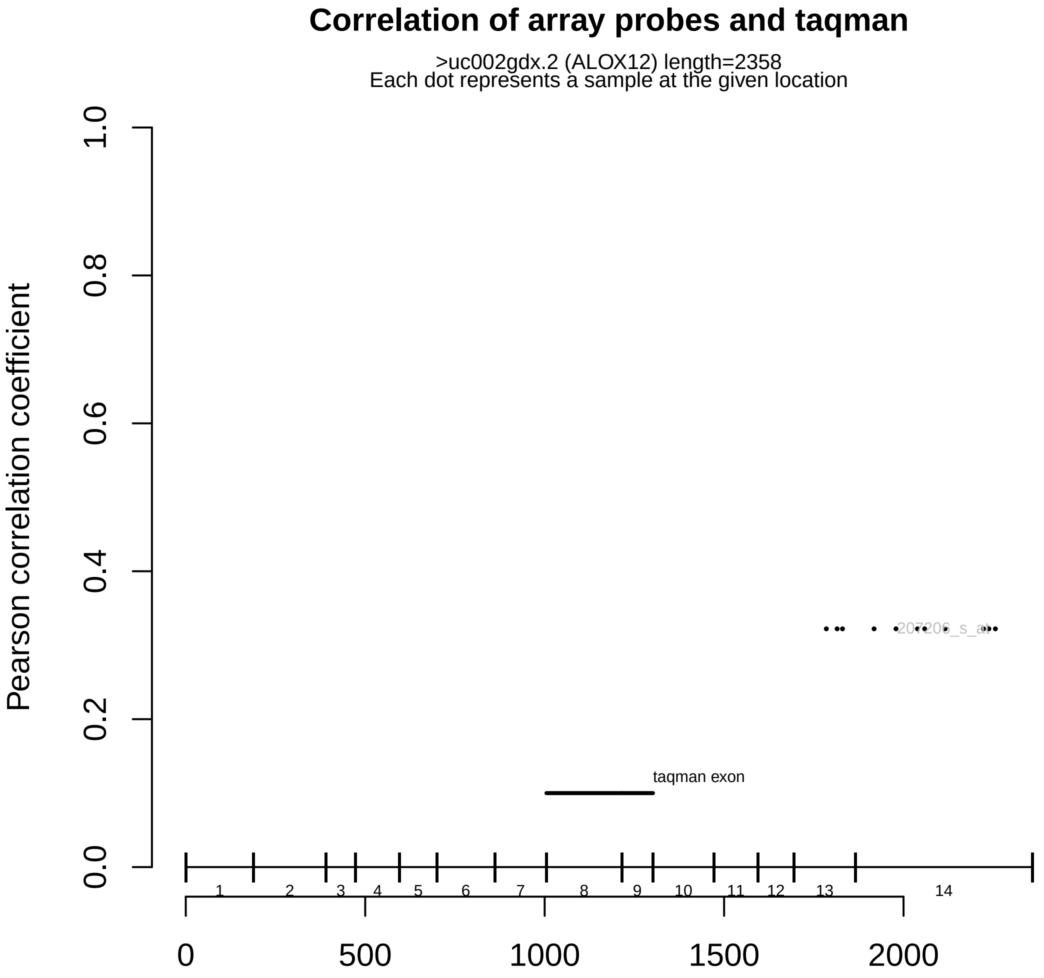

Found 1 probesets with correlation coefficient(s): 207206_s_at: 0.322

Found all probesets

Number of taqman replicate measurements with CV above threshold: 69

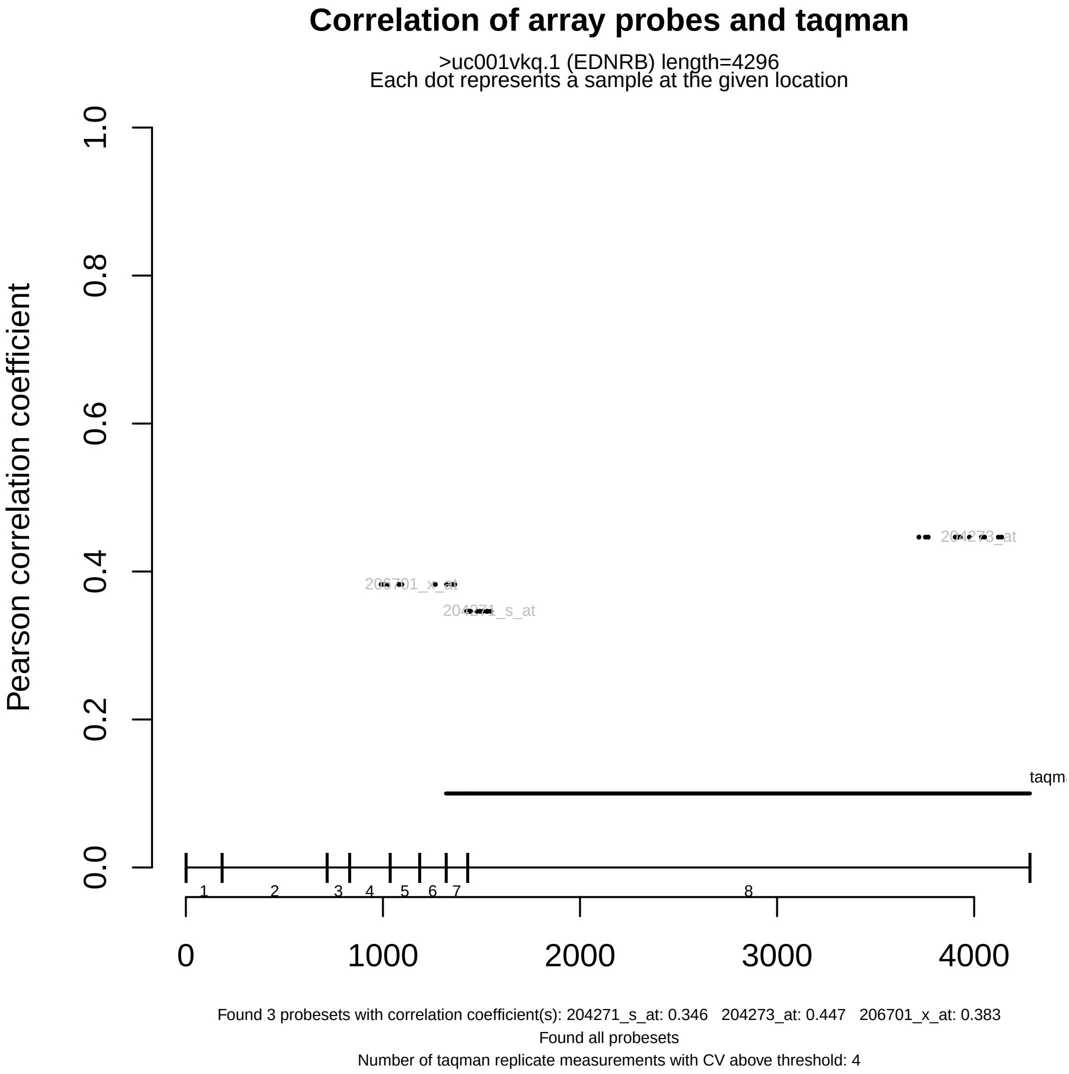

Correlation of array probes and taqman
>uc001vkq.1 (EDNRB) length=4296
Each dot represents a sample at the given location
Pearson correlation coefficient
0.0
0.2
0.4
0.6
0.8
1.0
206701_x_at
204271_s_at
204273_at
taqm
1
2
3
4
5
6
7
8
0
1000
2000
3000
4000
Found 3 probesets with correlation coefficient(s): 204271_s_at: 0.346 204273_at: 0.447 206701_x_at: 0.383
Found all probesets
Number of taqman replicate measurements with CV above threshold: 4

Correlation of array probes and taqman

>uc001tjn.2 (IGF1) length=7204
Each dot represents a sample at the given location

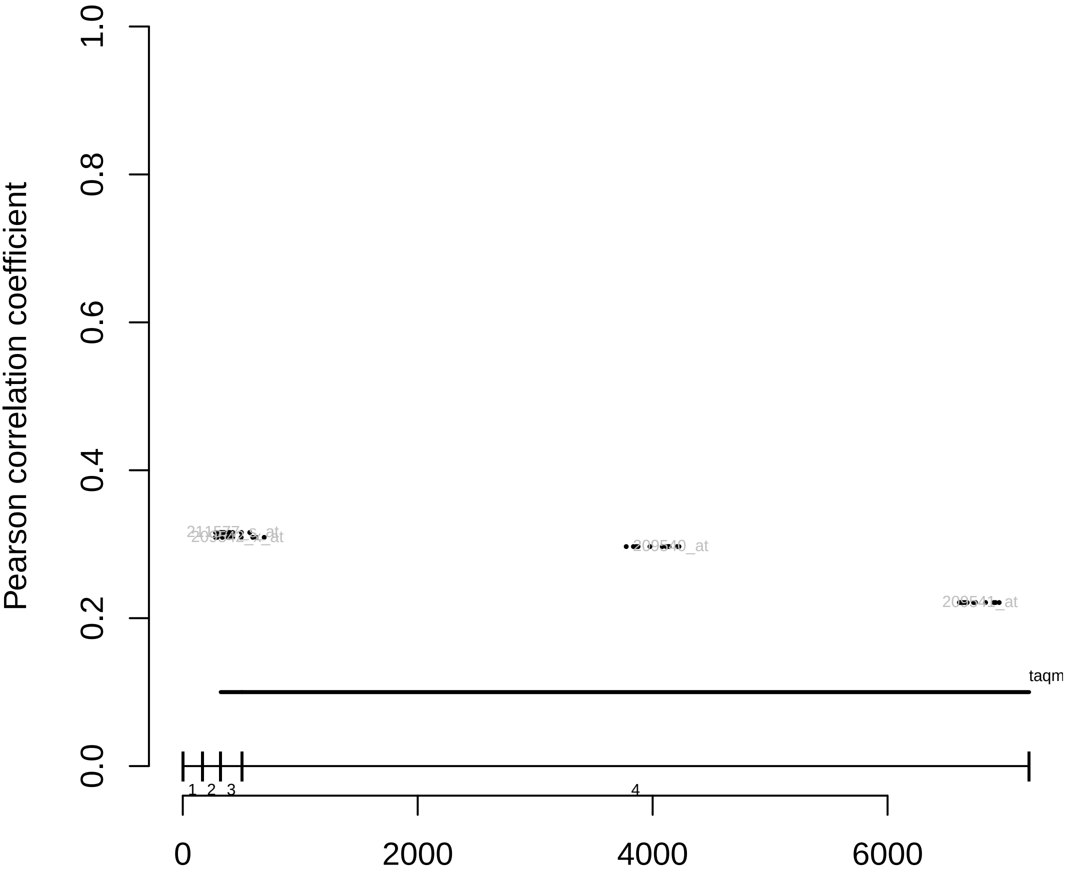

Found 4 probesets with correlation coefficient(s): 209540_at: 0.297 209541_at: 0.221 209542_x_at: 0.309 211577_s_at: 0.316
Found all probesets
Number of taqman replicate measurements with CV above threshold: 3

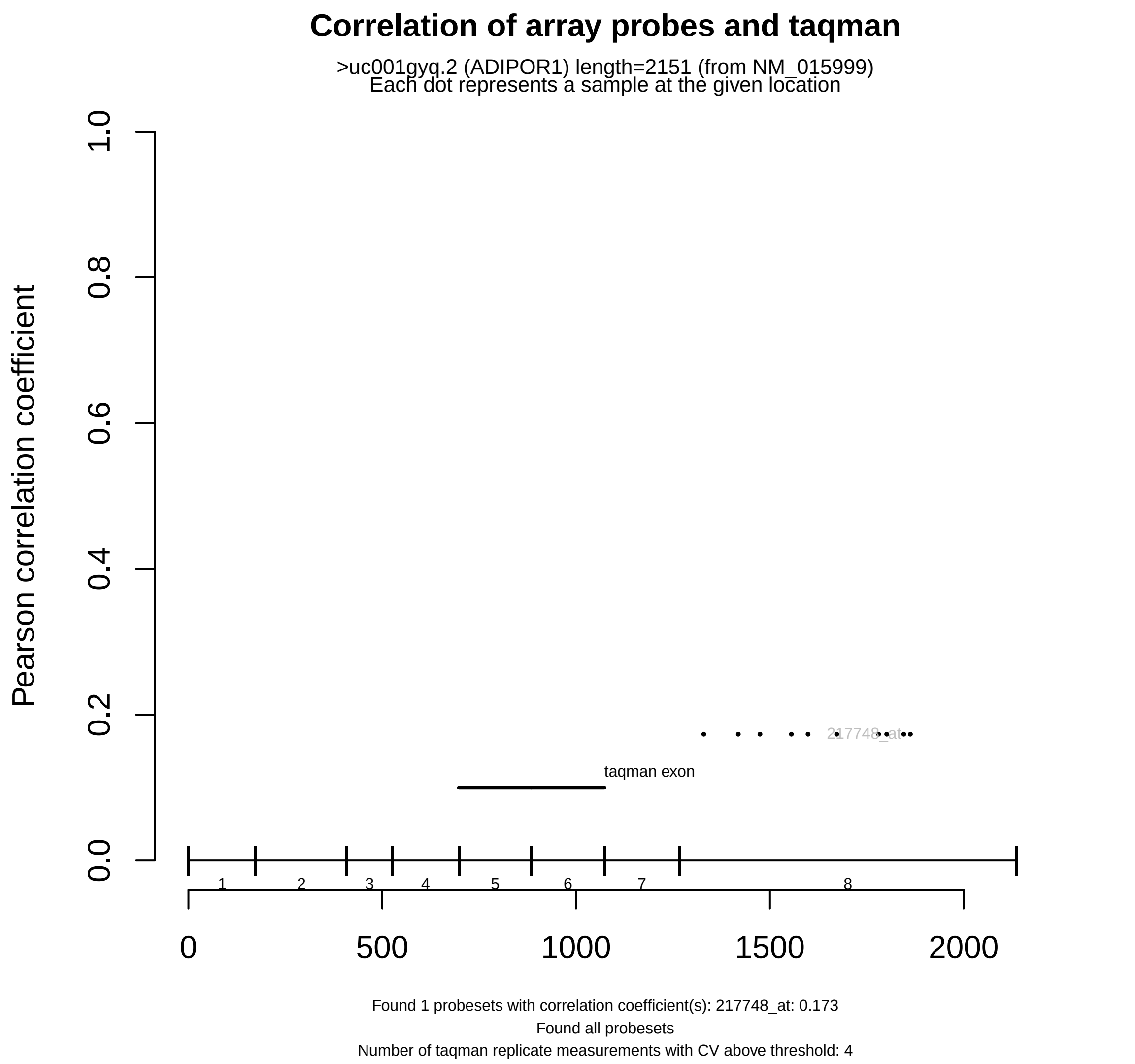
Correlation of array probes and taqman
>uc001gyq.2 (ADIPOR1) length=2151 (from NM_015999)
Each dot represents a sample at the given location
Pearson correlation coefficient
0.0
0.2
0.4
0.6
0.8
1.0
217748_at
taqman exon
1
2
3
4
5
6
7
8
0
500
1000
1500
2000
Found 1 probesets with correlation coefficient(s): 217748_at: 0.173
Found all probesets
Number of taqman replicate measurements with CV above threshold: 4
