# Supplementary material for: Endogenous control genes in complex vascular tissue samples
Source: BMC Genomics. 2009 Nov 10;10:516. doi: 10.1186/1471-2164-10-516 (PMC2779820; doi:10.1186/1471-2164-10-516)
Supplement: Additional file 5 — Figure 3, without probe set summarization. The figure text of Figure 3 applies here as well, with the only change being that microarray data were not RMA-normalized. Instead, the quantile-normalized probe level values were extracted using the GeneRegionScan package. Full details can be found in the Additional file 7 script. [file 1471-2164-10-516-S5.pdf]

Correlation of array probes and taqman

>uc003iky.2 (EDNRA) length=4334
Each dot represents correlation to taqman of an array probe at the given location

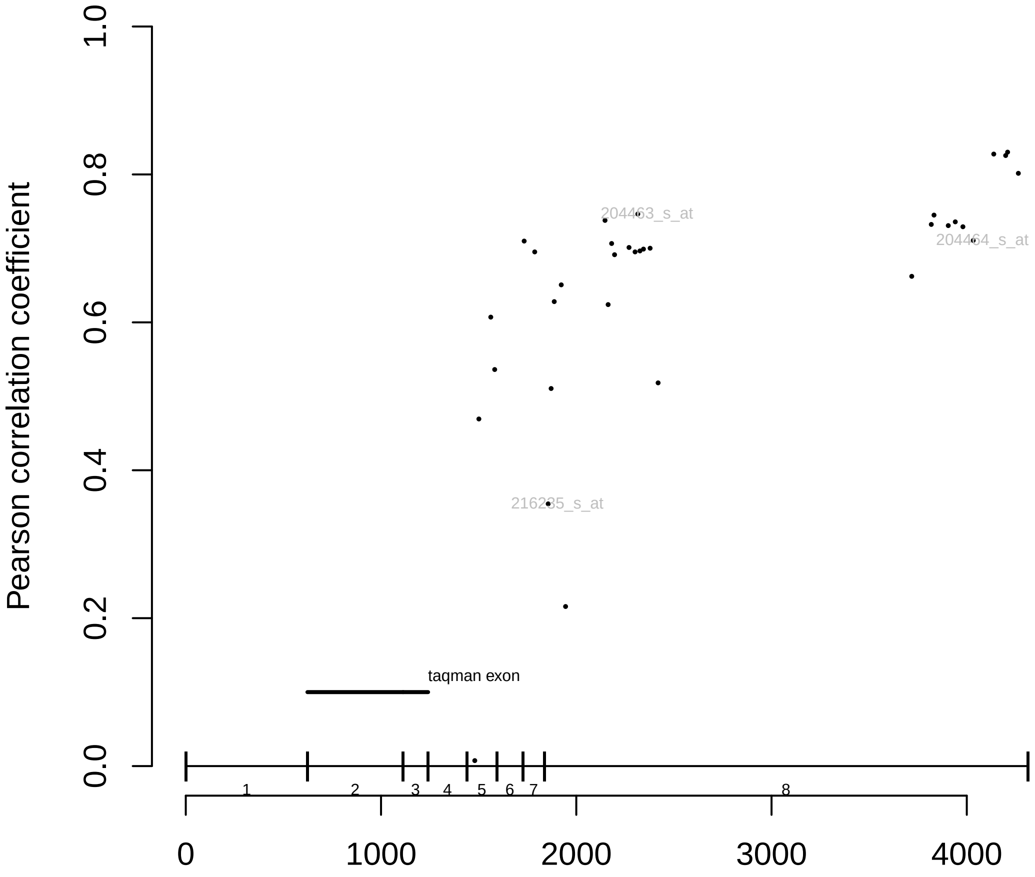

Found 3 probesets with mean correlation coefficient(s): 204463_s_at: 0.683 204464_s_at: 0.757 216235_s_at: 0.49
Found all probesets
Number of taqman replicate measurements with CV above threshold: 2

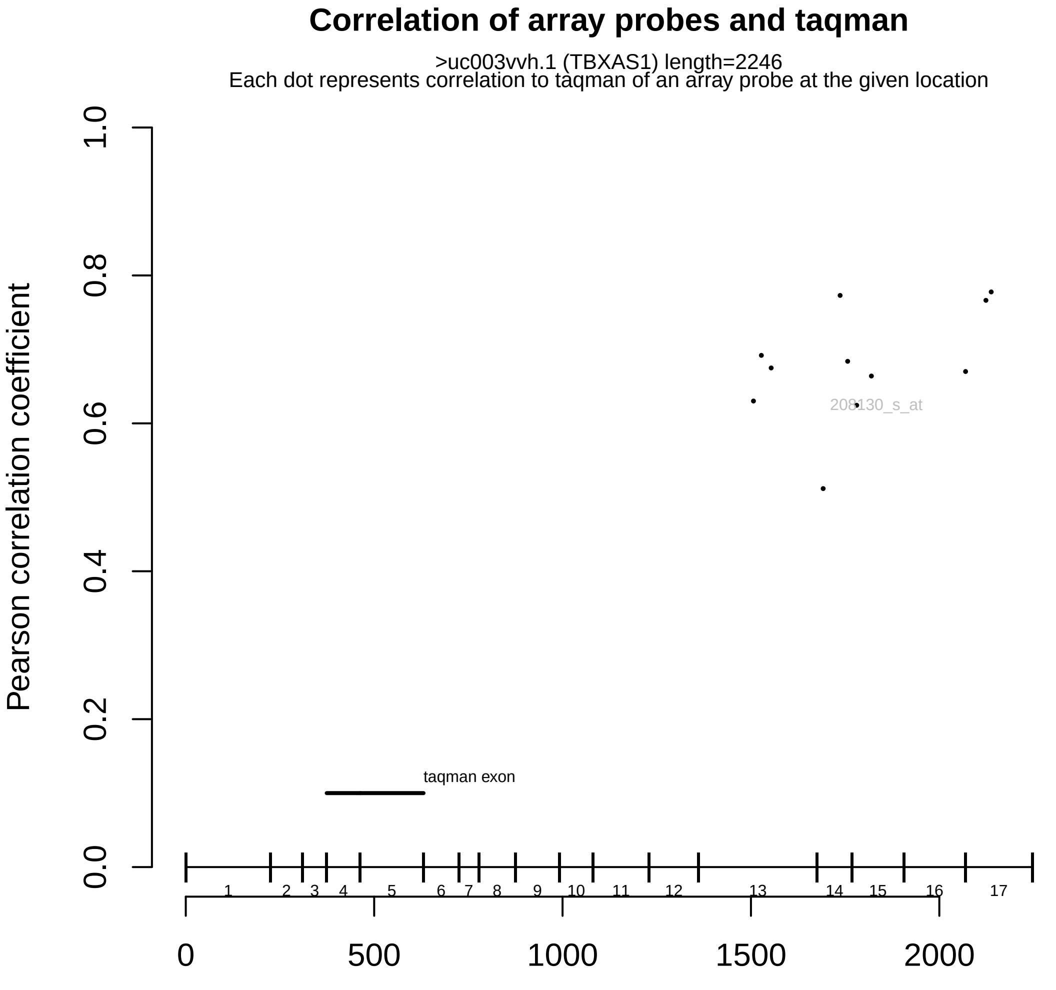

Found 1 probesets with mean correlation coefficient(s): 208130_s_at: 0.679

1 probesets did not match in the given mRNA. They had mean correlation coefficient(s): 236345_at: 0.339

Number of taqman replicate measurements with CV above threshold: 8

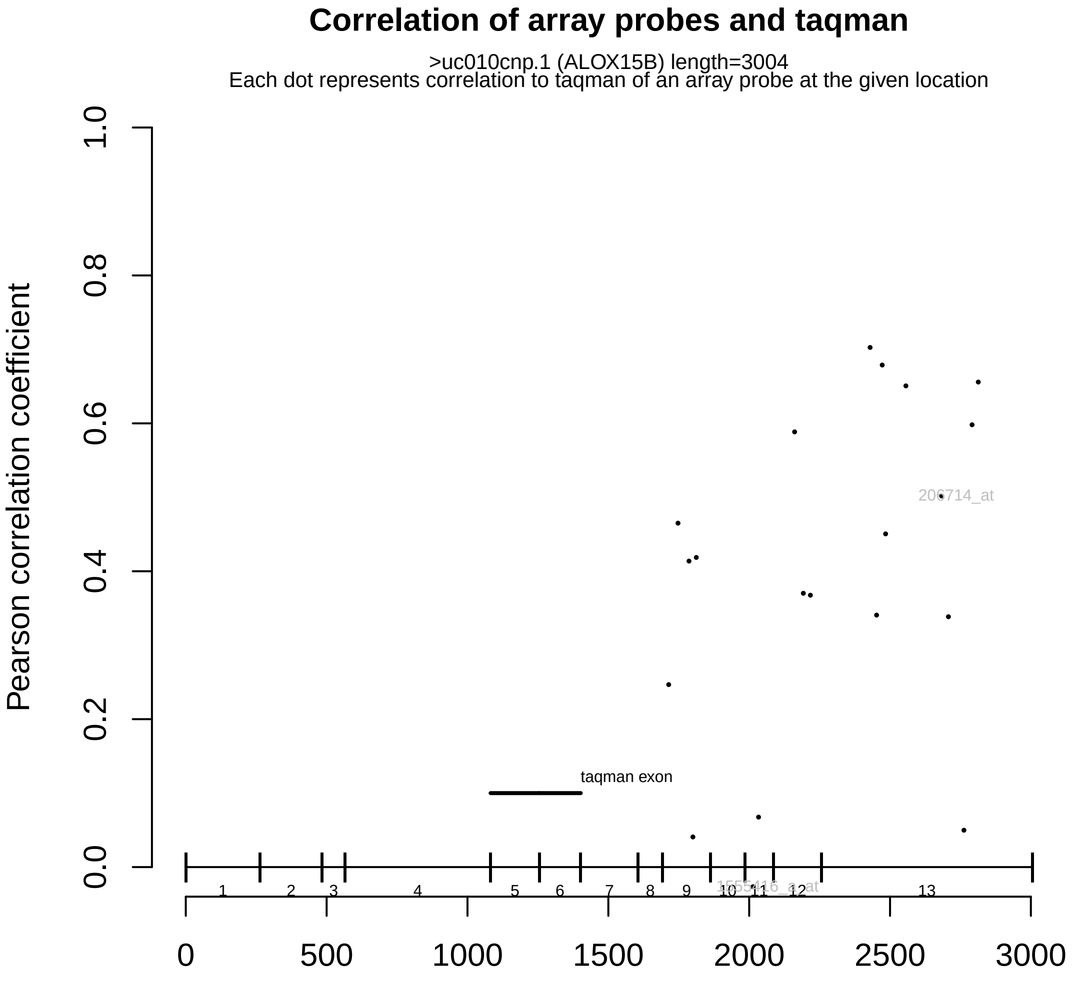

Found 2 probesets with mean correlation coefficient(s): 1555416_a_at: 0.235   206714_at: 0.481

Found all probesets

Number of taqman replicate measurements with CV above threshold: 10

Correlation of array probes and taqman

>uc003tnp.1 (IGFBP1) length=1660
Each dot represents correlation to taqman of an array probe at the given location

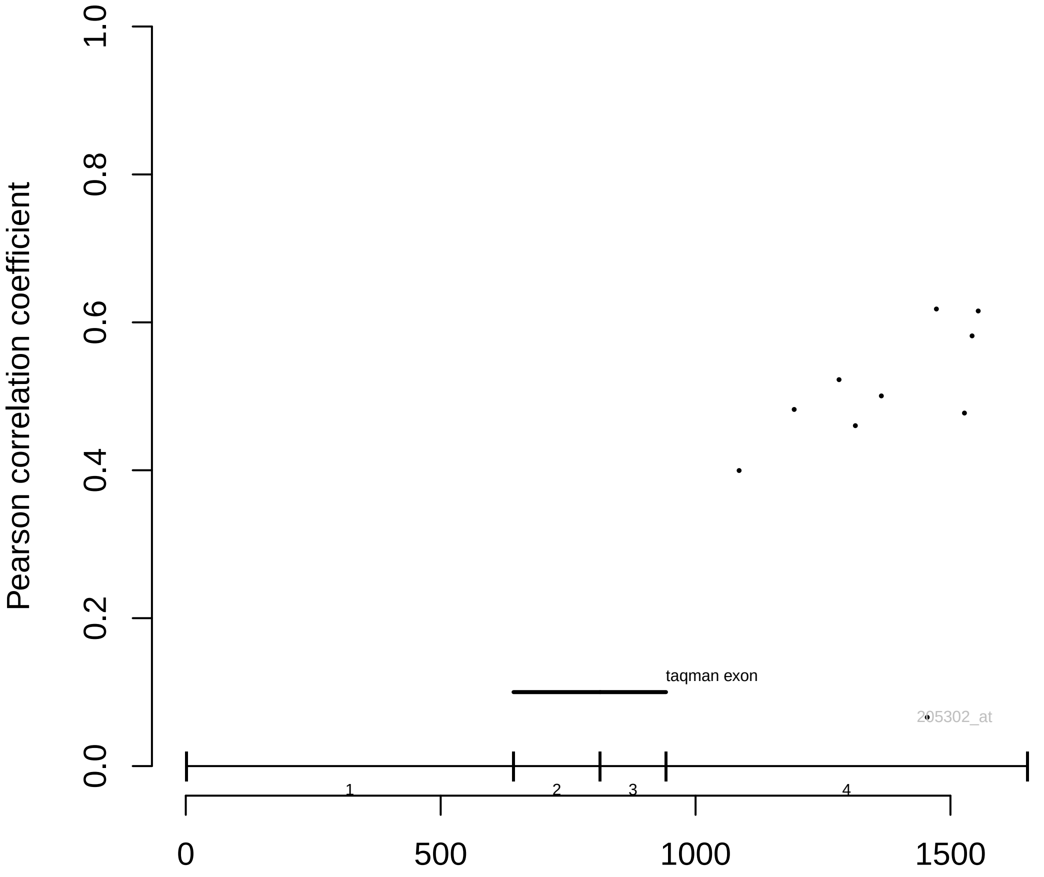

Found 1 probesets with mean correlation coefficient(s): 205302_at: 0.426
1 probesets did not match in the given mRNA. They had mean correlation coefficient(s): 237989_at: −0.00398
Number of taqman replicate measurements with CV above threshold: 7

Correlation of array probes and taqman

>uc003uhf.2 (CD36) length=4727
Each dot represents correlation to taqman of an array probe at the given location

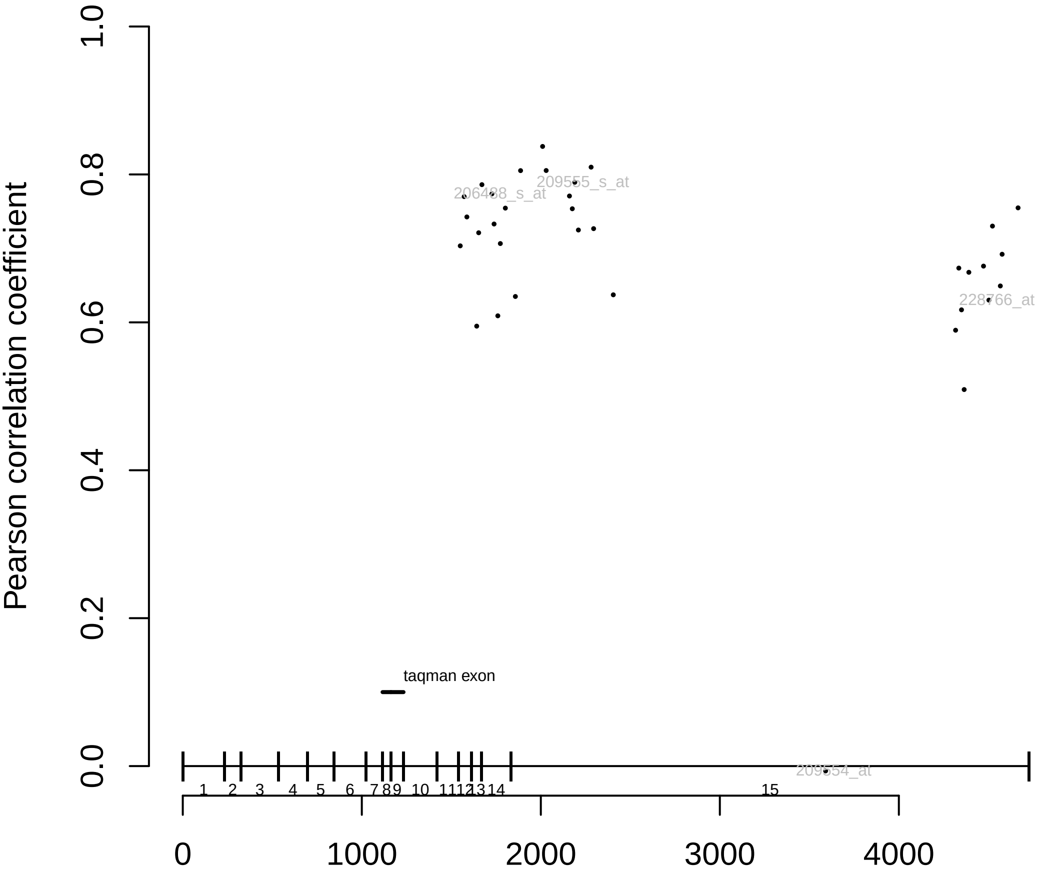

Found 4 probesets with mean correlation coefficient(s): 206488_s_at: 0.718 209554_at: 0.122 209555_s_at: 0.754 228766_at: 0.654
1 probesets did not match in the given mRNA. They had mean correlation coefficient(s): 242197_x_at: 0.575
Number of taqman replicate measurements with CV above threshold: 10

Correlation of array probes and taqman

>uc010jcr.1 (LOX) length=1016 (NM_002317)

Each dot represents correlation to taqman of an array probe at the given location

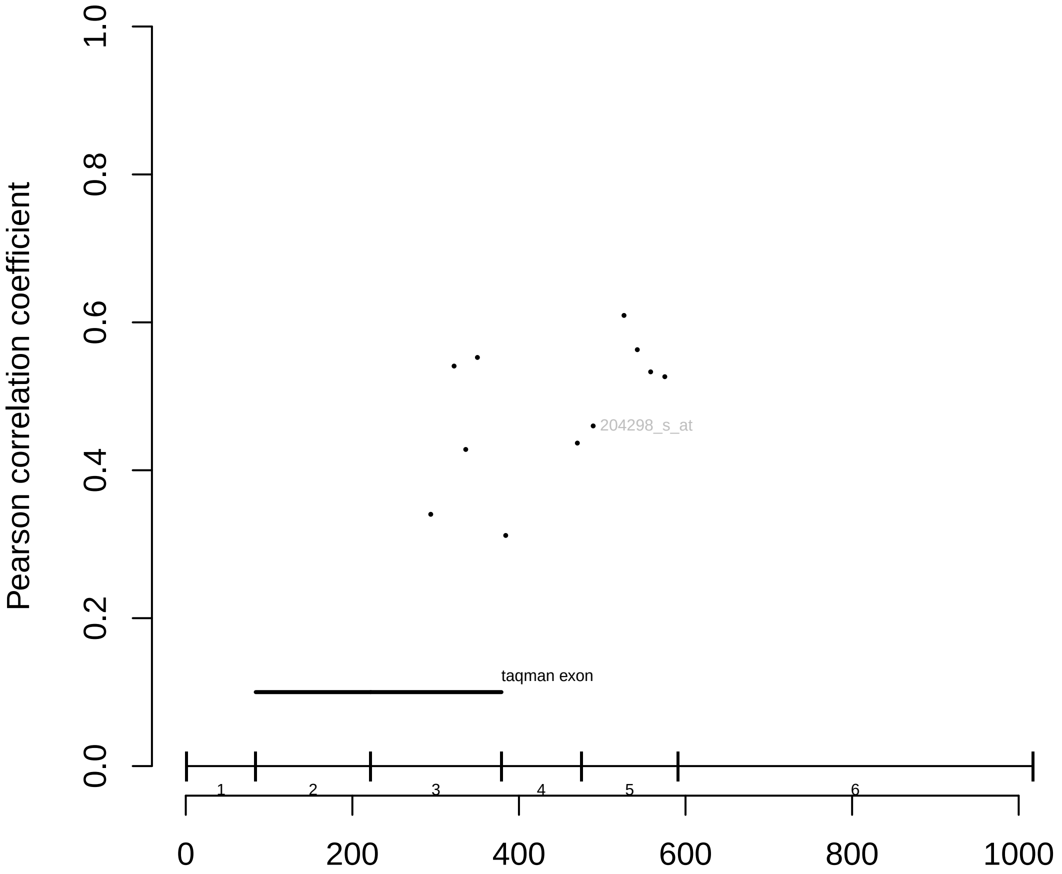

Found 1 probesets with mean correlation coefficient(s): 204298_s_at: 0.482

2 probesets did not match in the given mRNA. They had mean correlation coefficient(s): 213640_s_at: 0.169 215446_s_at: 0.624

Number of taqman replicate measurements with CV above threshold: 0

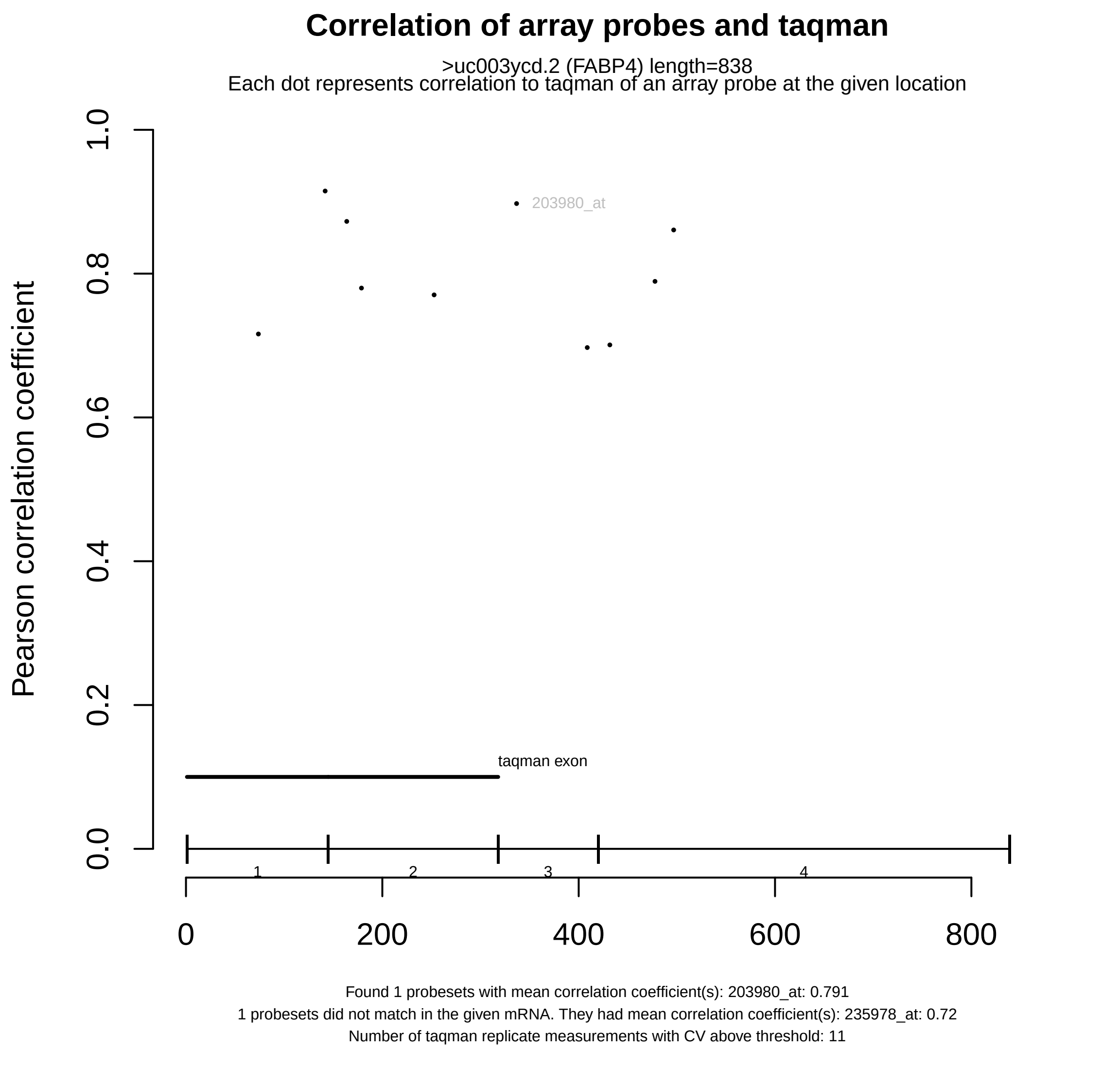

Correlation of array probes and taqman
>uc003ycd.2 (FABP4) length=838
Each dot represents correlation to taqman of an array probe at the given location
Pearson correlation coefficient
0.0
0.2
0.4
0.6
0.8
1.0
203980_at
taqman exon
1
2
3
4
0
200
400
600
800
Found 1 probesets with mean correlation coefficient(s): 203980_at: 0.791
1 probesets did not match in the given mRNA. They had mean correlation coefficient(s): 235978_at: 0.72
Number of taqman replicate measurements with CV above threshold: 11

Correlation of array probes and taqman

>uc002mfh.1 (TNFSF9 / CD137L) length=1645 (from NM_003811, inclusive 3 x G in the middle but excl poly−A tail
Each dot represents correlation to taqman of an array probe at the given location

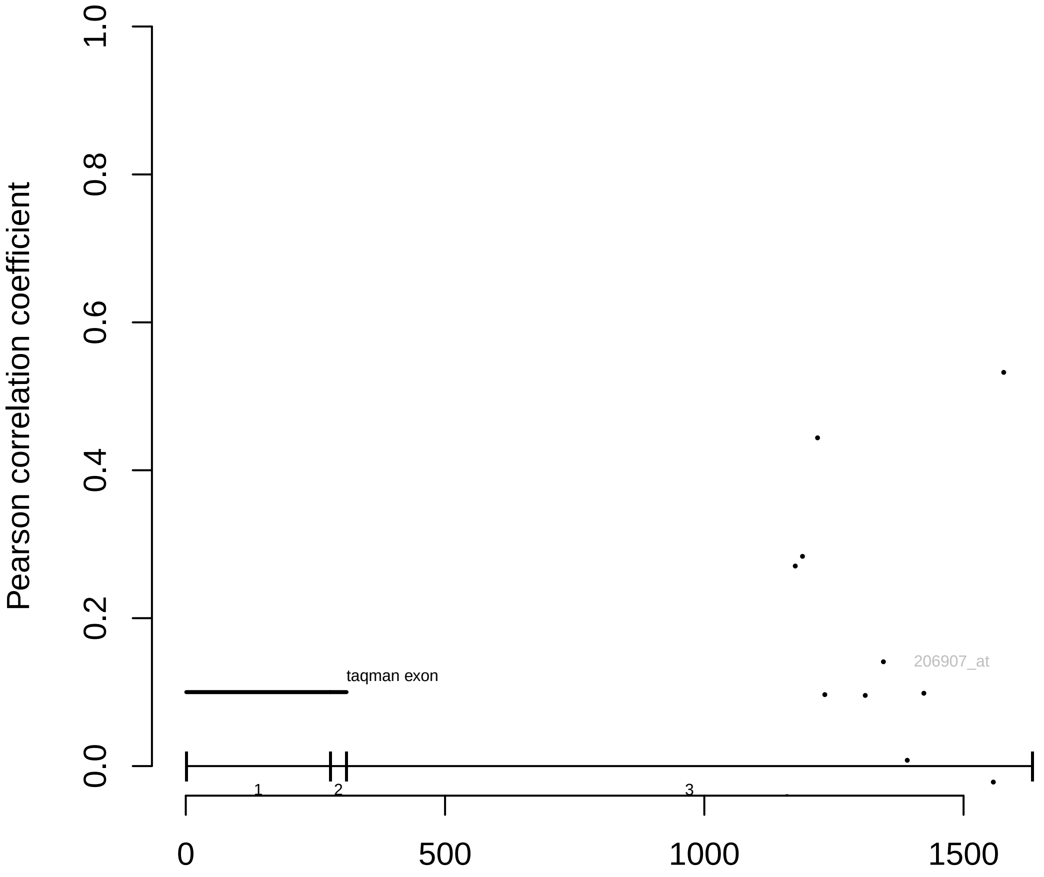

Found 1 probesets with mean correlation coefficient(s): 206907_at: 0.173

Found all probesets

Number of taqman replicate measurements with CV above threshold: 5

Correlation of array probes and taqman

>uc010bon.1 (IGF1R) length=11239
Each dot represents correlation to taqman of an array probe at the given location

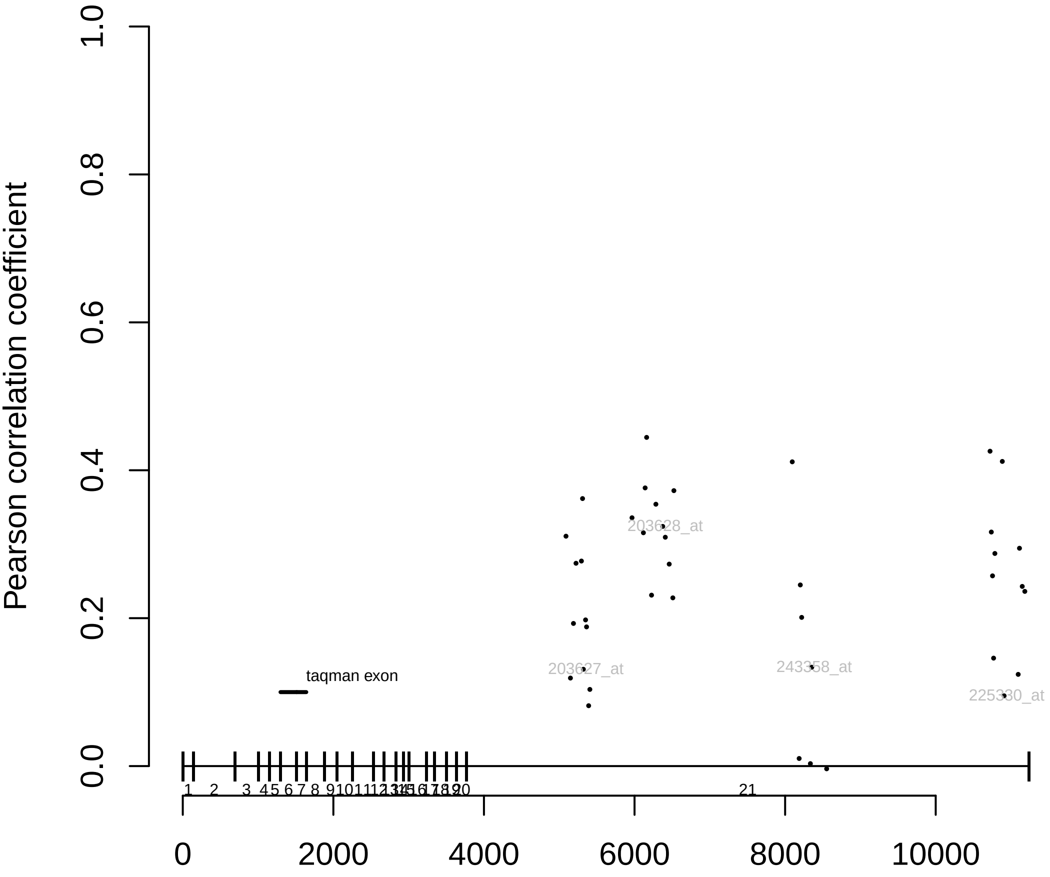

Found 4 probesets with mean correlation coefficient(s): 203627_at: 0.203 203628_at: 0.324 225330_at: 0.258 243358_at: 0.0663
1 probesets did not match in the given mRNA. They had mean correlation coefficient(s): 208441_at: 0.0248
Number of taqman replicate measurements with CV above threshold: 3

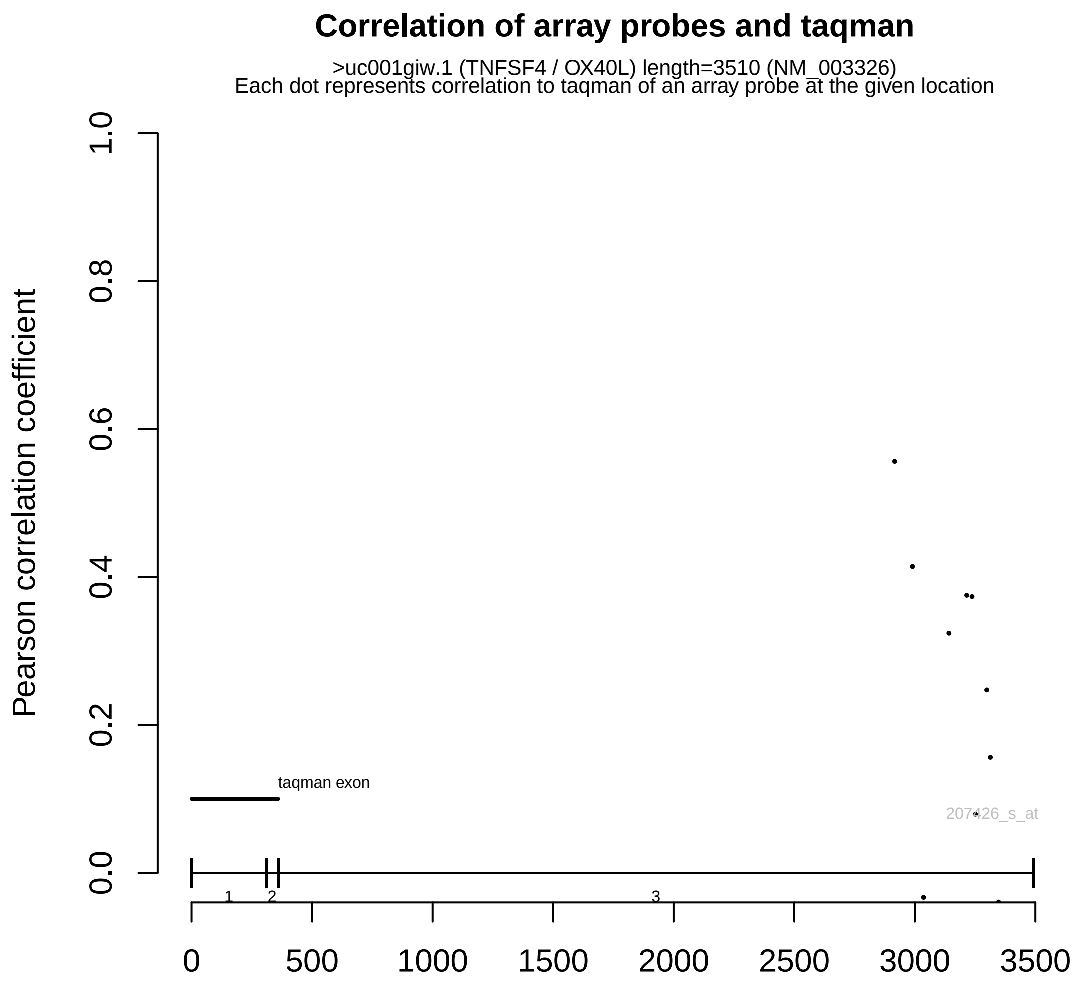

Found 1 probesets with mean correlation coefficient(s): 207426_s_at: 0.196

Found all probesets

Number of taqman replicate measurements with CV above threshold: 10

Correlation of array probes and taqman

>uc001qjn.1 (ADIPOR2) length=3973 (NM_024551)
Each dot represents correlation to taqman of an array probe at the given location

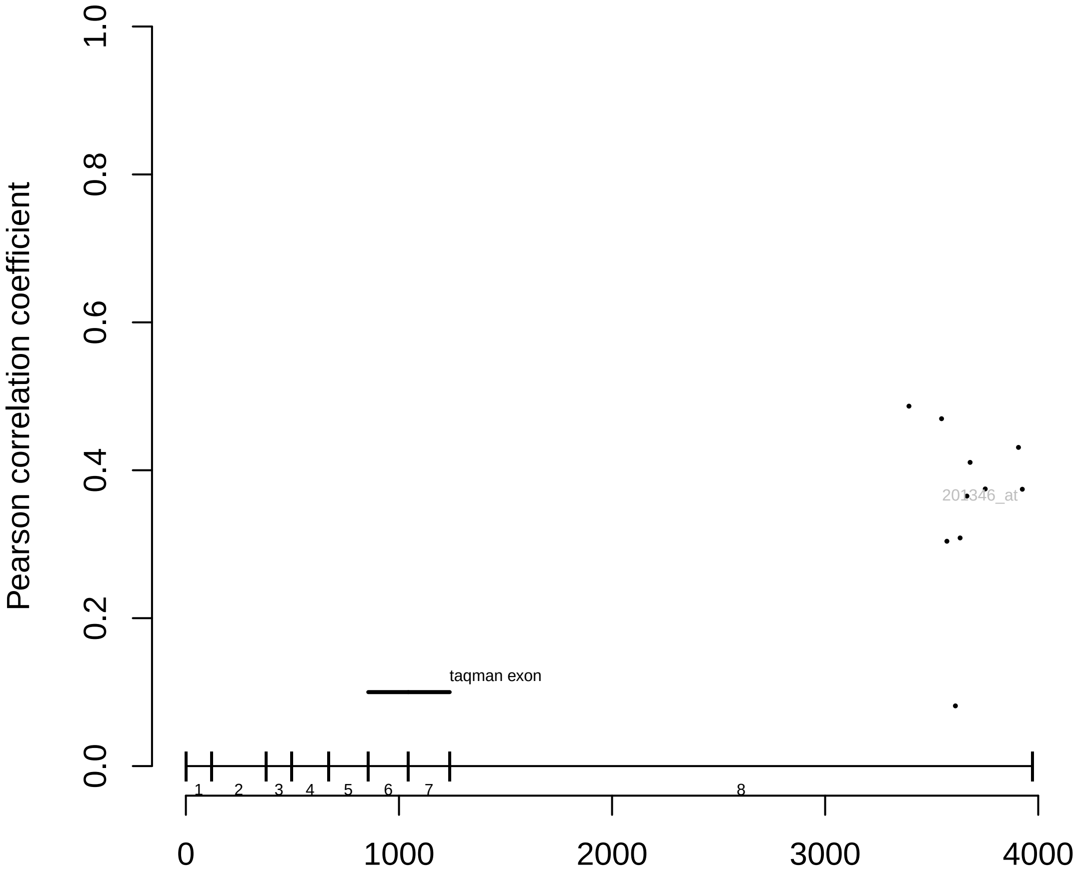

Found 1 probesets with mean correlation coefficient(s): 201346_at: 0.318
Found all probesets
Number of taqman replicate measurements with CV above threshold: 4

Correlation of array probes and taqman

>uc002gdx.2 (ALOX12) length=2358
Each dot represents correlation to taqman of an array probe at the given location

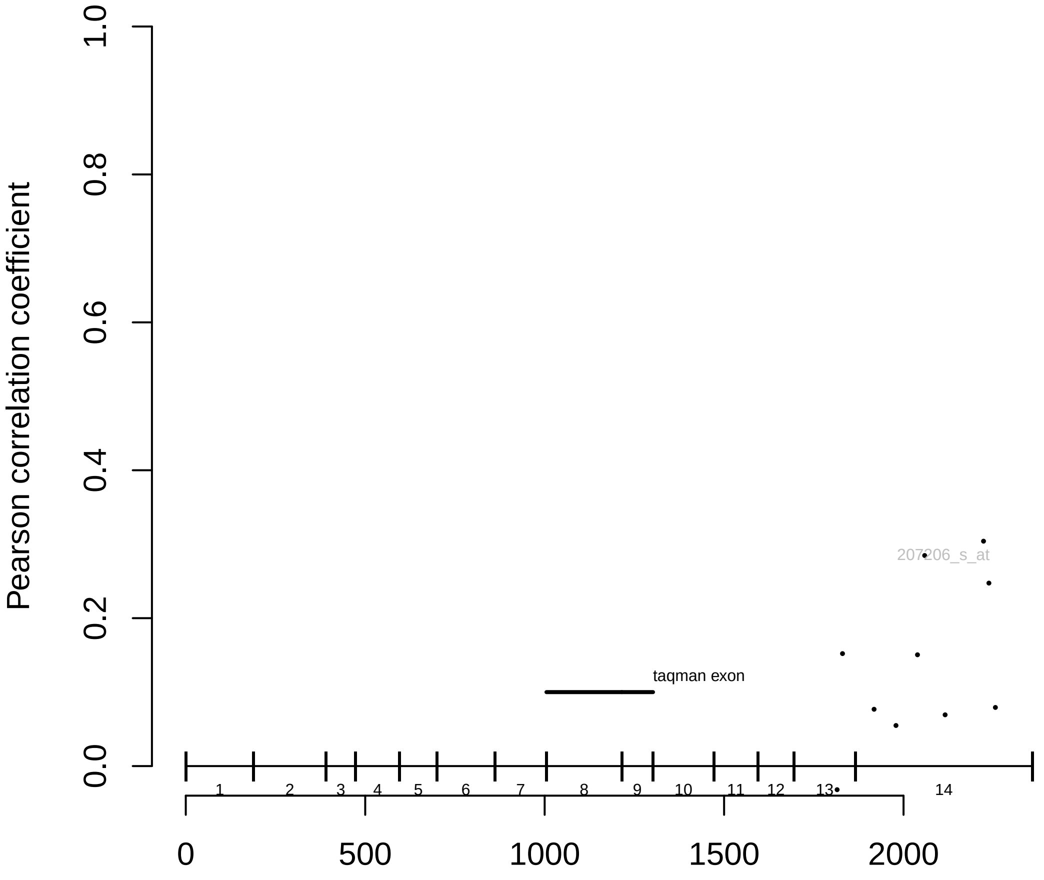

Found 1 probesets with mean correlation coefficient(s): 207206_s_at: 0.12

Found all probesets

Number of taqman replicate measurements with CV above threshold: 69

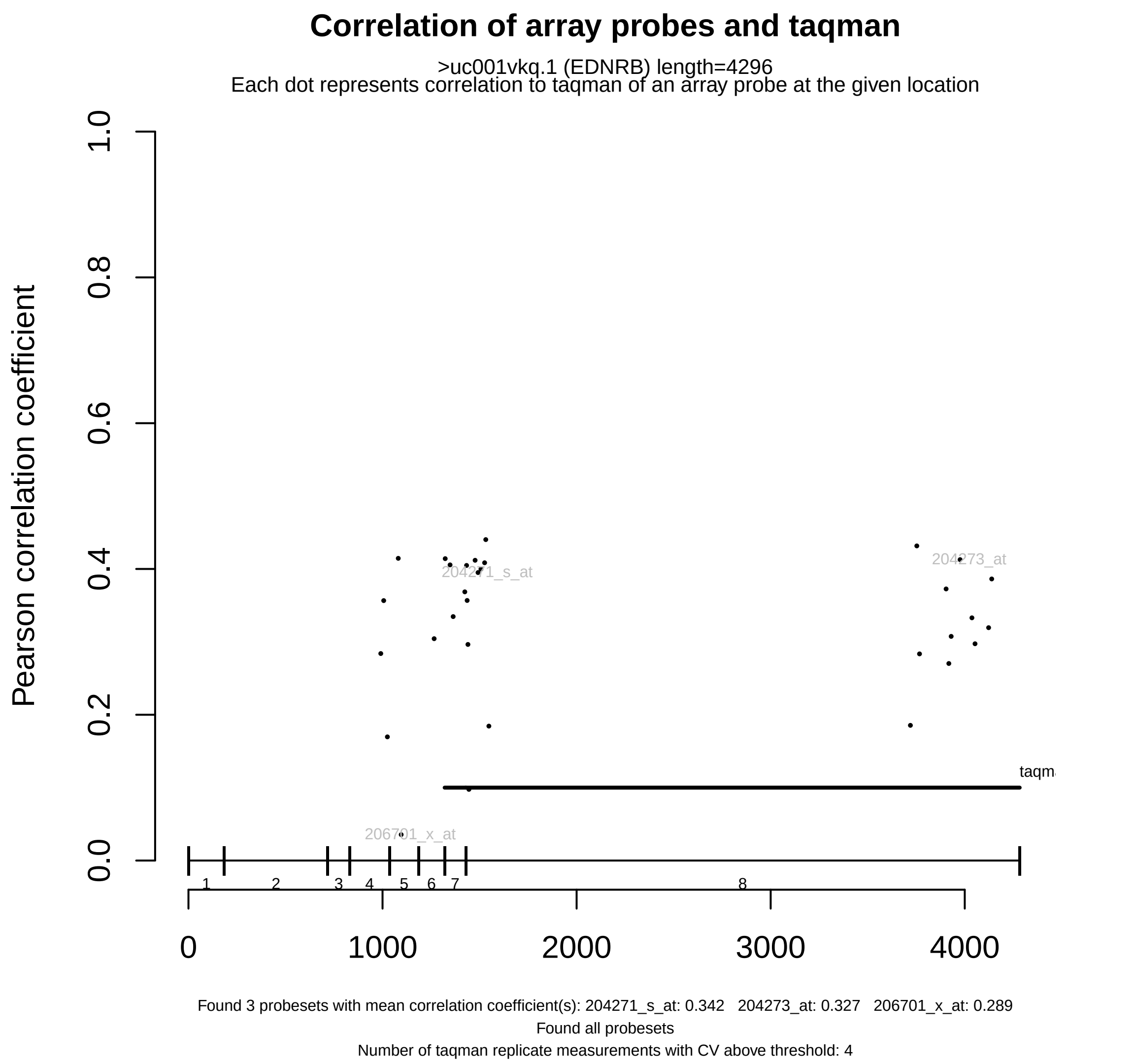

Correlation of array probes and taqman
>uc001vkq.1 (EDNRB) length=4296
Each dot represents correlation to taqman of an array probe at the given location
Pearson correlation coefficient
0.0
0.2
0.4
0.6
0.8
1.0
204271_s_at
204273_at
206701_x_at
taqm
1
2
3
4
5
6
7
8
0
1000
2000
3000
4000
Found 3 probesets with mean correlation coefficient(s): 204271_s_at: 0.342 204273_at: 0.327 206701_x_at: 0.289
Found all probesets
Number of taqman replicate measurements with CV above threshold: 4

Correlation of array probes and taqman

>uc001tjn.2 (IGF1) length=7204
Each dot represents correlation to taqman of an array probe at the given location

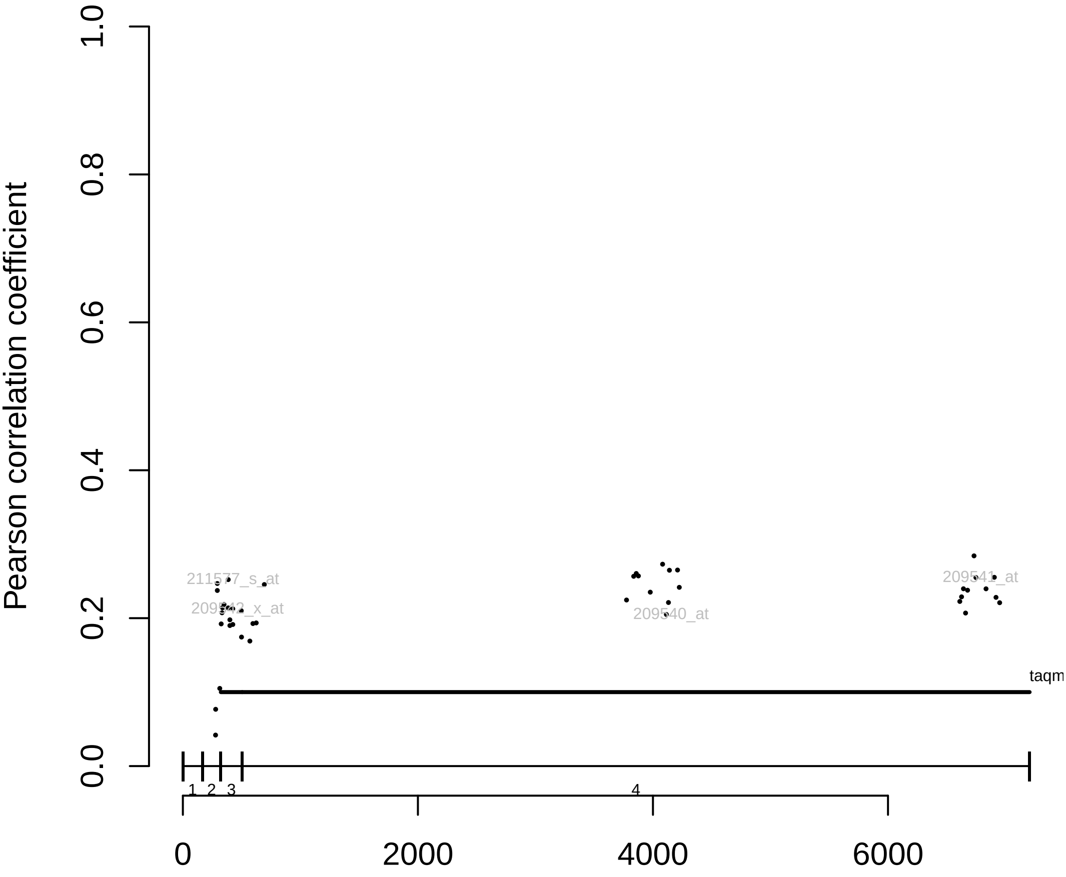

Found 4 probesets with mean correlation coefficient(s): 209540_at: 0.246 209541_at: 0.238 209542_x_at: 0.199 211577_s_at: 0.183
Found all probesets
Number of taqman replicate measurements with CV above threshold: 3

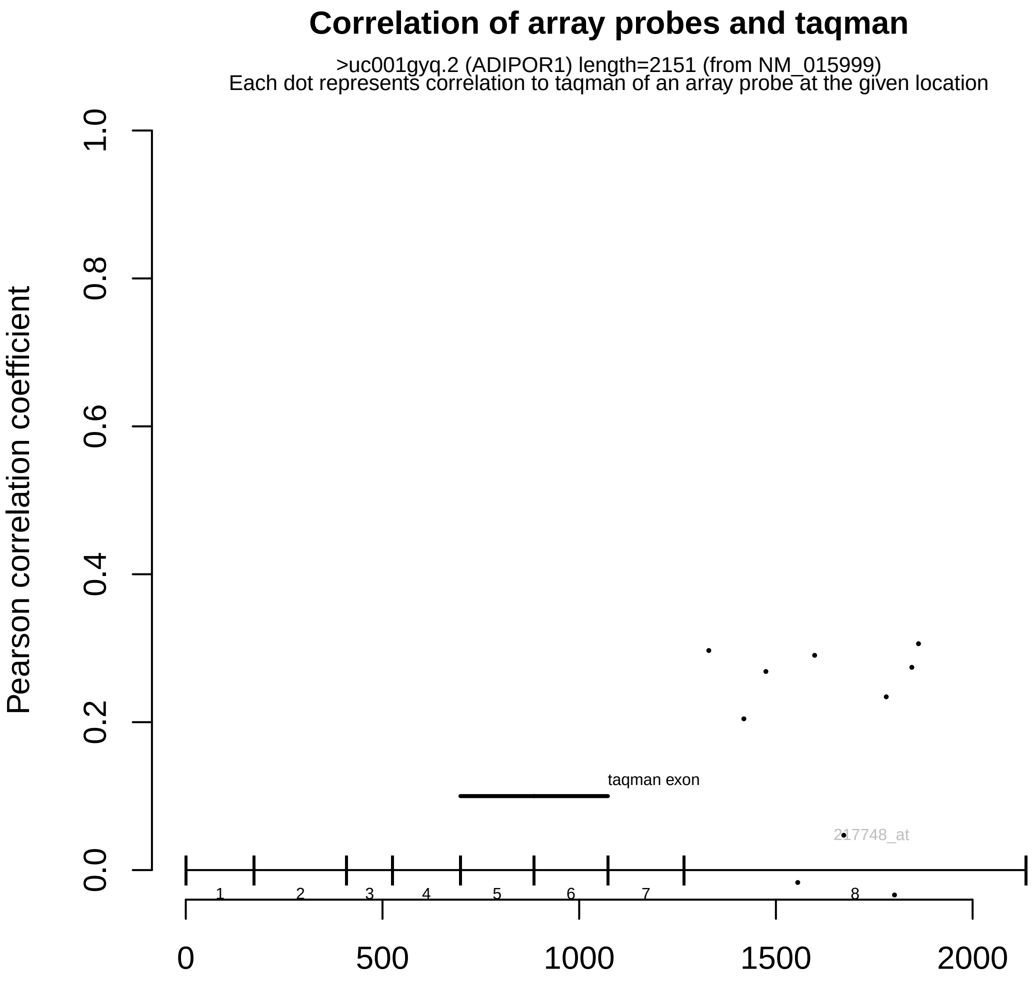

Found 1 probesets with mean correlation coefficient(s): 217748_at: 0.18

Found all probesets

Number of taqman replicate measurements with CV above threshold: 4
