# Supplementary material for: Endogenous control genes in complex vascular tissue samples
Source: BMC Genomics. 2009 Nov 10;10:516. doi: 10.1186/1471-2164-10-516 (PMC2779820; doi:10.1186/1471-2164-10-516)
Supplement: Additional file 1 — scatter plots of real-time PCR and array data for all genes of interest. The zip-file contains pdf files for each gene of interest. Each row in the pdf file shows a probe set investigating the gene in question. Each column in the pdf file shows different combinations of endogenous controls used for preparing the real-time PCR data. [file correlation plots for ADIPOR1.pdf › correlation plots for ADIPOR1.pdf]

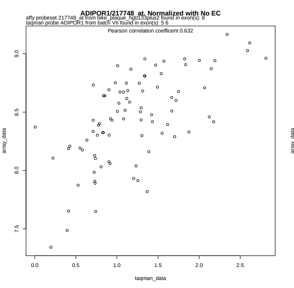
ADIPOR1/217748_at. Normalized with No EC
affy probeset 217748_at from bike_plaque_hgu133plus2 found in exon(s): 8
taqman probe ADIPOR1 from batch VII found in exon(s): 5 6
Pearson correlation coefficent 0.632
array_data
taqman_data

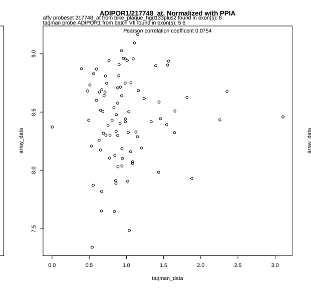
ADIPOR1/217748_at. Normalized with PPIA
affy probeset 217748_at from bike_plaque_hgu133plus2 found in exon(s): 8
taqman probe ADIPOR1 from batch VII found in exon(s): 5 6
Pearson correlation coefficent 0.0754
array_data
taqman_data

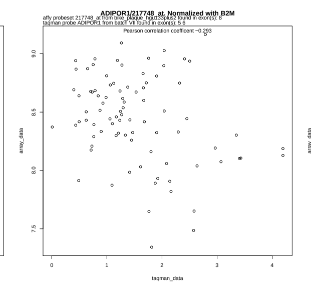
ADIPOR1/217748_at. Normalized with B2M
affy probeset 217748_at from bike_plaque_hgu133plus2 found in exon(s): 8
taqman probe ADIPOR1 from batch VII found in exon(s): 5 6
Pearson correlation coefficent −0.293
array_data
taqman_data

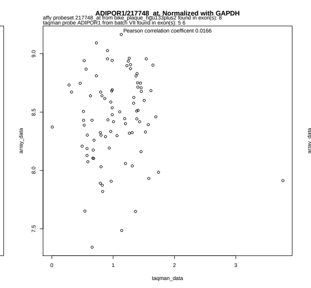
ADIPOR1/217748_at. Normalized with GAPDH
affy probeset 217748_at from bike_plaque_hgu133plus2 found in exon(s): 8
taqman probe ADIPOR1 from batch VII found in exon(s): 5 6
Pearson correlation coefficent 0.0166
array_data
taqman_data

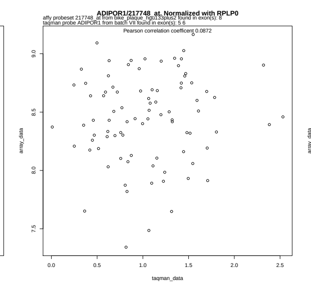
ADIPOR1/217748_at. Normalized with RPLP0
affy probeset 217748_at from bike_plaque_hgu133plus2 found in exon(s): 8
taqman probe ADIPOR1 from batch VII found in exon(s): 5 6
Pearson correlation coefficent 0.0872
array_data
taqman_data

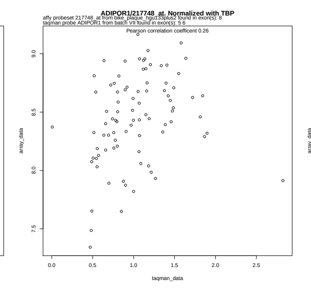
ADIPOR1/217748_at. Normalized with TBP
affy probeset 217748_at from bike_plaque_hgu133plus2 found in exon(s): 8
taqman probe ADIPOR1 from batch VII found in exon(s): 5 6
Pearson correlation coefficent 0.26
array_data
taqman_data

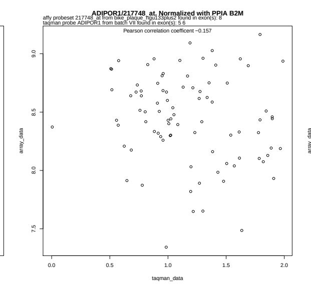
ADIPOR1/217748_at. Normalized with PPIA B2M
affy probeset 217748_at from bike_plaque_hgu133plus2 found in exon(s): 8
taqman probe ADIPOR1 from batch VII found in exon(s): 5 6
Pearson correlation coefficent −0.157
array_data
taqman_data

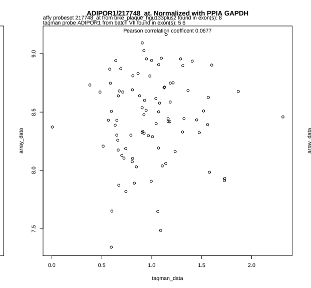
ADIPOR1/217748_at. Normalized with PPIA GAPDH
affy probeset 217748_at from bike_plaque_hgu133plus2 found in exon(s): 8
taqman probe ADIPOR1 from batch VII found in exon(s): 5 6
Pearson correlation coefficent 0.0677
array_data
taqman_data

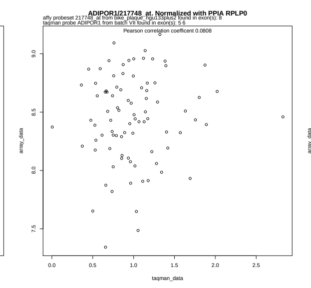
ADIPOR1/217748_at. Normalized with PPIA RPLP0
affy probeset 217748_at from bike_plaque_hgu133plus2 found in exon(s): 8
taqman probe ADIPOR1 from batch VII found in exon(s): 5 6
Pearson correlation coefficent 0.0808
array_data
taqman_data

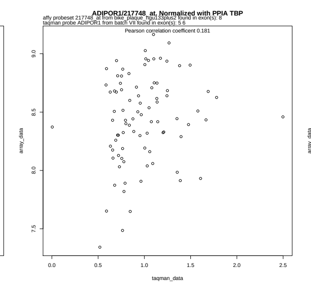
ADIPOR1/217748_at. Normalized with PPIA TBP
affy probeset 217748_at from bike_plaque_hgu133plus2 found in exon(s): 8
taqman probe ADIPOR1 from batch VII found in exon(s): 5 6
Pearson correlation coefficent 0.181
array_data
taqman_data

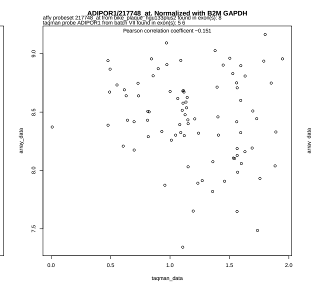
ADIPOR1/217748_at. Normalized with B2M GAPDH
affy probeset 217748_at from bike_plaque_hgu133plus2 found in exon(s): 8
taqman probe ADIPOR1 from batch VII found in exon(s): 5 6
Pearson correlation coefficent −0.151
array_data
taqman_data

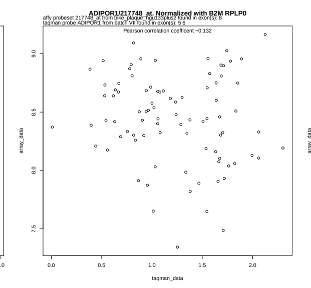
ADIPOR1/217748_at. Normalized with B2M RPLP0
affy probeset 217748_at from bike_plaque_hgu133plus2 found in exon(s): 8
taqman probe ADIPOR1 from batch VII found in exon(s): 5 6
Pearson correlation coefficent −0.132
array_data
taqman_data

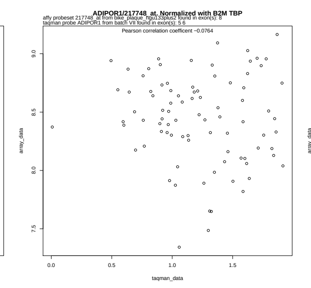
ADIPOR1/217748_at. Normalized with B2M TBP
affy probeset 217748_at from bike_plaque_hgu133plus2 found in exon(s): 8
taqman probe ADIPOR1 from batch VII found in exon(s): 5 6
Pearson correlation coefficent −0.0764
array_data
taqman_data

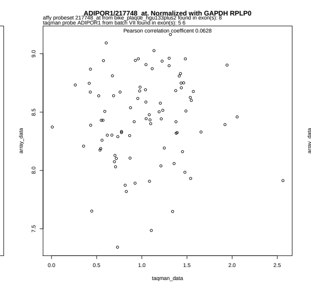
ADIPOR1/217748_at. Normalized with GAPDH RPLP0
affy probeset 217748_at from bike_plaque_hgu133plus2 found in exon(s): 8
taqman probe ADIPOR1 from batch VII found in exon(s): 5 6
Pearson correlation coefficent 0.0628
array_data
taqman_data

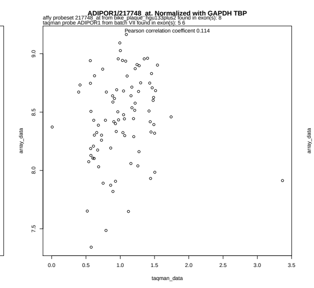
ADIPOR1/217748_at. Normalized with GAPDH TBP
affy probeset 217748_at from bike_plaque_hgu133plus2 found in exon(s): 8
taqman probe ADIPOR1 from batch VII found in exon(s): 5 6
Pearson correlation coefficent 0.114
array_data
taqman_data

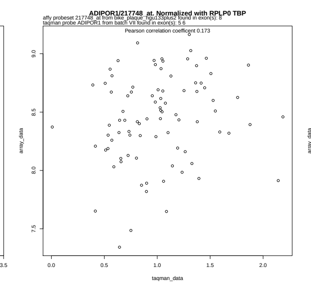
ADIPOR1/217748_at. Normalized with RPLP0 TBP
affy probeset 217748_at from bike_plaque_hgu133plus2 found in exon(s): 8
taqman probe ADIPOR1 from batch VII found in exon(s): 5 6
Pearson correlation coefficent 0.173
array_data
taqman_data

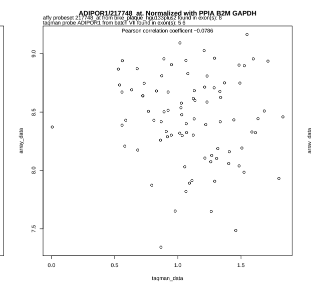
ADIPOR1/217748_at. Normalized with PPIA B2M GAPDH
affy probeset 217748_at from bike_plaque_hgu133plus2 found in exon(s): 8
taqman probe ADIPOR1 from batch VII found in exon(s): 5 6
Pearson correlation coefficent −0.0786
array_data
taqman_data

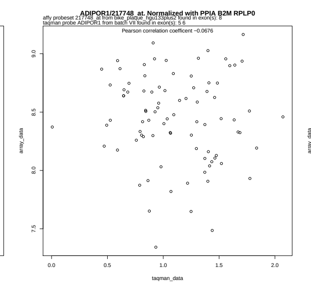
ADIPOR1/217748_at. Normalized with PPIA B2M RPLP0
affy probeset 217748_at from bike_plaque_hgu133plus2 found in exon(s): 8
taqman probe ADIPOR1 from batch VII found in exon(s): 5 6
Pearson correlation coefficent −0.0676
array_data
taqman_data

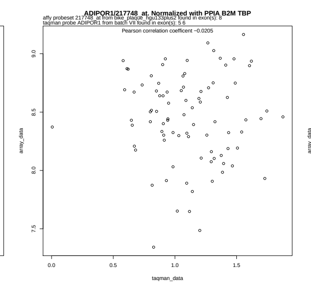
ADIPOR1/217748_at. Normalized with PPIA B2M TBP
affy probeset 217748_at from bike_plaque_hgu133plus2 found in exon(s): 8
taqman probe ADIPOR1 from batch VII found in exon(s): 5 6
Pearson correlation coefficent −0.0205
array_data
taqman_data

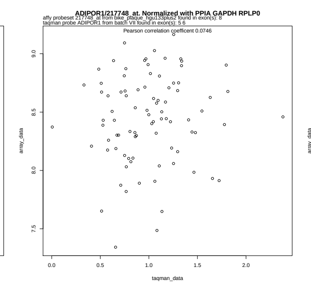
ADIPOR1/217748_at. Normalized with PPIA GAPDH RPLP0
affy probeset 217748_at from bike_plaque_hgu133plus2 found in exon(s): 8
taqman probe ADIPOR1 from batch VII found in exon(s): 5 6
Pearson correlation coefficent 0.0746
array_data
taqman_data

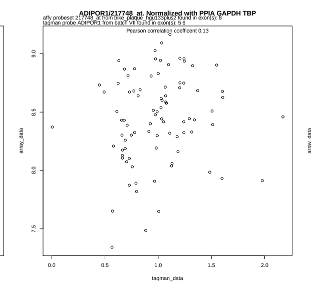
ADIPOR1/217748_at. Normalized with PPIA GAPDH TBP
affy probeset 217748_at from bike_plaque_hgu133plus2 found in exon(s): 8
taqman probe ADIPOR1 from batch VII found in exon(s): 5 6
Pearson correlation coefficent 0.13
array_data
taqman_data

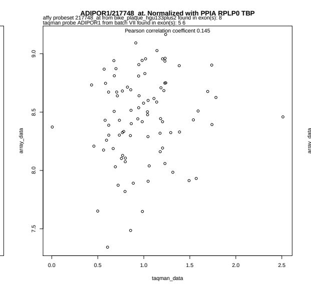
ADIPOR1/217748_at. Normalized with PPIA RPLP0 TBP
affy probeset 217748_at from bike_plaque_hgu133plus2 found in exon(s): 8
taqman probe ADIPOR1 from batch VII found in exon(s): 5 6
Pearson correlation coefficent 0.145
array_data
taqman_data

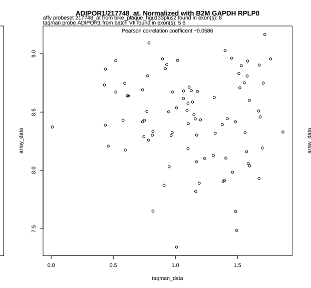
ADIPOR1/217748_at. Normalized with B2M GAPDH RPLP0
affy probeset 217748_at from bike_plaque_hgu133plus2 found in exon(s): 8
taqman probe ADIPOR1 from batch VII found in exon(s): 5 6
Pearson correlation coefficent −0.0586
array_data
taqman_data

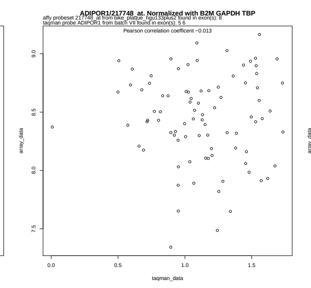
ADIPOR1/217748_at. Normalized with B2M GAPDH TBP
affy probeset 217748_at from bike_plaque_hgu133plus2 found in exon(s): 8
taqman probe ADIPOR1 from batch VII found in exon(s): 5 6
Pearson correlation coefficent −0.013
array_data
taqman_data

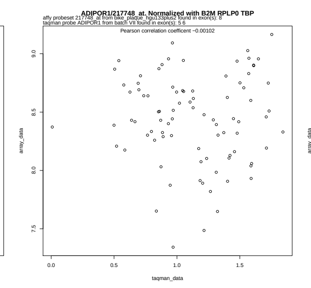
ADIPOR1/217748_at. Normalized with B2M RPLP0 TBP
affy probeset 217748_at from bike_plaque_hgu133plus2 found in exon(s): 8
taqman probe ADIPOR1 from batch VII found in exon(s): 5 6
Pearson correlation coefficent −0.00102
array_data
taqman_data

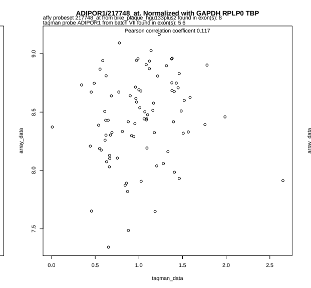
ADIPOR1/217748_at. Normalized with GAPDH RPLP0 TBP
affy probeset 217748_at from bike_plaque_hgu133plus2 found in exon(s): 8
taqman probe ADIPOR1 from batch VII found in exon(s): 5 6
Pearson correlation coefficent 0.117
array_data
taqman_data

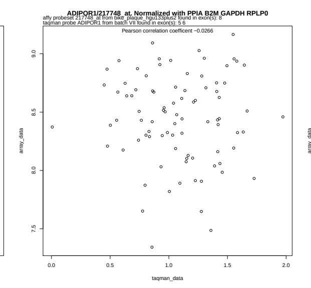
ADIPOR1/217748_at. Normalized with PPIA B2M GAPDH RPLP0
affy probeset 217748_at from bike_plaque_hgu133plus2 found in exon(s): 8
taqman probe ADIPOR1 from batch VII found in exon(s): 5 6
Pearson correlation coefficent −0.0266
array_data
taqman_data

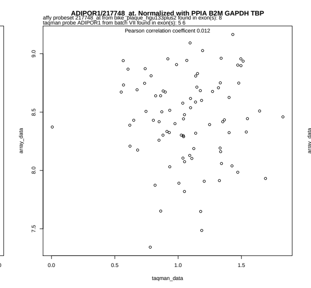
ADIPOR1/217748_at. Normalized with PPIA B2M GAPDH TBP
affy probeset 217748_at from bike_plaque_hgu133plus2 found in exon(s): 8
taqman probe ADIPOR1 from batch VII found in exon(s): 5 6
Pearson correlation coefficent 0.012
array_data
taqman_data

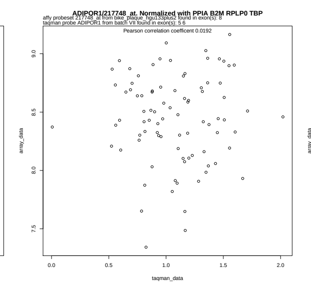
ADIPOR1/217748_at. Normalized with PPIA B2M RPLP0 TBP
affy probeset 217748_at from bike_plaque_hgu133plus2 found in exon(s): 8
taqman probe ADIPOR1 from batch VII found in exon(s): 5 6
Pearson correlation coefficent 0.0192
array_data
taqman_data

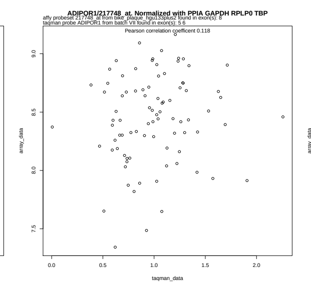
ADIPOR1/217748_at. Normalized with PPIA GAPDH RPLP0 TBP
affy probeset 217748_at from bike_plaque_hgu133plus2 found in exon(s): 8
taqman probe ADIPOR1 from batch VII found in exon(s): 5 6
Pearson correlation coefficent 0.118
array_data
taqman_data

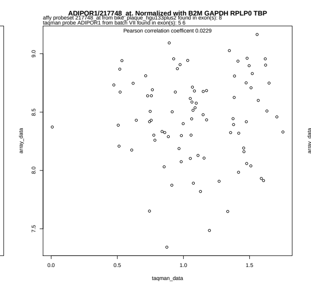
ADIPOR1/217748_at. Normalized with B2M GAPDH RPLP0 TBP
affy probeset 217748_at from bike_plaque_hgu133plus2 found in exon(s): 8
taqman probe ADIPOR1 from batch VII found in exon(s): 5 6
Pearson correlation coefficent 0.0229
array_data
taqman_data

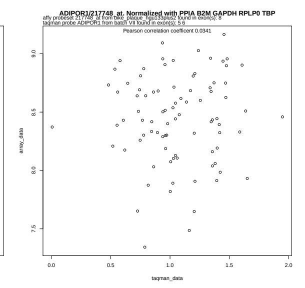
ADIPOR1/217748_at. Normalized with PPIA B2M GAPDH RPLP0 TBP
affy probeset 217748_at from bike_plaque_hgu133plus2 found in exon(s): 8
taqman probe ADIPOR1 from batch VII found in exon(s): 5 6
Pearson correlation coefficent 0.0341
array_data
taqman_data
