# Supplementary material for: Endogenous control genes in complex vascular tissue samples
Source: BMC Genomics. 2009 Nov 10;10:516. doi: 10.1186/1471-2164-10-516 (PMC2779820; doi:10.1186/1471-2164-10-516)
Supplement: Additional file 1 — scatter plots of real-time PCR and array data for all genes of interest. The zip-file contains pdf files for each gene of interest. Each row in the pdf file shows a probe set investigating the gene in question. Each column in the pdf file shows different combinations of endogenous controls used for preparing the real-time PCR data. [file correlation plots for ADIPOR2.pdf › correlation plots for ADIPOR2.pdf]

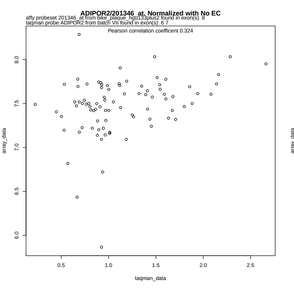
ADIPOR2/201346_at. Normalized with No EC
affy probeset 201346_at from bike_plaque_hgu133plus2 found in exon(s): 8
taqman probe ADIPOR2 from batch VII found in exon(s): 6 7
Pearson correlation coefficent 0.324
array_data
taqman_data
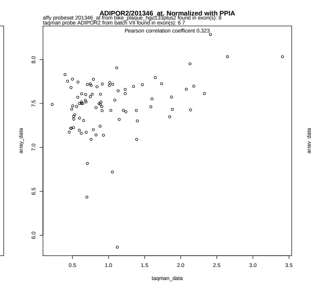
ADIPOR2/201346_at. Normalized with PPIA
affy probeset 201346_at from bike_plaque_hgu133plus2 found in exon(s): 8
taqman probe ADIPOR2 from batch VII found in exon(s): 6 7
Pearson correlation coefficent 0.323
array_data
taqman_data
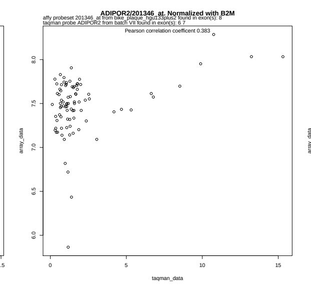
ADIPOR2/201346_at. Normalized with B2M
affy probeset 201346_at from bike_plaque_hgu133plus2 found in exon(s): 8
taqman probe ADIPOR2 from batch VII found in exon(s): 6 7
Pearson correlation coefficent 0.383
array_data
taqman_data
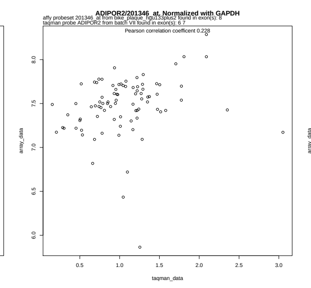
ADIPOR2/201346_at. Normalized with GAPDH
affy probeset 201346_at from bike_plaque_hgu133plus2 found in exon(s): 8
taqman probe ADIPOR2 from batch VII found in exon(s): 6 7
Pearson correlation coefficent 0.228
array_data
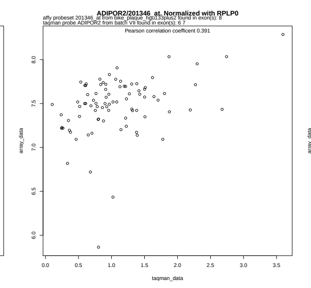
ADIPOR2/201346_at. Normalized with RPLP0
affy probeset 201346_at from bike_plaque_hgu133plus2 found in exon(s): 8
taqman probe ADIPOR2 from batch VII found in exon(s): 6 7
Pearson correlation coefficent 0.391
array_data
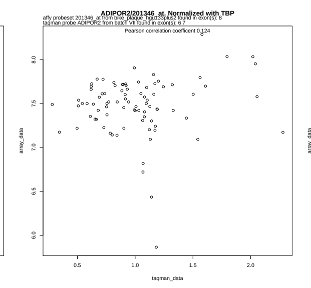
ADIPOR2/201346_at. Normalized with TBP
affy probeset 201346_at from bike_plaque_hgu133plus2 found in exon(s): 8
taqman probe ADIPOR2 from batch VII found in exon(s): 6 7
Pearson correlation coefficent 0.124
array_data
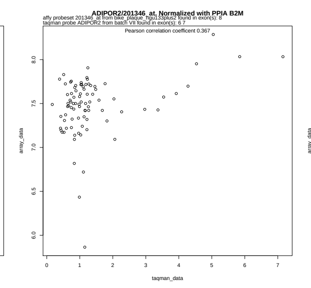
ADIPOR2/201346_at. Normalized with PPIA B2M
affy probeset 201346_at from bike_plaque_hgu133plus2 found in exon(s): 8
taqman probe ADIPOR2 from batch VII found in exon(s): 6 7
Pearson correlation coefficent 0.367
array_data
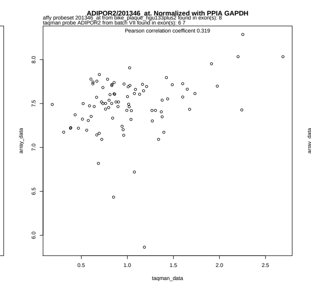
ADIPOR2/201346_at. Normalized with PPIA GAPDH
affy probeset 201346_at from bike_plaque_hgu133plus2 found in exon(s): 8
taqman probe ADIPOR2 from batch VII found in exon(s): 6 7
Pearson correlation coefficent 0.319
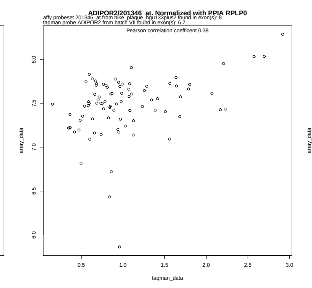
ADIPOR2/201346_at. Normalized with PPIA RPLP0
affy probeset 201346_at from bike_plaque_hgu133plus2 found in exon(s): 8
taqman probe ADIPOR2 from batch VII found in exon(s): 6 7
Pearson correlation coefficent 0.38
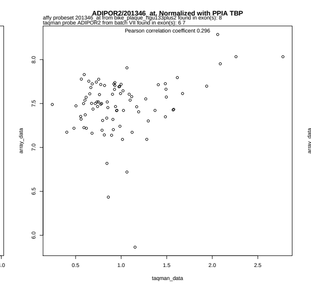
ADIPOR2/201346_at. Normalized with PPIA TBP
affy probeset 201346_at from bike_plaque_hgu133plus2 found in exon(s): 8
taqman probe ADIPOR2 from batch VII found in exon(s): 6 7
Pearson correlation coefficent 0.296
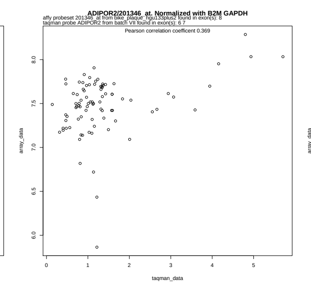
ADIPOR2/201346_at. Normalized with B2M GAPDH
affy probeset 201346_at from bike_plaque_hgu133plus2 found in exon(s): 8
taqman probe ADIPOR2 from batch VII found in exon(s): 6 7
Pearson correlation coefficent 0.369
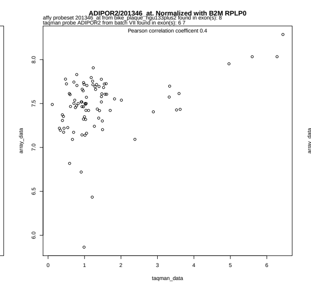
ADIPOR2/201346_at. Normalized with B2M RPLP0
affy probeset 201346_at from bike_plaque_hgu133plus2 found in exon(s): 8
taqman probe ADIPOR2 from batch VII found in exon(s): 6 7
Pearson correlation coefficent 0.4
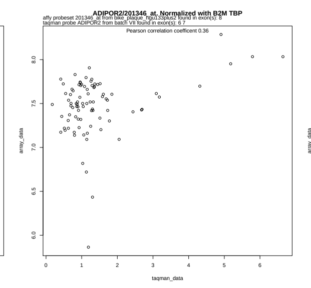
ADIPOR2/201346_at. Normalized with B2M TBP
affy probeset 201346_at from bike_plaque_hgu133plus2 found in exon(s): 8
taqman probe ADIPOR2 from batch VII found in exon(s): 6 7
Pearson correlation coefficent 0.36
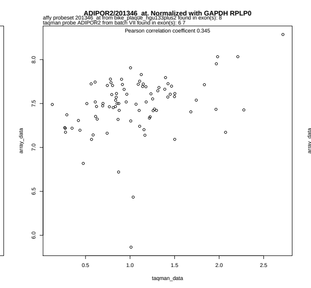
ADIPOR2/201346_at. Normalized with GAPDH RPLP0
affy probeset 201346_at from bike_plaque_hgu133plus2 found in exon(s): 8
taqman probe ADIPOR2 from batch VII found in exon(s): 6 7
Pearson correlation coefficent 0.345
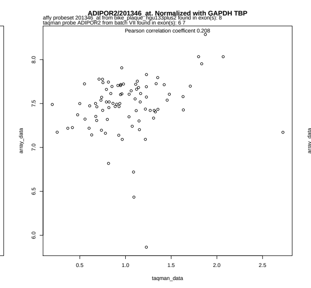
ADIPOR2/201346_at. Normalized with GAPDH TBP
affy probeset 201346_at from bike_plaque_hgu133plus2 found in exon(s): 8
taqman probe ADIPOR2 from batch VII found in exon(s): 6 7
Pearson correlation coefficent 0.208
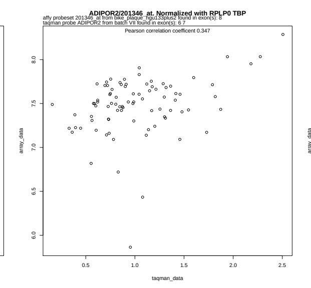
ADIPOR2/201346_at. Normalized with RPLP0 TBP
affy probeset 201346_at from bike_plaque_hgu133plus2 found in exon(s): 8
taqman probe ADIPOR2 from batch VII found in exon(s): 6 7
Pearson correlation coefficent 0.347
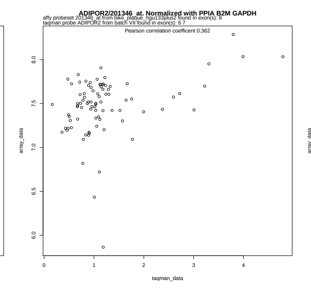
ADIPOR2/201346_at. Normalized with PPIA B2M GAPDH
affy probeset 201346_at from bike_plaque_hgu133plus2 found in exon(s): 8
taqman probe ADIPOR2 from batch VII found in exon(s): 6 7
Pearson correlation coefficent 0.362
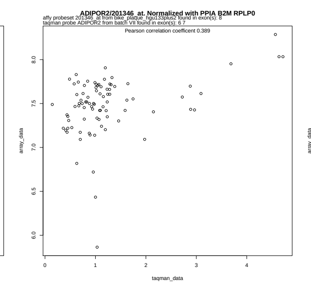
ADIPOR2/201346_at. Normalized with PPIA B2M RPLP0
affy probeset 201346_at from bike_plaque_hgu133plus2 found in exon(s): 8
taqman probe ADIPOR2 from batch VII found in exon(s): 6 7
Pearson correlation coefficent 0.389
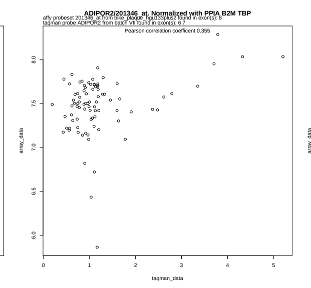
ADIPOR2/201346_at. Normalized with PPIA B2M TBP
affy probeset 201346_at from bike_plaque_hgu133plus2 found in exon(s): 8
taqman probe ADIPOR2 from batch VII found in exon(s): 6 7
Pearson correlation coefficent 0.355
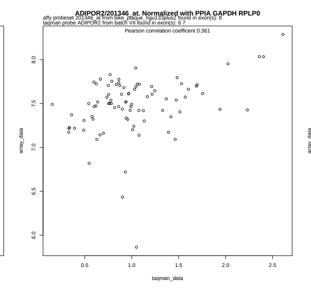
ADIPOR2/201346_at. Normalized with PPIA GAPDH RPLP0
affy probeset 201346_at from bike_plaque_hgu133plus2 found in exon(s): 8
taqman probe ADIPOR2 from batch VII found in exon(s): 6 7
Pearson correlation coefficent 0.361
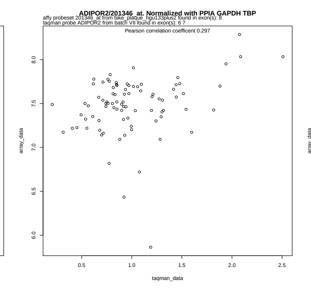
ADIPOR2/201346_at. Normalized with PPIA GAPDH TBP
affy probeset 201346_at from bike_plaque_hgu133plus2 found in exon(s): 8
taqman probe ADIPOR2 from batch VII found in exon(s): 6 7
Pearson correlation coefficent 0.297
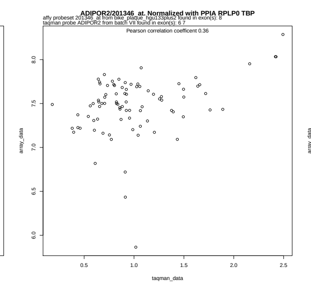
ADIPOR2/201346_at. Normalized with PPIA RPLP0 TBP
affy probeset 201346_at from bike_plaque_hgu133plus2 found in exon(s): 8
taqman probe ADIPOR2 from batch VII found in exon(s): 6 7
Pearson correlation coefficent 0.36
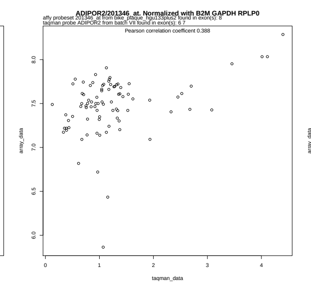
ADIPOR2/201346_at. Normalized with B2M GAPDH RPLP0
affy probeset 201346_at from bike_plaque_hgu133plus2 found in exon(s): 8
taqman probe ADIPOR2 from batch VII found in exon(s): 6 7
Pearson correlation coefficent 0.388
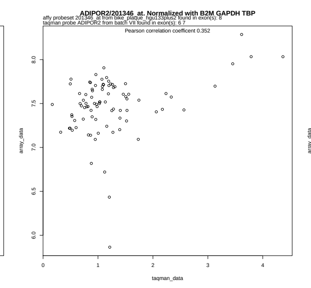
ADIPOR2/201346_at. Normalized with B2M GAPDH TBP
affy probeset 201346_at from bike_plaque_hgu133plus2 found in exon(s): 8
taqman probe ADIPOR2 from batch VII found in exon(s): 6 7
Pearson correlation coefficent 0.352
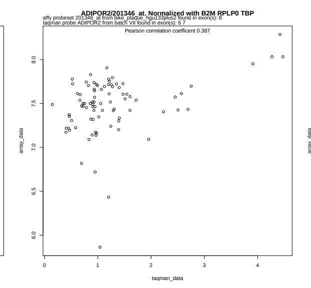
ADIPOR2/201346_at. Normalized with B2M RPLP0 TBP
affy probeset 201346_at from bike_plaque_hgu133plus2 found in exon(s): 8
taqman probe ADIPOR2 from batch VII found in exon(s): 6 7
Pearson correlation coefficent 0.387
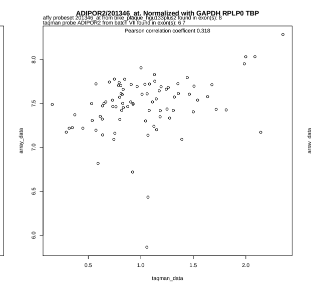
ADIPOR2/201346_at. Normalized with GAPDH RPLP0 TBP
affy probeset 201346_at from bike_plaque_hgu133plus2 found in exon(s): 8
taqman probe ADIPOR2 from batch VII found in exon(s): 6 7
Pearson correlation coefficent 0.318
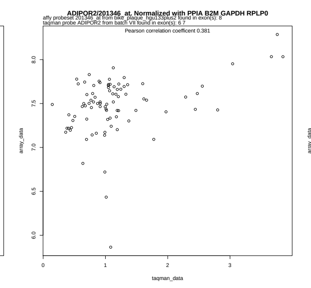
ADIPOR2/201346_at. Normalized with PPIA B2M GAPDH RPLP0
affy probeset 201346_at from bike_plaque_hgu133plus2 found in exon(s): 8
taqman probe ADIPOR2 from batch VII found in exon(s): 6 7
Pearson correlation coefficent 0.381
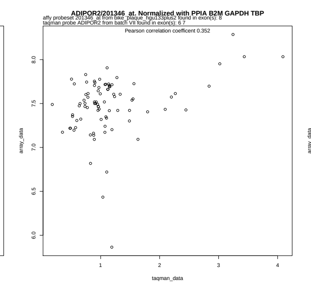
ADIPOR2/201346_at. Normalized with PPIA B2M GAPDH TBP
affy probeset 201346_at from bike_plaque_hgu133plus2 found in exon(s): 8
taqman probe ADIPOR2 from batch VII found in exon(s): 6 7
Pearson correlation coefficent 0.352
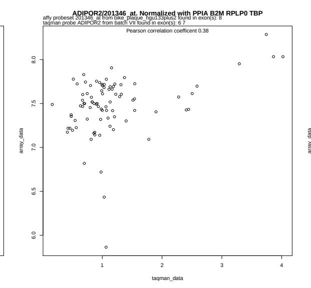
ADIPOR2/201346_at. Normalized with PPIA B2M RPLP0 TBP
affy probeset 201346_at from bike_plaque_hgu133plus2 found in exon(s): 8
taqman probe ADIPOR2 from batch VII found in exon(s): 6 7
Pearson correlation coefficent 0.38
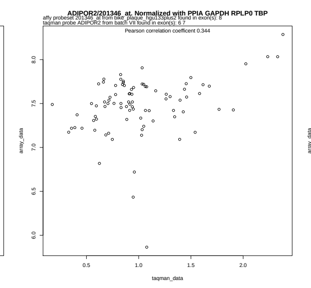
ADIPOR2/201346_at. Normalized with PPIA GAPDH RPLP0 TBP
affy probeset 201346_at from bike_plaque_hgu133plus2 found in exon(s): 8
taqman probe ADIPOR2 from batch VII found in exon(s): 6 7
Pearson correlation coefficent 0.344
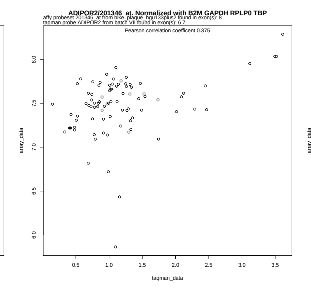
ADIPOR2/201346_at. Normalized with B2M GAPDH RPLP0 TBP
affy probeset 201346_at from bike_plaque_hgu133plus2 found in exon(s): 8
taqman probe ADIPOR2 from batch VII found in exon(s): 6 7
Pearson correlation coefficent 0.375
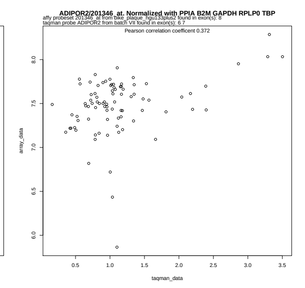
ADIPOR2/201346_at. Normalized with PPIA B2M GAPDH RPLP0 TBP
affy probeset 201346_at from bike_plaque_hgu133plus2 found in exon(s): 8
taqman probe ADIPOR2 from batch VII found in exon(s): 6 7
Pearson correlation coefficent 0.372
