# Supplementary material for: Endogenous control genes in complex vascular tissue samples
Source: BMC Genomics. 2009 Nov 10;10:516. doi: 10.1186/1471-2164-10-516 (PMC2779820; doi:10.1186/1471-2164-10-516)
Supplement: Additional file 1 — scatter plots of real-time PCR and array data for all genes of interest. The zip-file contains pdf files for each gene of interest. Each row in the pdf file shows a probe set investigating the gene in question. Each column in the pdf file shows different combinations of endogenous controls used for preparing the real-time PCR data. [file correlation plots for ALOX12.pdf › correlation plots for ALOX12.pdf]

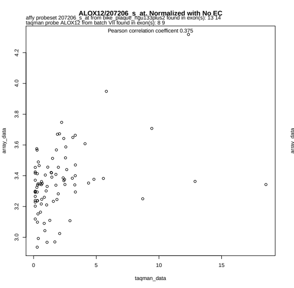
ALOX12/207206_s_at. Normalized with No EC
affy probeset 207206_s_at from bike_plaque_hgu133plus2 found in exon(s): 13 14
taqman probe ALOX12 from batch VII found in exon(s): 8 9
Pearson correlation coefficent 0.375
array_data
taqman_data

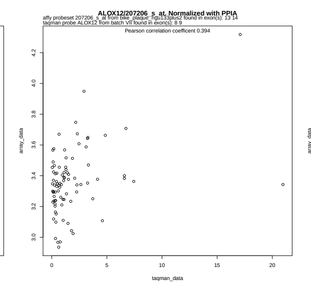
ALOX12/207206_s_at. Normalized with PPIA
affy probeset 207206_s_at from bike_plaque_hgu133plus2 found in exon(s): 13 14
taqman probe ALOX12 from batch VII found in exon(s): 8 9
Pearson correlation coefficent 0.394
array_data
taqman_data

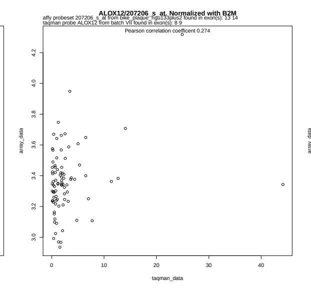
ALOX12/207206_s_at. Normalized with B2M
affy probeset 207206_s_at from bike_plaque_hgu133plus2 found in exon(s): 13 14
taqman probe ALOX12 from batch VII found in exon(s): 8 9
Pearson correlation coefficent 0.274
array_data
taqman_data

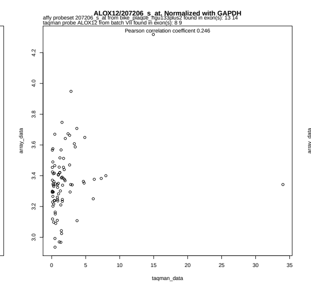
ALOX12/207206_s_at. Normalized with GAPDH
affy probeset 207206_s_at from bike_plaque_hgu133plus2 found in exon(s): 13 14
taqman probe ALOX12 from batch VII found in exon(s): 8 9
Pearson correlation coefficent 0.246
array_data
taqman_data

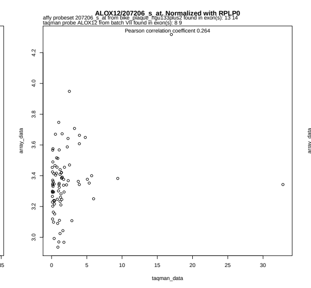
ALOX12/207206_s_at. Normalized with RPLP0
affy probeset 207206_s_at from bike_plaque_hgu133plus2 found in exon(s): 13 14
taqman probe ALOX12 from batch VII found in exon(s): 8 9
Pearson correlation coefficent 0.264
array_data
taqman_data

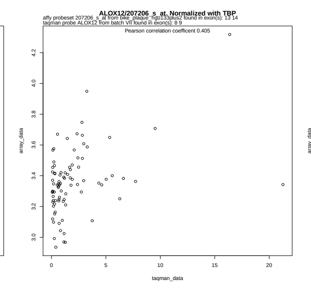
ALOX12/207206_s_at. Normalized with TBP
affy probeset 207206_s_at from bike_plaque_hgu133plus2 found in exon(s): 13 14
taqman probe ALOX12 from batch VII found in exon(s): 8 9
Pearson correlation coefficent 0.405
array_data
taqman_data

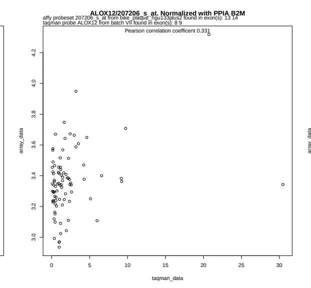
ALOX12/207206_s_at. Normalized with PPIA B2M
affy probeset 207206_s_at from bike_plaque_hgu133plus2 found in exon(s): 13 14
taqman probe ALOX12 from batch VII found in exon(s): 8 9
Pearson correlation coefficent 0.331
array_data
taqman_data

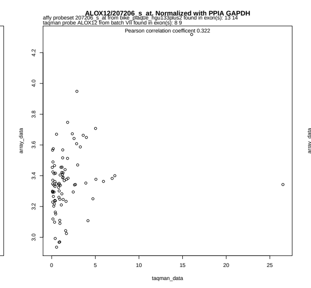
ALOX12/207206_s_at. Normalized with PPIA GAPDH
affy probeset 207206_s_at from bike_plaque_hgu133plus2 found in exon(s): 13 14
taqman probe ALOX12 from batch VII found in exon(s): 8 9
Pearson correlation coefficent 0.322
array_data
taqman_data

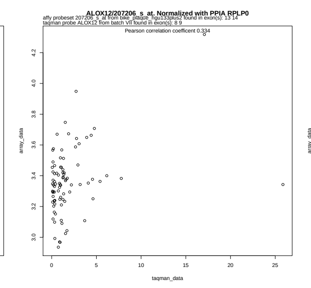
ALOX12/207206_s_at. Normalized with PPIA RPLP0
affy probeset 207206_s_at from bike_plaque_hgu133plus2 found in exon(s): 13 14
taqman probe ALOX12 from batch VII found in exon(s): 8 9
Pearson correlation coefficent 0.334
array_data
taqman_data

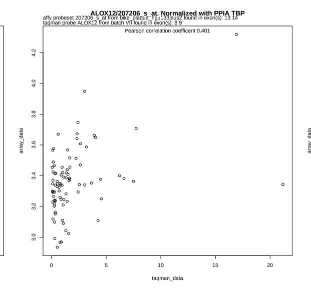
ALOX12/207206_s_at. Normalized with PPIA TBP
affy probeset 207206_s_at from bike_plaque_hgu133plus2 found in exon(s): 13 14
taqman probe ALOX12 from batch VII found in exon(s): 8 9
Pearson correlation coefficent 0.401
array_data
taqman_data

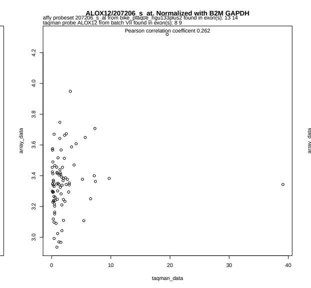
ALOX12/207206_s_at. Normalized with B2M GAPDH
affy probeset 207206_s_at from bike_plaque_hgu133plus2 found in exon(s): 13 14
taqman probe ALOX12 from batch VII found in exon(s): 8 9
Pearson correlation coefficent 0.262
array_data
taqman_data

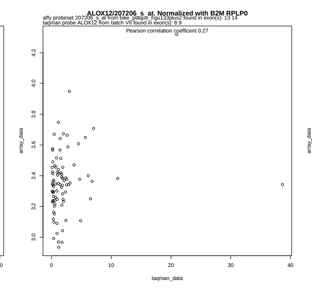
ALOX12/207206_s_at. Normalized with B2M RPLP0
affy probeset 207206_s_at from bike_plaque_hgu133plus2 found in exon(s): 13 14
taqman probe ALOX12 from batch VII found in exon(s): 8 9
Pearson correlation coefficent 0.27
array_data
taqman_data

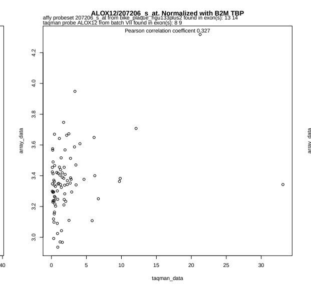
ALOX12/207206_s_at. Normalized with B2M TBP
affy probeset 207206_s_at from bike_plaque_hgu133plus2 found in exon(s): 13 14
taqman probe ALOX12 from batch VII found in exon(s): 8 9
Pearson correlation coefficent 0.327
array_data
taqman_data

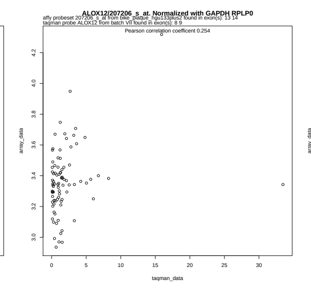
ALOX12/207206_s_at. Normalized with GAPDH RPLP0
affy probeset 207206_s_at from bike_plaque_hgu133plus2 found in exon(s): 13 14
taqman probe ALOX12 from batch VII found in exon(s): 8 9
Pearson correlation coefficent 0.254
array_data
taqman_data

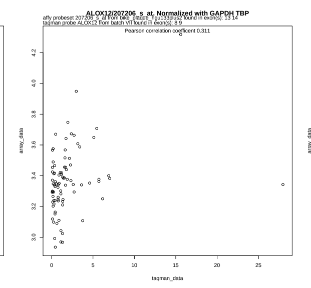
ALOX12/207206_s_at. Normalized with GAPDH TBP
affy probeset 207206_s_at from bike_plaque_hgu133plus2 found in exon(s): 13 14
taqman probe ALOX12 from batch VII found in exon(s): 8 9
Pearson correlation coefficent 0.311
array_data
taqman_data

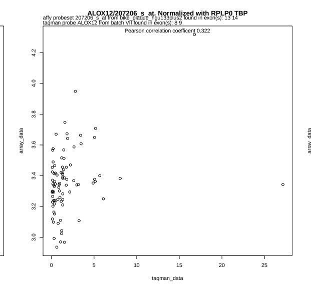
ALOX12/207206_s_at. Normalized with RPLP0 TBP
affy probeset 207206_s_at from bike_plaque_hgu133plus2 found in exon(s): 13 14
taqman probe ALOX12 from batch VII found in exon(s): 8 9
Pearson correlation coefficent 0.322
array_data
taqman_data

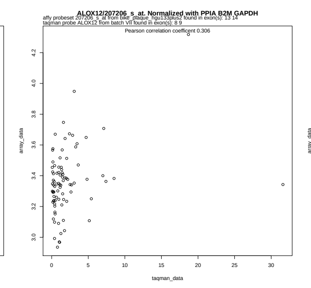
ALOX12/207206_s_at. Normalized with PPIA B2M GAPDH
affy probeset 207206_s_at from bike_plaque_hgu133plus2 found in exon(s): 13 14
taqman probe ALOX12 from batch VII found in exon(s): 8 9
Pearson correlation coefficent 0.306
array_data
taqman_data

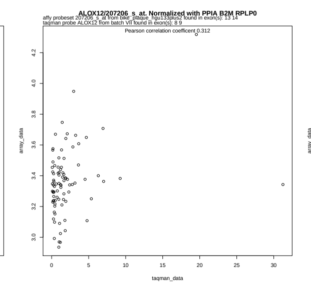
ALOX12/207206_s_at. Normalized with PPIA B2M RPLP0
affy probeset 207206_s_at from bike_plaque_hgu133plus2 found in exon(s): 13 14
taqman probe ALOX12 from batch VII found in exon(s): 8 9
Pearson correlation coefficent 0.312
array_data
taqman_data

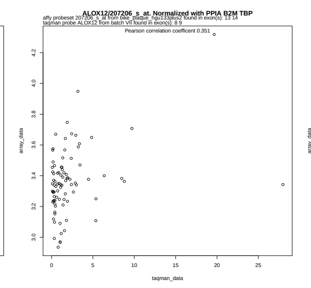
ALOX12/207206_s_at. Normalized with PPIA B2M TBP
affy probeset 207206_s_at from bike_plaque_hgu133plus2 found in exon(s): 13 14
taqman probe ALOX12 from batch VII found in exon(s): 8 9
Pearson correlation coefficent 0.351
array_data
taqman_data

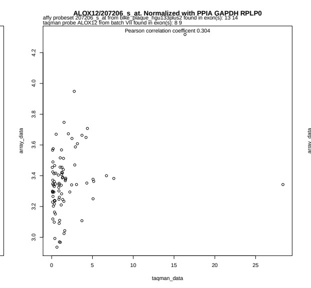
ALOX12/207206_s_at. Normalized with PPIA GAPDH RPLP0
affy probeset 207206_s_at from bike_plaque_hgu133plus2 found in exon(s): 13 14
taqman probe ALOX12 from batch VII found in exon(s): 8 9
Pearson correlation coefficent 0.304
array_data
taqman_data

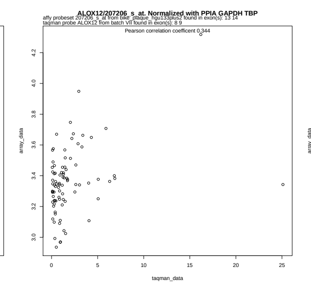
ALOX12/207206_s_at. Normalized with PPIA GAPDH TBP
affy probeset 207206_s_at from bike_plaque_hgu133plus2 found in exon(s): 13 14
taqman probe ALOX12 from batch VII found in exon(s): 8 9
Pearson correlation coefficent 0.344
array_data
taqman_data

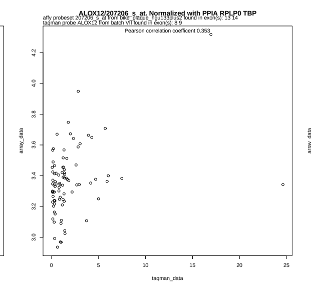
ALOX12/207206_s_at. Normalized with PPIA RPLP0 TBP
affy probeset 207206_s_at from bike_plaque_hgu133plus2 found in exon(s): 13 14
taqman probe ALOX12 from batch VII found in exon(s): 8 9
Pearson correlation coefficent 0.353
array_data
taqman_data

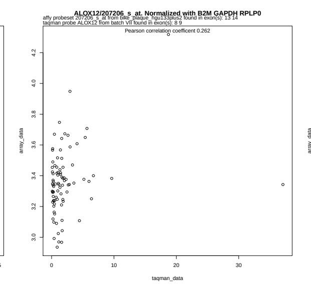
ALOX12/207206_s_at. Normalized with B2M GAPDH RPLP0
affy probeset 207206_s_at from bike_plaque_hgu133plus2 found in exon(s): 13 14
taqman probe ALOX12 from batch VII found in exon(s): 8 9
Pearson correlation coefficent 0.262
array_data
taqman_data

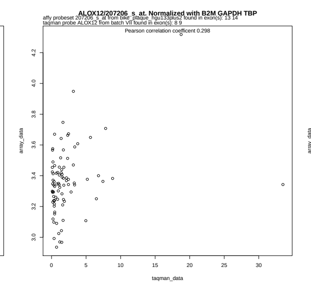
ALOX12/207206_s_at. Normalized with B2M GAPDH TBP
affy probeset 207206_s_at from bike_plaque_hgu133plus2 found in exon(s): 13 14
taqman probe ALOX12 from batch VII found in exon(s): 8 9
Pearson correlation coefficent 0.298
array_data
taqman_data

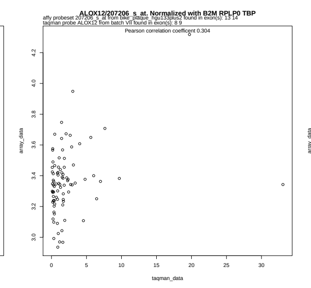
ALOX12/207206_s_at. Normalized with B2M RPLP0 TBP
affy probeset 207206_s_at from bike_plaque_hgu133plus2 found in exon(s): 13 14
taqman probe ALOX12 from batch VII found in exon(s): 8 9
Pearson correlation coefficent 0.304
array_data
taqman_data

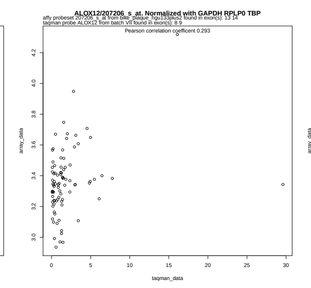
ALOX12/207206_s_at. Normalized with GAPDH RPLP0 TBP
affy probeset 207206_s_at from bike_plaque_hgu133plus2 found in exon(s): 13 14
taqman probe ALOX12 from batch VII found in exon(s): 8 9
Pearson correlation coefficent 0.293
array_data
taqman_data

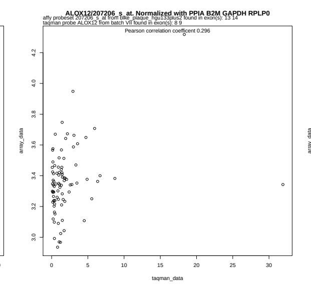
ALOX12/207206_s_at. Normalized with PPIA B2M GAPDH RPLP0
affy probeset 207206_s_at from bike_plaque_hgu133plus2 found in exon(s): 13 14
taqman probe ALOX12 from batch VII found in exon(s): 8 9
Pearson correlation coefficent 0.296
array_data
taqman_data

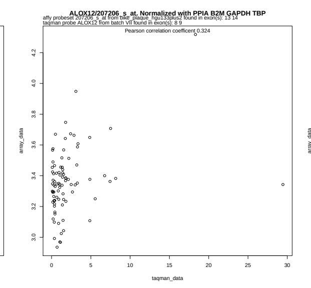
ALOX12/207206_s_at. Normalized with PPIA B2M GAPDH TBP
affy probeset 207206_s_at from bike_plaque_hgu133plus2 found in exon(s): 13 14
taqman probe ALOX12 from batch VII found in exon(s): 8 9
Pearson correlation coefficent 0.324
array_data
taqman_data

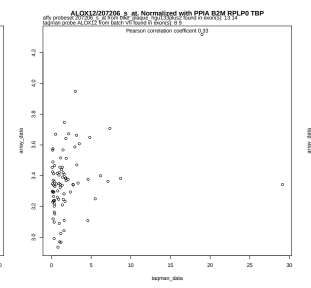
ALOX12/207206_s_at. Normalized with PPIA B2M RPLP0 TBP
affy probeset 207206_s_at from bike_plaque_hgu133plus2 found in exon(s): 13 14
taqman probe ALOX12 from batch VII found in exon(s): 8 9
Pearson correlation coefficent 0.33
array_data
taqman_data

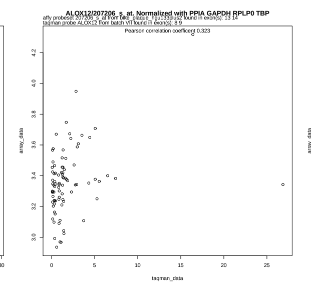
ALOX12/207206_s_at. Normalized with PPIA GAPDH RPLP0 TBP
affy probeset 207206_s_at from bike_plaque_hgu133plus2 found in exon(s): 13 14
taqman probe ALOX12 from batch VII found in exon(s): 8 9
Pearson correlation coefficent 0.323
array_data
taqman_data

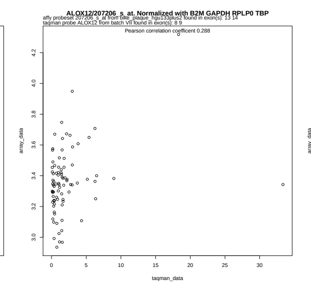
ALOX12/207206_s_at. Normalized with B2M GAPDH RPLP0 TBP
affy probeset 207206_s_at from bike_plaque_hgu133plus2 found in exon(s): 13 14
taqman probe ALOX12 from batch VII found in exon(s): 8 9
Pearson correlation coefficent 0.288
array_data
taqman_data

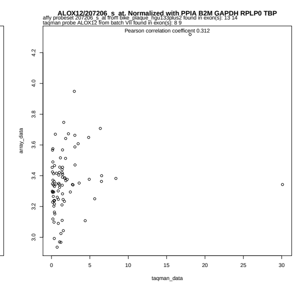
ALOX12/207206_s_at. Normalized with PPIA B2M GAPDH RPLP0 TBP
affy probeset 207206_s_at from bike_plaque_hgu133plus2 found in exon(s): 13 14
taqman probe ALOX12 from batch VII found in exon(s): 8 9
Pearson correlation coefficent 0.312
array_data
taqman_data
