# Supplementary material for: Endogenous control genes in complex vascular tissue samples
Source: BMC Genomics. 2009 Nov 10;10:516. doi: 10.1186/1471-2164-10-516 (PMC2779820; doi:10.1186/1471-2164-10-516)
Supplement: Additional file 1 — scatter plots of real-time PCR and array data for all genes of interest. The zip-file contains pdf files for each gene of interest. Each row in the pdf file shows a probe set investigating the gene in question. Each column in the pdf file shows different combinations of endogenous controls used for preparing the real-time PCR data. [file correlation plots for CD36.pdf › correlation plots for CD36.pdf]

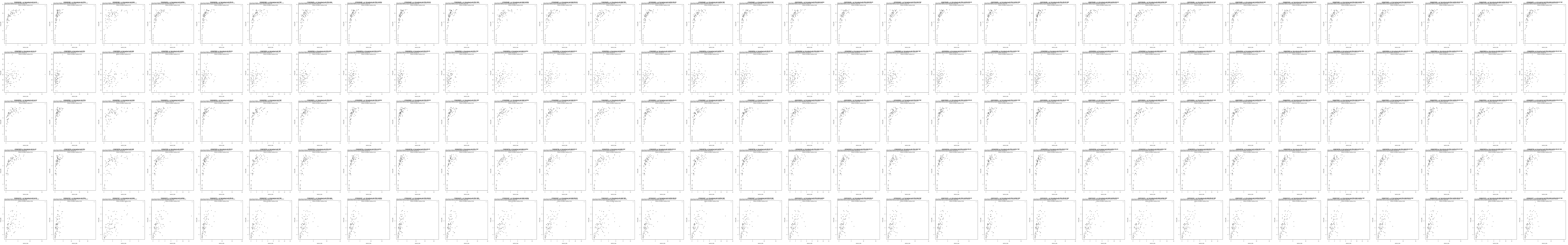
CD36/206488_s_at. Normalized with No EC
affy probeset 206488_s_at from bike_plaque_hgu133plus2 found in exon(s): 12 13 14
taqman probe CD36 from batch VII found in exon(s): 8 9
Pearson correlation coefficent 0.617
array_data
taqman_data
CD36/206488_s_at. Normalized with PPIA
Pearson correlation coefficent 0.419
CD36/206488_s_at. Normalized with B2M
Pearson correlation coefficent 0.579
CD36/209554_at. Normalized with No EC
affy probeset 209554_at from bike_plaque_hgu133plus2 found in exon(s): 15
Pearson correlation coefficent 0.26
CD36/209554_at. Normalized with PPIA
Pearson correlation coefficent 0.222
CD36/209554_at. Normalized with B2M
Pearson correlation coefficent 0.277
