# Supplementary material for: Endogenous control genes in complex vascular tissue samples
Source: BMC Genomics. 2009 Nov 10;10:516. doi: 10.1186/1471-2164-10-516 (PMC2779820; doi:10.1186/1471-2164-10-516)
Supplement: Additional file 1 — scatter plots of real-time PCR and array data for all genes of interest. The zip-file contains pdf files for each gene of interest. Each row in the pdf file shows a probe set investigating the gene in question. Each column in the pdf file shows different combinations of endogenous controls used for preparing the real-time PCR data. [file correlation plots for EDNRB.pdf › correlation plots for EDNRB.pdf]

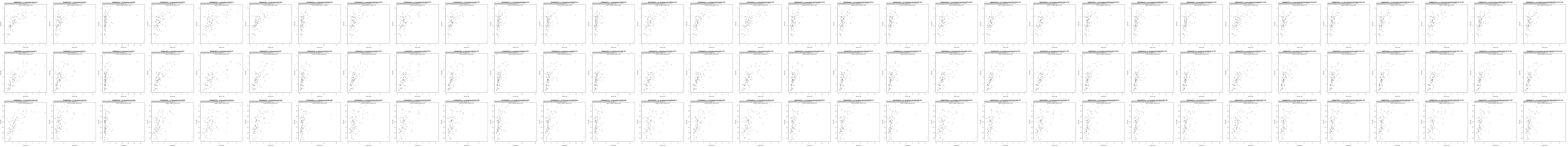
EDNRB/204271_s_at. Normalized with No EC
affy probeset 204271_s_at from bike_plaque_hgu133plus2 found in exon(s): 8
taqman probe EDNRB from batch VII found in exon(s): 7 8
Pearson correlation coefficent 0.474
EDNRB/204271_s_at. Normalized with PPIA
Pearson correlation coefficent 0.151
EDNRB/204271_s_at. Normalized with B2M
Pearson correlation coefficent −0.083
EDNRB/204271_s_at. Normalized with GAPDH
Pearson correlation coefficent 0.25
array_data
taqman_data
