# Supplementary material for: Endogenous control genes in complex vascular tissue samples
Source: BMC Genomics. 2009 Nov 10;10:516. doi: 10.1186/1471-2164-10-516 (PMC2779820; doi:10.1186/1471-2164-10-516)
Supplement: Additional file 1 — scatter plots of real-time PCR and array data for all genes of interest. The zip-file contains pdf files for each gene of interest. Each row in the pdf file shows a probe set investigating the gene in question. Each column in the pdf file shows different combinations of endogenous controls used for preparing the real-time PCR data. [file correlation plots for FABP4.pdf › correlation plots for FABP4.pdf]

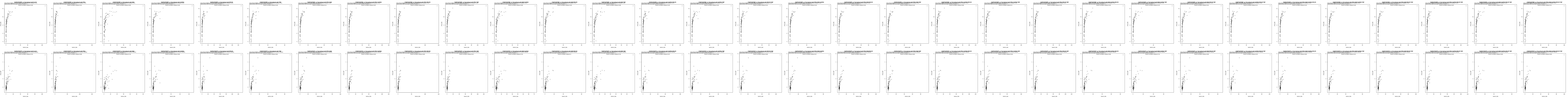
FABP4/203980_at. Normalized with No EC
affy probeset 203980_at from bike_plaque_hgu133plus2 found in exon(s): 1 2 3 4
taqman probe FABP4 from batch VII found in exon(s): 1 2
Pearson correlation coefficent 0.458
FABP4/203980_at. Normalized with PPIA
Pearson correlation coefficent 0.341
FABP4/203980_at. Normalized with B2M
Pearson correlation coefficent 0.509
FABP4/235978_at. Normalized with No EC
affy probeset 235978_at from bike_plaque_hgu133plus2 found in exon(s): Unknown
FABP4/235978_at. Normalized with PPIA
FABP4/235978_at. Normalized with B2M
array_data
taqman_data
