# Supplementary material for: Endogenous control genes in complex vascular tissue samples
Source: BMC Genomics. 2009 Nov 10;10:516. doi: 10.1186/1471-2164-10-516 (PMC2779820; doi:10.1186/1471-2164-10-516)

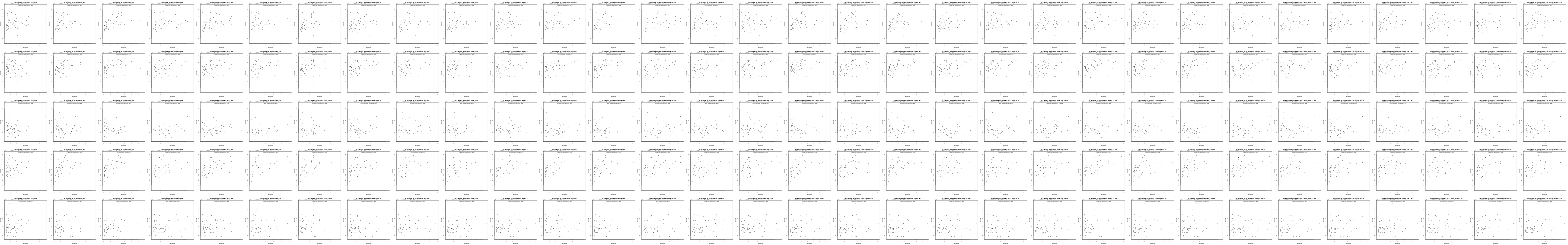

Supplement: Additional file 1 — scatter plots of real-time PCR and array data for all genes of interest. The zip-file contains pdf files for each gene of interest. Each row in the pdf file shows a probe set investigating the gene in question. Each column in the pdf file shows different combinations of endogenous controls used for preparing the real-time PCR data. [file correlation plots for IGF1R.pdf › correlation plots for IGF1R.pdf]
