# Supplementary material for: Endogenous control genes in complex vascular tissue samples
Source: BMC Genomics. 2009 Nov 10;10:516. doi: 10.1186/1471-2164-10-516 (PMC2779820; doi:10.1186/1471-2164-10-516)
Supplement: Additional file 1 — scatter plots of real-time PCR and array data for all genes of interest. The zip-file contains pdf files for each gene of interest. Each row in the pdf file shows a probe set investigating the gene in question. Each column in the pdf file shows different combinations of endogenous controls used for preparing the real-time PCR data. [file correlation plots for IGFBP1.pdf › correlation plots for IGFBP1.pdf]

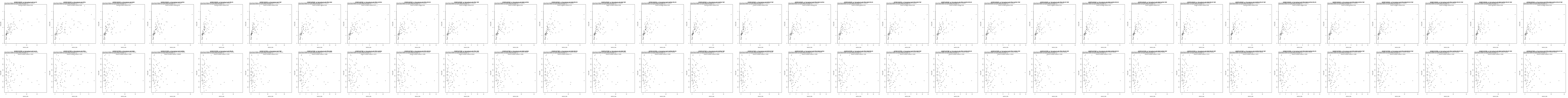
IGFBP1/205302_at. Normalized with No EC
affy probeset 205302_at from bike_plaque_hgu133plus2 found in exon(s): 4
taqman probe IGFBP1 from batch VII found in exon(s): 2 3
Pearson correlation coefficent 0.653
array_data
taqman_data
IGFBP1/205302_at. Normalized with PPIA
affy probeset 205302_at from bike_plaque_hgu133plus2 found in exon(s): 4
taqman probe IGFBP1 from batch VII found in exon(s): 2 3
Pearson correlation coefficent 0.689
array_data
taqman_data
IGFBP1/205302_at. Normalized with B2M
affy probeset 205302_at from bike_plaque_hgu133plus2 found in exon(s): 4
taqman probe IGFBP1 from batch VII found in exon(s): 2 3
Pearson correlation coefficent 0.547
array_data
taqman_data
