# Supplementary material for: Endogenous control genes in complex vascular tissue samples
Source: BMC Genomics. 2009 Nov 10;10:516. doi: 10.1186/1471-2164-10-516 (PMC2779820; doi:10.1186/1471-2164-10-516)
Supplement: Additional file 1 — scatter plots of real-time PCR and array data for all genes of interest. The zip-file contains pdf files for each gene of interest. Each row in the pdf file shows a probe set investigating the gene in question. Each column in the pdf file shows different combinations of endogenous controls used for preparing the real-time PCR data. [file correlation plots for TNFSF4.pdf › correlation plots for TNFSF4.pdf]

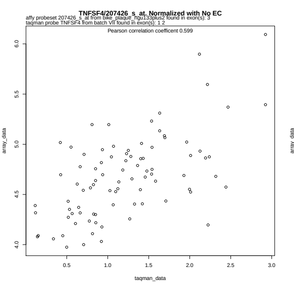
TNFSF4/207426_s_at. Normalized with No EC
affy probeset 207426_s_at from bike_plaque_hgu133plus2 found in exon(s): 3
taqman probe TNFSF4 from batch VII found in exon(s): 1 2
Pearson correlation coefficent 0.599
array_data
taqman_data

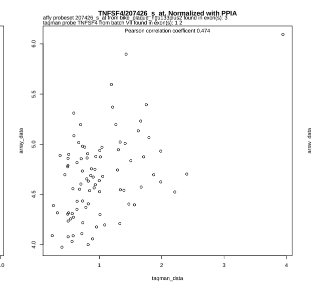
TNFSF4/207426_s_at. Normalized with PPIA
affy probeset 207426_s_at from bike_plaque_hgu133plus2 found in exon(s): 3
taqman probe TNFSF4 from batch VII found in exon(s): 1 2
Pearson correlation coefficent 0.474
array_data
taqman_data

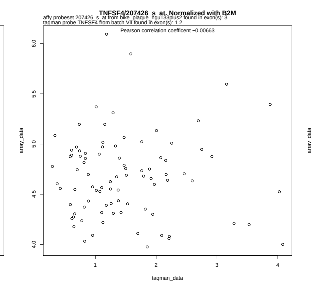
TNFSF4/207426_s_at. Normalized with B2M
affy probeset 207426_s_at from bike_plaque_hgu133plus2 found in exon(s): 3
taqman probe TNFSF4 from batch VII found in exon(s): 1 2
Pearson correlation coefficent −0.00663
array_data
taqman_data

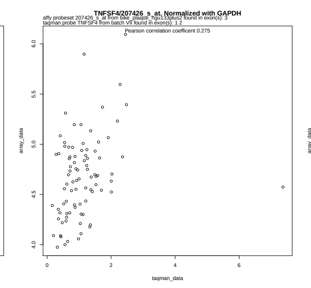
TNFSF4/207426_s_at. Normalized with GAPDH
affy probeset 207426_s_at from bike_plaque_hgu133plus2 found in exon(s): 3
taqman probe TNFSF4 from batch VII found in exon(s): 1 2
Pearson correlation coefficent 0.275
array_data
taqman_data

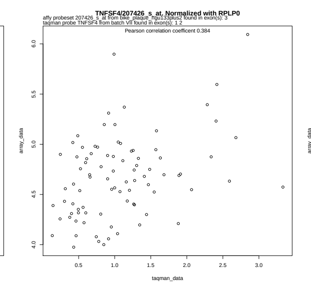
TNFSF4/207426_s_at. Normalized with RPLP0
affy probeset 207426_s_at from bike_plaque_hgu133plus2 found in exon(s): 3
taqman probe TNFSF4 from batch VII found in exon(s): 1 2
Pearson correlation coefficent 0.384
array_data
taqman_data

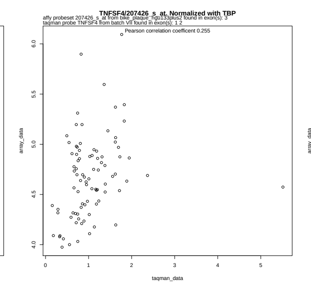
TNFSF4/207426_s_at. Normalized with TBP
affy probeset 207426_s_at from bike_plaque_hgu133plus2 found in exon(s): 3
taqman probe TNFSF4 from batch VII found in exon(s): 1 2
Pearson correlation coefficent 0.255
array_data
taqman_data

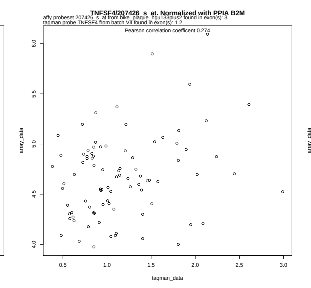
TNFSF4/207426_s_at. Normalized with PPIA B2M
affy probeset 207426_s_at from bike_plaque_hgu133plus2 found in exon(s): 3
taqman probe TNFSF4 from batch VII found in exon(s): 1 2
Pearson correlation coefficent 0.274
array_data
taqman_data

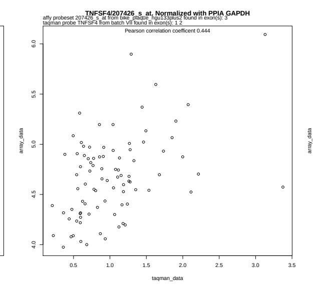
TNFSF4/207426_s_at. Normalized with PPIA GAPDH
affy probeset 207426_s_at from bike_plaque_hgu133plus2 found in exon(s): 3
taqman probe TNFSF4 from batch VII found in exon(s): 1 2
Pearson correlation coefficent 0.444
array_data
taqman_data

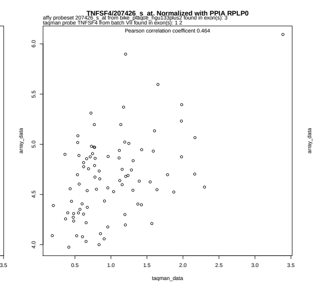
TNFSF4/207426_s_at. Normalized with PPIA RPLP0
affy probeset 207426_s_at from bike_plaque_hgu133plus2 found in exon(s): 3
taqman probe TNFSF4 from batch VII found in exon(s): 1 2
Pearson correlation coefficent 0.464
array_data
taqman_data

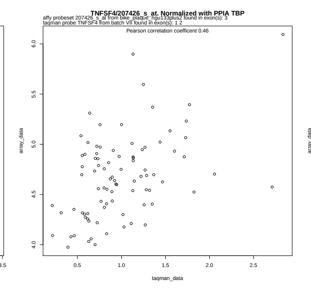
TNFSF4/207426_s_at. Normalized with PPIA TBP
affy probeset 207426_s_at from bike_plaque_hgu133plus2 found in exon(s): 3
taqman probe TNFSF4 from batch VII found in exon(s): 1 2
Pearson correlation coefficent 0.46
array_data
taqman_data

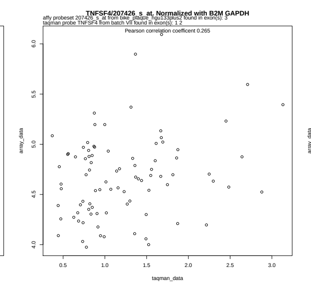
TNFSF4/207426_s_at. Normalized with B2M GAPDH
affy probeset 207426_s_at from bike_plaque_hgu133plus2 found in exon(s): 3
taqman probe TNFSF4 from batch VII found in exon(s): 1 2
Pearson correlation coefficent 0.265
array_data
taqman_data

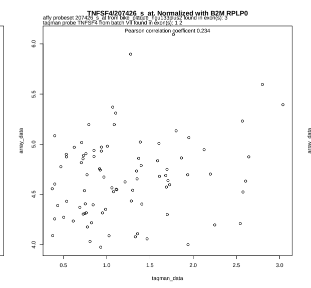
TNFSF4/207426_s_at. Normalized with B2M RPLP0
affy probeset 207426_s_at from bike_plaque_hgu133plus2 found in exon(s): 3
taqman probe TNFSF4 from batch VII found in exon(s): 1 2
Pearson correlation coefficent 0.234
array_data
taqman_data

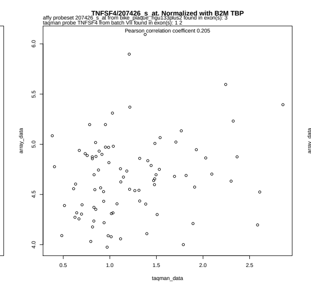
TNFSF4/207426_s_at. Normalized with B2M TBP
affy probeset 207426_s_at from bike_plaque_hgu133plus2 found in exon(s): 3
taqman probe TNFSF4 from batch VII found in exon(s): 1 2
Pearson correlation coefficent 0.205
array_data
taqman_data

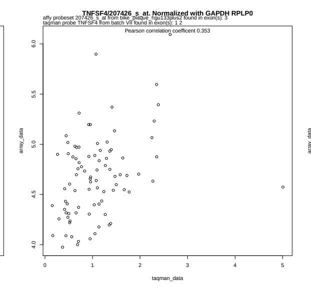
TNFSF4/207426_s_at. Normalized with GAPDH RPLP0
affy probeset 207426_s_at from bike_plaque_hgu133plus2 found in exon(s): 3
taqman probe TNFSF4 from batch VII found in exon(s): 1 2
Pearson correlation coefficent 0.353
array_data
taqman_data

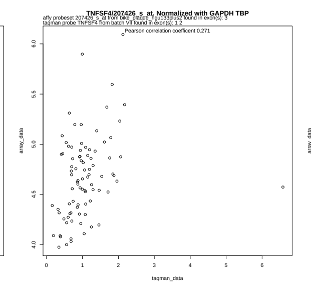
TNFSF4/207426_s_at. Normalized with GAPDH TBP
affy probeset 207426_s_at from bike_plaque_hgu133plus2 found in exon(s): 3
taqman probe TNFSF4 from batch VII found in exon(s): 1 2
Pearson correlation coefficent 0.271
array_data
taqman_data

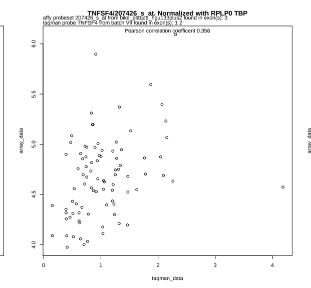
TNFSF4/207426_s_at. Normalized with RPLP0 TBP
affy probeset 207426_s_at from bike_plaque_hgu133plus2 found in exon(s): 3
taqman probe TNFSF4 from batch VII found in exon(s): 1 2
Pearson correlation coefficent 0.356
array_data
taqman_data

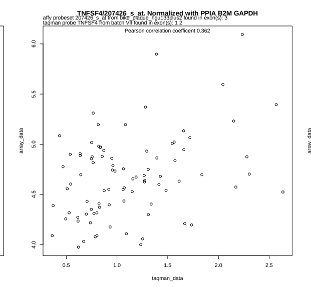
TNFSF4/207426_s_at. Normalized with PPIA B2M GAPDH
affy probeset 207426_s_at from bike_plaque_hgu133plus2 found in exon(s): 3
taqman probe TNFSF4 from batch VII found in exon(s): 1 2
Pearson correlation coefficent 0.362
array_data
taqman_data

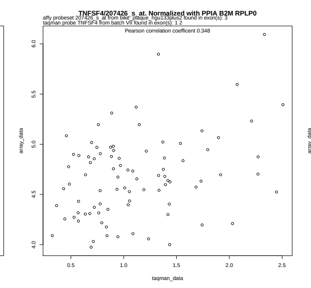
TNFSF4/207426_s_at. Normalized with PPIA B2M RPLP0
affy probeset 207426_s_at from bike_plaque_hgu133plus2 found in exon(s): 3
taqman probe TNFSF4 from batch VII found in exon(s): 1 2
Pearson correlation coefficent 0.348
array_data
taqman_data

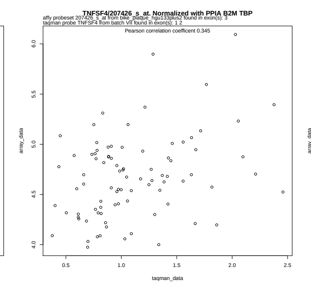
TNFSF4/207426_s_at. Normalized with PPIA B2M TBP
affy probeset 207426_s_at from bike_plaque_hgu133plus2 found in exon(s): 3
taqman probe TNFSF4 from batch VII found in exon(s): 1 2
Pearson correlation coefficent 0.345
array_data
taqman_data

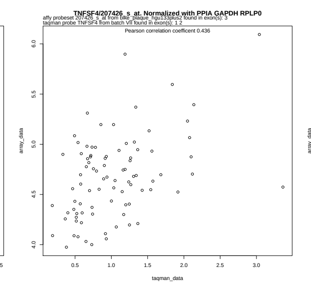
TNFSF4/207426_s_at. Normalized with PPIA GAPDH RPLP0
affy probeset 207426_s_at from bike_plaque_hgu133plus2 found in exon(s): 3
taqman probe TNFSF4 from batch VII found in exon(s): 1 2
Pearson correlation coefficent 0.436
array_data
taqman_data

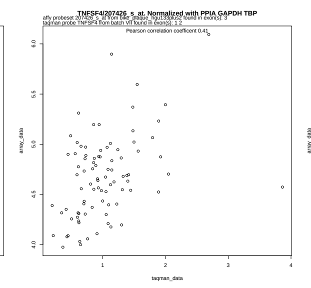
TNFSF4/207426_s_at. Normalized with PPIA GAPDH TBP
affy probeset 207426_s_at from bike_plaque_hgu133plus2 found in exon(s): 3
taqman probe TNFSF4 from batch VII found in exon(s): 1 2
Pearson correlation coefficent 0.41
array_data
taqman_data

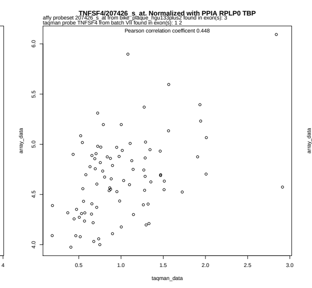
TNFSF4/207426_s_at. Normalized with PPIA RPLP0 TBP
affy probeset 207426_s_at from bike_plaque_hgu133plus2 found in exon(s): 3
taqman probe TNFSF4 from batch VII found in exon(s): 1 2
Pearson correlation coefficent 0.448
array_data
taqman_data

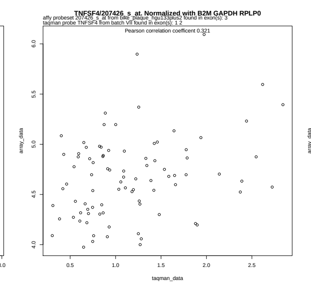
TNFSF4/207426_s_at. Normalized with B2M GAPDH RPLP0
affy probeset 207426_s_at from bike_plaque_hgu133plus2 found in exon(s): 3
taqman probe TNFSF4 from batch VII found in exon(s): 1 2
Pearson correlation coefficent 0.321
array_data
taqman_data

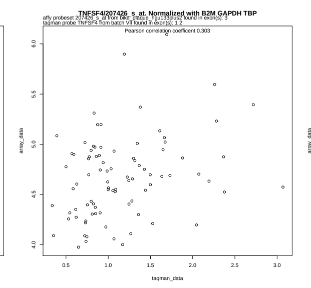
TNFSF4/207426_s_at. Normalized with B2M GAPDH TBP
affy probeset 207426_s_at from bike_plaque_hgu133plus2 found in exon(s): 3
taqman probe TNFSF4 from batch VII found in exon(s): 1 2
Pearson correlation coefficent 0.303
array_data
taqman_data

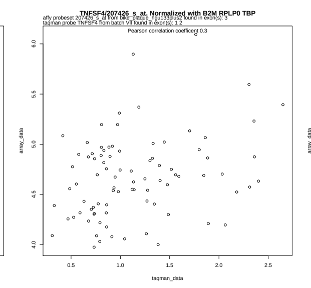
TNFSF4/207426_s_at. Normalized with B2M RPLP0 TBP
affy probeset 207426_s_at from bike_plaque_hgu133plus2 found in exon(s): 3
taqman probe TNFSF4 from batch VII found in exon(s): 1 2
Pearson correlation coefficent 0.3
array_data
taqman_data

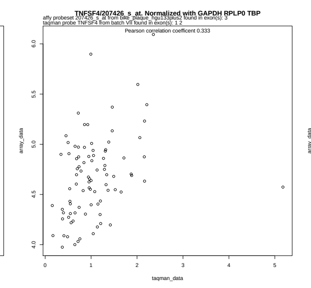
TNFSF4/207426_s_at. Normalized with GAPDH RPLP0 TBP
affy probeset 207426_s_at from bike_plaque_hgu133plus2 found in exon(s): 3
taqman probe TNFSF4 from batch VII found in exon(s): 1 2
Pearson correlation coefficent 0.333
array_data
taqman_data

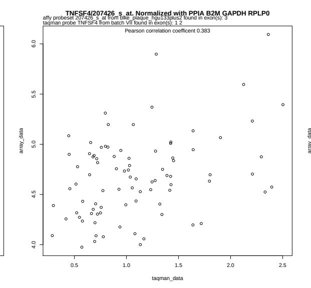
TNFSF4/207426_s_at. Normalized with PPIA B2M GAPDH RPLP0
affy probeset 207426_s_at from bike_plaque_hgu133plus2 found in exon(s): 3
taqman probe TNFSF4 from batch VII found in exon(s): 1 2
Pearson correlation coefficent 0.383
array_data
taqman_data

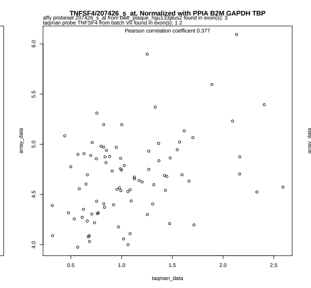
TNFSF4/207426_s_at. Normalized with PPIA B2M GAPDH TBP
affy probeset 207426_s_at from bike_plaque_hgu133plus2 found in exon(s): 3
taqman probe TNFSF4 from batch VII found in exon(s): 1 2
Pearson correlation coefficent 0.377
array_data
taqman_data

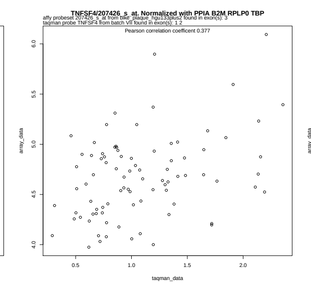
TNFSF4/207426_s_at. Normalized with PPIA B2M RPLP0 TBP
affy probeset 207426_s_at from bike_plaque_hgu133plus2 found in exon(s): 3
taqman probe TNFSF4 from batch VII found in exon(s): 1 2
Pearson correlation coefficent 0.377
array_data
taqman_data

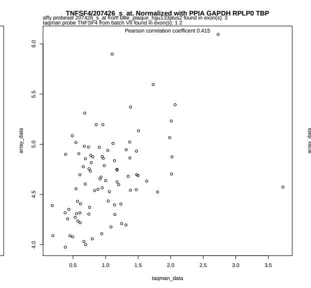
TNFSF4/207426_s_at. Normalized with PPIA GAPDH RPLP0 TBP
affy probeset 207426_s_at from bike_plaque_hgu133plus2 found in exon(s): 3
taqman probe TNFSF4 from batch VII found in exon(s): 1 2
Pearson correlation coefficent 0.415
array_data
taqman_data

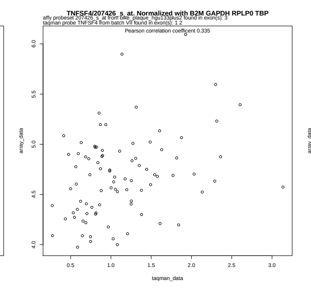
TNFSF4/207426_s_at. Normalized with B2M GAPDH RPLP0 TBP
affy probeset 207426_s_at from bike_plaque_hgu133plus2 found in exon(s): 3
taqman probe TNFSF4 from batch VII found in exon(s): 1 2
Pearson correlation coefficent 0.335
array_data
taqman_data

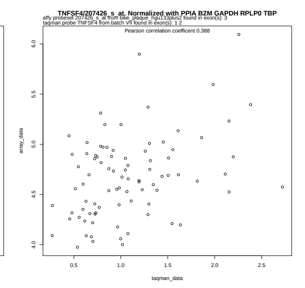
TNFSF4/207426_s_at. Normalized with PPIA B2M GAPDH RPLP0 TBP
affy probeset 207426_s_at from bike_plaque_hgu133plus2 found in exon(s): 3
taqman probe TNFSF4 from batch VII found in exon(s): 1 2
Pearson correlation coefficent 0.388
array_data
taqman_data
