# Supplementary material for: Endogenous control genes in complex vascular tissue samples
Source: BMC Genomics. 2009 Nov 10;10:516. doi: 10.1186/1471-2164-10-516 (PMC2779820; doi:10.1186/1471-2164-10-516)
Supplement: Additional file 1 — scatter plots of real-time PCR and array data for all genes of interest. The zip-file contains pdf files for each gene of interest. Each row in the pdf file shows a probe set investigating the gene in question. Each column in the pdf file shows different combinations of endogenous controls used for preparing the real-time PCR data. [file correlation plots for TNFSF9.pdf › correlation plots for TNFSF9.pdf]

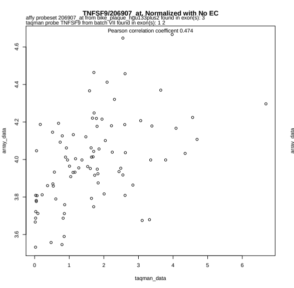
TNFSF9/206907_at. Normalized with No EC
affy probeset 206907_at from bike_plaque_hgu133plus2 found in exon(s): 3
taqman probe TNFSF9 from batch VII found in exon(s): 1 2
Pearson correlation coefficent 0.474
array_data
taqman_data

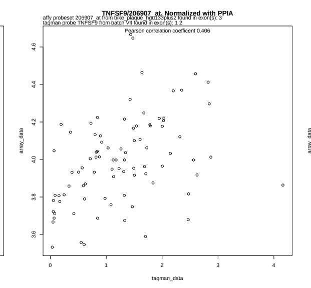
TNFSF9/206907_at. Normalized with PPIA
affy probeset 206907_at from bike_plaque_hgu133plus2 found in exon(s): 3
taqman probe TNFSF9 from batch VII found in exon(s): 1 2
Pearson correlation coefficent 0.406
array_data
taqman_data

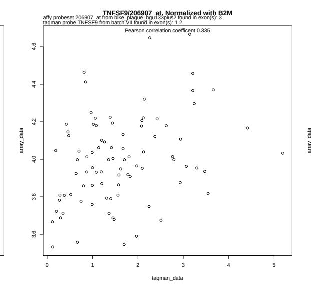
TNFSF9/206907_at. Normalized with B2M
affy probeset 206907_at from bike_plaque_hgu133plus2 found in exon(s): 3
taqman probe TNFSF9 from batch VII found in exon(s): 1 2
Pearson correlation coefficent 0.335
array_data
taqman_data

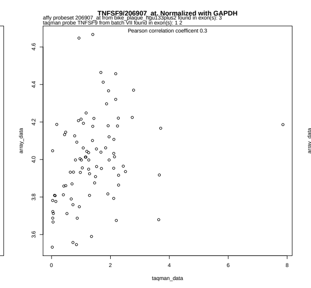
TNFSF9/206907_at. Normalized with GAPDH
affy probeset 206907_at from bike_plaque_hgu133plus2 found in exon(s): 3
taqman probe TNFSF9 from batch VII found in exon(s): 1 2
Pearson correlation coefficent 0.3
array_data
taqman_data

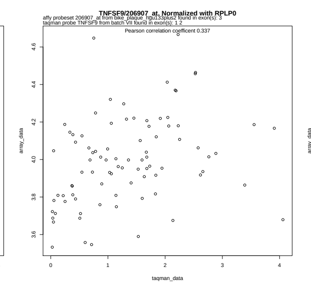
TNFSF9/206907_at. Normalized with RPLP0
affy probeset 206907_at from bike_plaque_hgu133plus2 found in exon(s): 3
taqman probe TNFSF9 from batch VII found in exon(s): 1 2
Pearson correlation coefficent 0.337
array_data
taqman_data

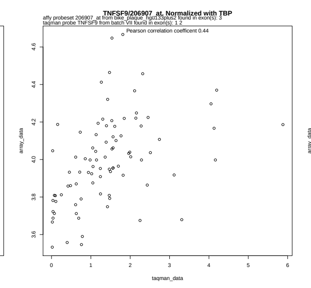
TNFSF9/206907_at. Normalized with TBP
affy probeset 206907_at from bike_plaque_hgu133plus2 found in exon(s): 3
taqman probe TNFSF9 from batch VII found in exon(s): 1 2
Pearson correlation coefficent 0.44
array_data
taqman_data

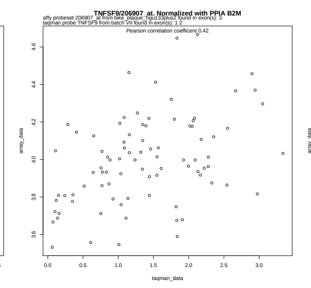
TNFSF9/206907_at. Normalized with PPIA B2M
affy probeset 206907_at from bike_plaque_hgu133plus2 found in exon(s): 3
taqman probe TNFSF9 from batch VII found in exon(s): 1 2
Pearson correlation coefficent 0.42
array_data
taqman_data

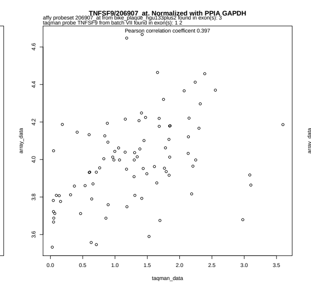
TNFSF9/206907_at. Normalized with PPIA GAPDH
affy probeset 206907_at from bike_plaque_hgu133plus2 found in exon(s): 3
taqman probe TNFSF9 from batch VII found in exon(s): 1 2
Pearson correlation coefficent 0.397
array_data
taqman_data

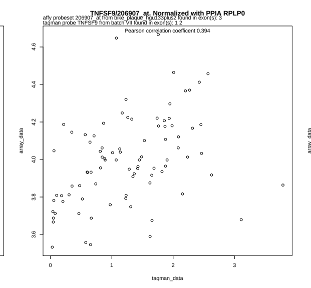
TNFSF9/206907_at. Normalized with PPIA RPLP0
affy probeset 206907_at from bike_plaque_hgu133plus2 found in exon(s): 3
taqman probe TNFSF9 from batch VII found in exon(s): 1 2
Pearson correlation coefficent 0.394
array_data
taqman_data

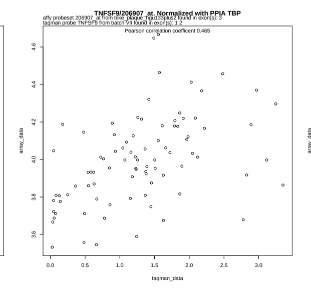
TNFSF9/206907_at. Normalized with PPIA TBP
affy probeset 206907_at from bike_plaque_hgu133plus2 found in exon(s): 3
taqman probe TNFSF9 from batch VII found in exon(s): 1 2
Pearson correlation coefficent 0.465
array_data
taqman_data

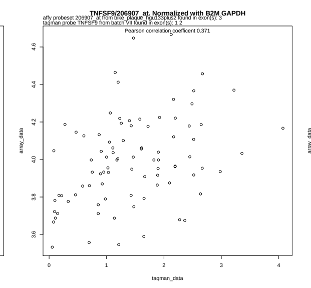
TNFSF9/206907_at. Normalized with B2M GAPDH
affy probeset 206907_at from bike_plaque_hgu133plus2 found in exon(s): 3
taqman probe TNFSF9 from batch VII found in exon(s): 1 2
Pearson correlation coefficent 0.371
array_data
taqman_data

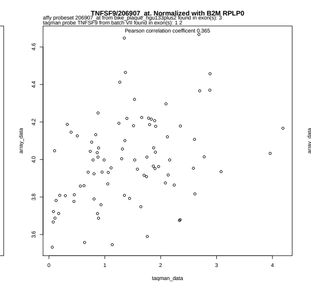
TNFSF9/206907_at. Normalized with B2M RPLP0
affy probeset 206907_at from bike_plaque_hgu133plus2 found in exon(s): 3
taqman probe TNFSF9 from batch VII found in exon(s): 1 2
Pearson correlation coefficent 0.365
array_data
taqman_data

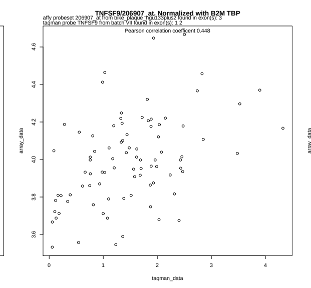
TNFSF9/206907_at. Normalized with B2M TBP
affy probeset 206907_at from bike_plaque_hgu133plus2 found in exon(s): 3
taqman probe TNFSF9 from batch VII found in exon(s): 1 2
Pearson correlation coefficent 0.448
array_data
taqman_data

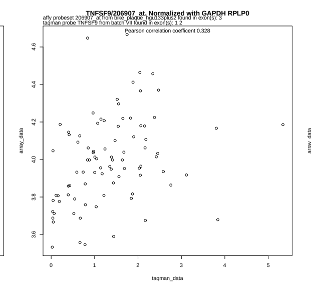
TNFSF9/206907_at. Normalized with GAPDH RPLP0
affy probeset 206907_at from bike_plaque_hgu133plus2 found in exon(s): 3
taqman probe TNFSF9 from batch VII found in exon(s): 1 2
Pearson correlation coefficent 0.328
array_data
taqman_data

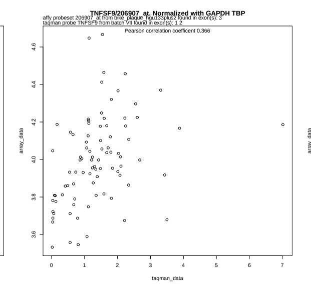
TNFSF9/206907_at. Normalized with GAPDH TBP
affy probeset 206907_at from bike_plaque_hgu133plus2 found in exon(s): 3
taqman probe TNFSF9 from batch VII found in exon(s): 1 2
Pearson correlation coefficent 0.366
array_data
taqman_data

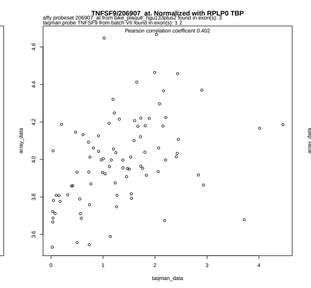
TNFSF9/206907_at. Normalized with RPLP0 TBP
affy probeset 206907_at from bike_plaque_hgu133plus2 found in exon(s): 3
taqman probe TNFSF9 from batch VII found in exon(s): 1 2
Pearson correlation coefficent 0.402
array_data
taqman_data

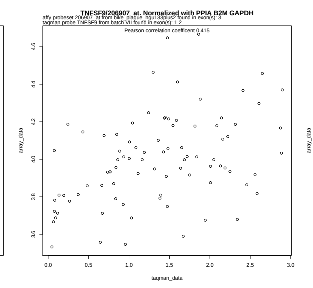
TNFSF9/206907_at. Normalized with PPIA B2M GAPDH
affy probeset 206907_at from bike_plaque_hgu133plus2 found in exon(s): 3
taqman probe TNFSF9 from batch VII found in exon(s): 1 2
Pearson correlation coefficent 0.415
array_data
taqman_data

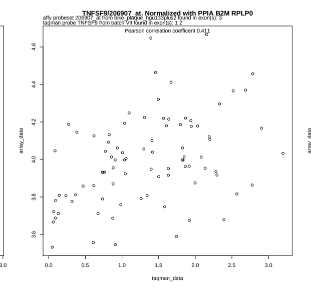
TNFSF9/206907_at. Normalized with PPIA B2M RPLP0
affy probeset 206907_at from bike_plaque_hgu133plus2 found in exon(s): 3
taqman probe TNFSF9 from batch VII found in exon(s): 1 2
Pearson correlation coefficent 0.411
array_data
taqman_data

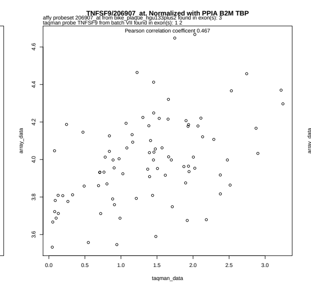
TNFSF9/206907_at. Normalized with PPIA B2M TBP
affy probeset 206907_at from bike_plaque_hgu133plus2 found in exon(s): 3
taqman probe TNFSF9 from batch VII found in exon(s): 1 2
Pearson correlation coefficent 0.467
array_data
taqman_data

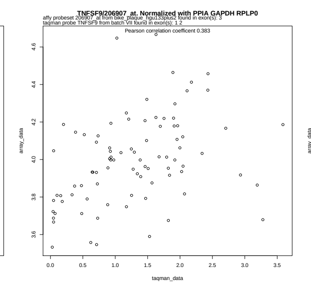
TNFSF9/206907_at. Normalized with PPIA GAPDH RPLP0
affy probeset 206907_at from bike_plaque_hgu133plus2 found in exon(s): 3
taqman probe TNFSF9 from batch VII found in exon(s): 1 2
Pearson correlation coefficent 0.383
array_data
taqman_data

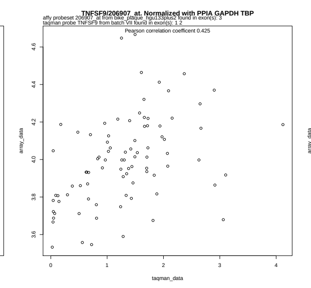
TNFSF9/206907_at. Normalized with PPIA GAPDH TBP
affy probeset 206907_at from bike_plaque_hgu133plus2 found in exon(s): 3
taqman probe TNFSF9 from batch VII found in exon(s): 1 2
Pearson correlation coefficent 0.425
array_data
taqman_data

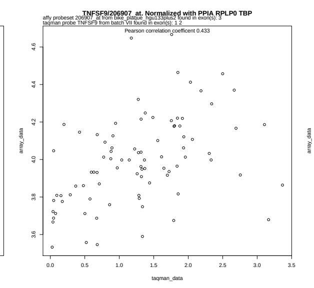
TNFSF9/206907_at. Normalized with PPIA RPLP0 TBP
affy probeset 206907_at from bike_plaque_hgu133plus2 found in exon(s): 3
taqman probe TNFSF9 from batch VII found in exon(s): 1 2
Pearson correlation coefficent 0.433
array_data
taqman_data

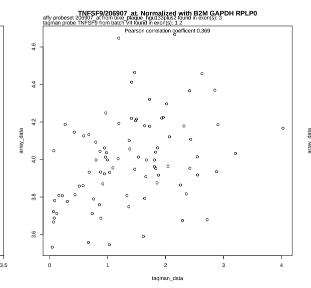
TNFSF9/206907_at. Normalized with B2M GAPDH RPLP0
affy probeset 206907_at from bike_plaque_hgu133plus2 found in exon(s): 3
taqman probe TNFSF9 from batch VII found in exon(s): 1 2
Pearson correlation coefficent 0.369
array_data
taqman_data

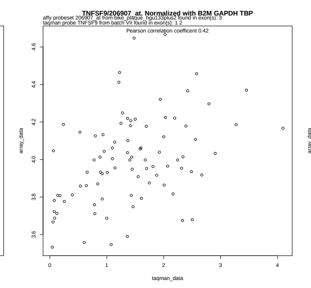
TNFSF9/206907_at. Normalized with B2M GAPDH TBP
affy probeset 206907_at from bike_plaque_hgu133plus2 found in exon(s): 3
taqman probe TNFSF9 from batch VII found in exon(s): 1 2
Pearson correlation coefficent 0.42
array_data
taqman_data

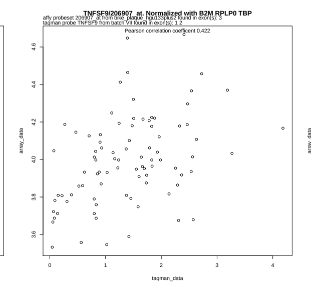
TNFSF9/206907_at. Normalized with B2M RPLP0 TBP
affy probeset 206907_at from bike_plaque_hgu133plus2 found in exon(s): 3
taqman probe TNFSF9 from batch VII found in exon(s): 1 2
Pearson correlation coefficent 0.422
array_data
taqman_data

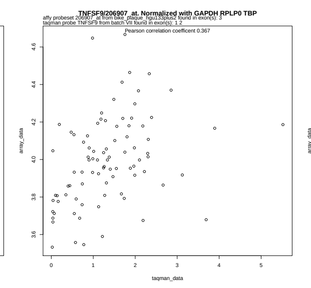
TNFSF9/206907_at. Normalized with GAPDH RPLP0 TBP
affy probeset 206907_at from bike_plaque_hgu133plus2 found in exon(s): 3
taqman probe TNFSF9 from batch VII found in exon(s): 1 2
Pearson correlation coefficent 0.367
array_data
taqman_data

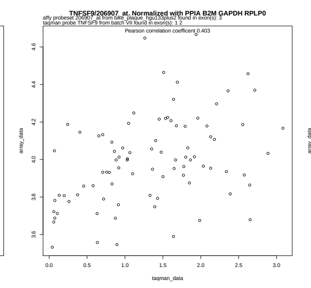
TNFSF9/206907_at. Normalized with PPIA B2M GAPDH RPLP0
affy probeset 206907_at from bike_plaque_hgu133plus2 found in exon(s): 3
taqman probe TNFSF9 from batch VII found in exon(s): 1 2
Pearson correlation coefficent 0.403
array_data
taqman_data

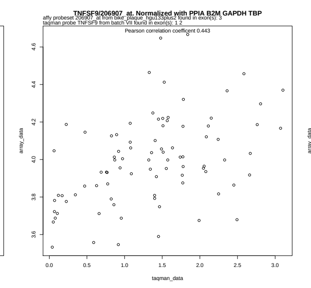
TNFSF9/206907_at. Normalized with PPIA B2M GAPDH TBP
affy probeset 206907_at from bike_plaque_hgu133plus2 found in exon(s): 3
taqman probe TNFSF9 from batch VII found in exon(s): 1 2
Pearson correlation coefficent 0.443
array_data
taqman_data

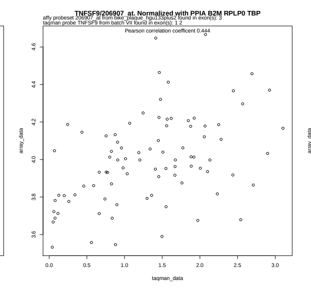
TNFSF9/206907_at. Normalized with PPIA B2M RPLP0 TBP
affy probeset 206907_at from bike_plaque_hgu133plus2 found in exon(s): 3
taqman probe TNFSF9 from batch VII found in exon(s): 1 2
Pearson correlation coefficent 0.444
array_data
taqman_data

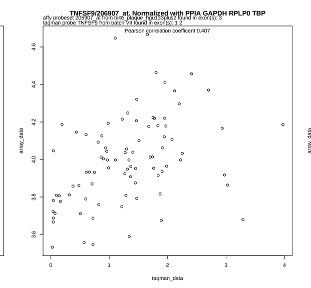
TNFSF9/206907_at. Normalized with PPIA GAPDH RPLP0 TBP
affy probeset 206907_at from bike_plaque_hgu133plus2 found in exon(s): 3
taqman probe TNFSF9 from batch VII found in exon(s): 1 2
Pearson correlation coefficent 0.407
array_data
taqman_data

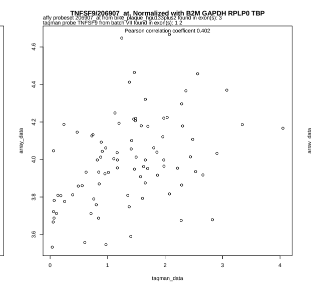
TNFSF9/206907_at. Normalized with B2M GAPDH RPLP0 TBP
affy probeset 206907_at from bike_plaque_hgu133plus2 found in exon(s): 3
taqman probe TNFSF9 from batch VII found in exon(s): 1 2
Pearson correlation coefficent 0.402
array_data
taqman_data

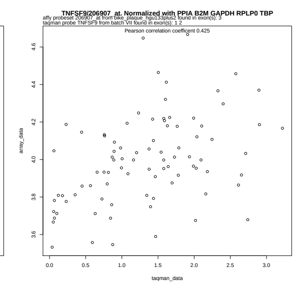
TNFSF9/206907_at. Normalized with PPIA B2M GAPDH RPLP0 TBP
affy probeset 206907_at from bike_plaque_hgu133plus2 found in exon(s): 3
taqman probe TNFSF9 from batch VII found in exon(s): 1 2
Pearson correlation coefficent 0.425
array_data
taqman_data
